# Primary care diagnosed multimorbidity and risk of venous thromboembolism in Sweden

**DOI:** 10.1016/j.ijcrp.2026.200631

**Published:** 2026-04-05

**Authors:** Jonatan Ahrén, MirNabi Pirouzifard, Björn Holmquist, Jan Sundquist, Kristina Sundquist, Bengt Zöller

**Affiliations:** a Center for Primary Health Care Research, Lund University/Region Skåne, 205 02, Malmö, Sweden; b University Clinic Primary Care, Skåne University Hospital, Region Skåne, Sweden; c Department of Statistics, Lund University, 220 07, Lund, Sweden

**Keywords:** Primary care, Epidemiology, Medicine, Multimorbidity, Public health, Venous thromboembolism

## Abstract

**Background:**

Multimorbidity, the co-occurrence of two or more non-communicable diseases (NCDs), is common and has been linked to venous thromboembolism (VTE). Whether multimorbidity diagnosed in primary-care is associated with incident VTE remains unclear. We aimed to examine this association.

**Methods:**

Multimorbidity was defined using Swedish primary-care data. Individuals without prior VTE were included, and incident VTE was identified through the Swedish National Patient Register. Primary-care multimorbidity was defined as two or more NCDs. Subdistribution hazard ratios (subHRs) for VTE were estimated adjusting for sociodemographic factors and acquired and inherited VTE risk factors. The VTE risk of nine disease clusters were investigated.

**Results:**

Among 8,170,329 included individuals, 2,183,236 (26.72%) had primary care-diagnosed multimorbidity. Adjusted subHR for VTE among individuals with multimorbidity was 1.36 (95%CI 1.34-1.39). A dose-response association was observed, a subHR of 1.62 (95%CI 1.57-1.67) for individuals with ≥5 NCDs. There were significant interactions between multimorbidity and sex and country of birth. Seven of nine multimorbidity clusters were associated with increased VTE risk.

**Conclusion:**

Primary care-diagnosed multimorbidity is an independent, dose-dependent risk factor for VTE. The association between several disease-clusters and VTE suggests potential value in cluster-based risk prediction.

## Highlights

Few studies have examined multimorbidity as a risk factor for VTE.
Primary care-diagnosed multimorbidity is linked to higher VTE risk.
VTE risk increases with increasing multimorbidity severity.
Findings may suggest a role for multimorbidity in future VTE risk assessment.

## 1 Introduction

Multimorbidity is a growing global healthcare challenge [1,2]. It is usually defined as the co-occurrence of two or more chronic conditions. Although often referring to non-communicable diseases (NCDs), it can also include long-term infectious diseases [[1], [2], [3]]. The presence of multimorbidity is associated with increased mortality, reduced quality of life, and higher demands on healthcare services and resources [4].

A large Scottish study of 1,751,841 primary-care patients found that 23.2% had multimorbidity [5]. Multimorbidity is a global health burden, increasingly affecting low- and middle-income countries [6]. Several factors are linked to higher multimorbidity risk, including older age, mental health disorders, female sex, low socioeconomic status, residence in socioeconomically disadvantaged areas, smoking, physical inactivity, and elevated body mass index [4,5]. Family history of multimorbidity is also a risk factor for multimorbidity, which suggests a genetic component to multimorbidity [7,8].

Venous thromboembolism (VTE) is the third most common cardiovascular disease, with an incidence of 1-3 cases per 1000 individuals [9]. Incidence increases with age, from around 1 per 100,000 in children to around 1% in individuals >70 years [10]. There is an increase in incidence during the preceding decades [11]. VTE typically presents as deep vein thrombosis (DVT) of the lower extremities or as pulmonary embolism (PE). Clinical outcomes range from post-thrombotic syndrome, affecting approximately 20% of DVT-patients, to acute mortality, which affects 1–2% of individuals with PE [10,12]. The 90-day mortality rate following a diagnosis of VTE is approximately 5% [13]. Risk factors for VTE are both acquired and genetic [14,15].

Recent studies indicate an association between multimorbidity and VTE [[16], [17], [18], [19]]. VTE risk is especially high in individuals with several NCDs [[16], [17], [18], [19]]. Moreover, it has been shown that multimorbidity disease clusters were associated with increased VTE risk [17,19]. These clusters reflect non-random patterns of coexisting NCDs and may capture heterogeneity in VTE risk beyond disease count alone. A nationwide Swedish study of hospital-diagnosed multimorbidity showed a dose-graded increase in VTE risk with hazard ratio (HR) of 2.38 (95%CI 2.12-2.66) for two NCDs and 4.29 (95%CI 2.53-7.28) for five NCDs [19].

The role of primary care-diagnosed multimorbidity in VTE risk has not been studied, although most patients are being managed in primary care.

We used primary-care data covering almost 90% of the Swedish population to examine the association between primary care-diagnosed multimorbidity and incident VTE. Our aim was to assess its importance for VTE risk in Sweden.

## 2 Material and methods

### 2.1 Registers

Data were obtained from Swedish registers, the Cause of Death Register (SCDR), the Total Population Register (education, migration, marital status, and dates of death), the Multigeneration Registers (familial relationships), the National Patient Register (NPR), hospital discharge diagnoses from 1964 to 2018 (with nationwide coverage from 1987) and outpatient hospital diagnoses from 2001 to 2018, and the National Prescribed Drug Register (prescription information, pharmacy dispensing data, medication type and indication for use) were incorporated. Additionally, primary care data from 20 of Sweden's 21 counties, covering almost 90% of the population, were used (Supplementary Tables 1–2). Data were linked using the Swedish personal identity number, which was replaced with a serial number and pseudonymized by Statistics Sweden before delivery. The registers are known for their comprehensive coverage and have been extensively validated in previous studies [[20], [21], [22]]. The data were provided by the National Board of Health and Welfare and Statistics Sweden.

### 2.2 Study design

A register-based cohort study was conducted with a follow-up period from 2010 to 2018, utilizing the landmark approach [19]. To minimize regression dilution bias and immortal time bias, multimorbidity was fixed at two predefined landmark time points selected to represent clinically meaningful intervals in which exposure could be reliably assessed. These landmarks ensured a stable temporal ordering between exposure and outcome. Two landmarks were established, with a five-year interval between baselines: 2010-01-01, and 2015-01-01. Individuals were followed until 2018-12-31, from each respective landmark. Landmark 1 had a follow-up of nine years, while Landmark 2 had a follow-up of four years. Exclusion criteria for all landmarks included VTE before the start of follow-up (i.e. prevalent VTE), death, emigration, and use of Direct Oral Anticoagulants (DOACs), Warfarin, oral contraceptives (OC) or hormone replacement therapy (HRT) at the respective landmark baseline. OC or HRT use were excluded, due to the complexity of modelling these time-varying exposures in the large cohort and due to the lack of formulation-specific information, leading to risk of exposure misclassification. The time periods used to assess prevalent multimorbidity and to exclude individuals prior to the two landmark baselines were 2005–2009 for Landmark 1 and 2010–2014 for Landmark 2. VTE events were assessed for Landmark 1 from 2010 to 2018 and for Landmark 2 2015-2018. The study design is illustrated in a flowchart (Supplementary Figs. 1–2). For individuals under 25 years, education was based on the highest level of education achieved by the individual or their parents. Country of birth was divided into born in Sweden, other Nordic countries, other European countries, and the rest of the world. Landmark 2 includes expanded registry coverage and will be presented in the main article, while Landmark 1 is presented in the supplementary appendix. A directed acyclic graph illustrating the study framework is provided (Supplementary Fig. 3).

### 2.3 Multimorbidity

A list of 45 non-communicable diseases (NCDs), previously published and modified after Barnett et al., was used to define multimorbidity (Supplementary Table 3) [5,8,16,17,19]. These NCDs were identified using International Classification of Diseases, 10th edition (ICD-10) codes from the primary-care data. The included NCDs are common, chronic, and carry a significant disease burden [5,8]. Multimorbidity was defined as the presence of two or more NCDs [8]. A multimorbidity score (1, 2, 3, 4, ≥5) was assigned, with each diagnosis receiving one point, and severity, used here to represent the overall burden of multimorbidity, was assessed based on the total number of diagnoses (0 to ≥5) [8,16,17,19,23].

### 2.4 Definition of the outcome venous thromboembolism

Cases of VTE were identified using the ICD-10 code, I26 to define PE, I80 (excluding I80.0) to define DVT [16,17,19]. These codes were applied to the SCDR and NPR, incorporating main and secondary diagnoses from the NPR. The validity of diagnoses in the NPR is high, with overall diagnostic accuracy estimated at 72-93%, specifically 95% for VTE [21,22,24,25].

### 2.5 Adjusting variables

Adjustments were made for sex, year of birth, education, country of birth, VTE in siblings, Human immunodeficiency virus (HIV) (B20-24), Chronic viral hepatitis B (B18.0-B18.1), Chronic viral hepatitis C (B18.2), Other thrombophilia (D68.6), Activated protein C resistance [factor V Leiden mutation] (D68.5A), Deficiency antithrombin (D68.5B), Deficiency protein C (D68.5C), Deficiency protein S (D68.5D), and Prothrombin gene mutation (D68.5E).

### 2.6 Statistical analysis

The association between multimorbidity and VTE was analysed using competing risk models with death and emigration treated as competing events. Subdistribution hazard ratios (subHR) were estimated using the Fine-Gray model, with 95% confidence intervals (CIs) [26]. Cumulative incidence functions (CIF) were calculated to estimate the probability of VTE occurrence across different levels of multimorbidity. Kaplan–Meier (KM) estimators were generated to show VTE-free survival time. The KM estimator and the CIF are both used in survival analysis, but they differ in how they handle competing risks. KM treats events other than the one of interest as censored, potentially overestimating the risk of the event, while CIF explicitly models the probability of experiencing a specific event while accounting for the possibility of other events occurring first. Incidence rates and incidence-rate ratios were estimated separately for each landmark cohort. SubHRs were stratified by sex, quartiles of birth year, and country of birth [27,28]. Log–log survival curves were examined to assess the proportional hazards assumption. In both landmark cohorts, the curves did not cross and remained parallel throughout the observation period, indicating that the proportional hazards assumption was not violated (Supplementary Fig. 4). Several sensitivity analyses were performed to assess the robustness of the findings. Interaction analyses were performed by including relevant interaction terms in the models. Nine previously defined disease clusters were used to investigate their association with VTE (Supplementary Table 4) [8].

## 3 Results

### 3.1 Multimorbidity

During Landmark 2 (2015-01-01-2018-12-31) a total of 8,170,329 participants were included. Of these 2,183,236 (26.72%) had multimorbidity (Table 1). Table 1 presents the descriptive statistics for study participants after exclusion criteria. Supplementary Tables 5–6 show complete study population for Landmark 1 and 2. The cohort included 42.83% females. Multimorbidity was more common in females (30.27%) than in males (24.06%), and 48.52% of all multimorbid individuals were females. The proportion of females increased with multimorbidity severity, from being 42.83% in the full cohort to 50.73% among individuals with five or more NCDs. Individuals with higher education had a lower prevalence of multimorbidity; 31.36% of individuals with multimorbidity had higher educational attainment. This proportion declined with increasing multimorbidity severity, reaching 21.86% among those with a multimorbidity score of five or more. Individuals with multimorbidity were older, median age of 60 compared to the non-multimorbid individuals, median age of 34 at the end of the study. The age at the end of the study increased gradually with increasing multimorbidity scores.

**Table 1:** Descriptive findings for study participants in Landmark 2.

**Multimorbidity score**									
	All	0	1	2	3	4	≥5	≤1	≥2
Unique individuals, n (%)	8170329 (100)	4338142 (53.10)	1648951 (20.18)	891363 (10.91)	522119 (6.39)	316792 (3.88)	452962 (5.54)	5987093 (73.28)	2183236 (26.72)
Sex, Female, n (%)	3499551 (42.83)	1738785 (49.69)	701500 (20.05)	412836 (11.80)	256619 (7.33)	160003 (4.57)	229808 (6.57)	2440285 (69.73)	1059266 (30.27)
Education (≥12 years), n (%)	3458113 (42.33)	2043542 (47.11)	729841 (44.26)	334449 (37.52)	164370 (31.48)	80895 (27.43)	99016 (21.86)	2733383 (46.32)	684730 (31.36)
Age at end of study, Median (IQR) Range (min-max)	41 (22-60) (0.1-111)	31 (16-49) (0.1-111)	42 (23-58) (1-109)	52 (35-67) (1.5-109)	60 (45-73) (1.5-109)	65 (51-77) (5-109)	72 (59-83) (2-109)	34 (18-52) (0.1-111)	60 (44-74) (1.5-109)
VTE, n (%)	55211 (0.68)	14720 (0.34)	9596 (0.58)	8422 (0.94)	6912 (1.32)	5169 (1.63)	10392 (2.29)	24316 (0.41)	30895 (1.42)

VTE=Venous thromboembolism, IQR=Interquartile Range

### 3.2 Venous thromboembolism

A total of 55,211 incident VTE events were recorded, corresponding to 0.68% of the study population. Among these, 30,895 events (1.42%) occurred in individuals with multimorbidity, compared to 24,316 events (0.41%) in individuals without multimorbidity. The incidence of VTE increased progressively with multimorbidity severity: 0.94% with score of two, 1.32% with score of three, 1.63% with score of four, and 2.29% among those with score of five or more (Table 1).

### 3.3 Multimorbidity and incident VTE

**Fig. 1:**
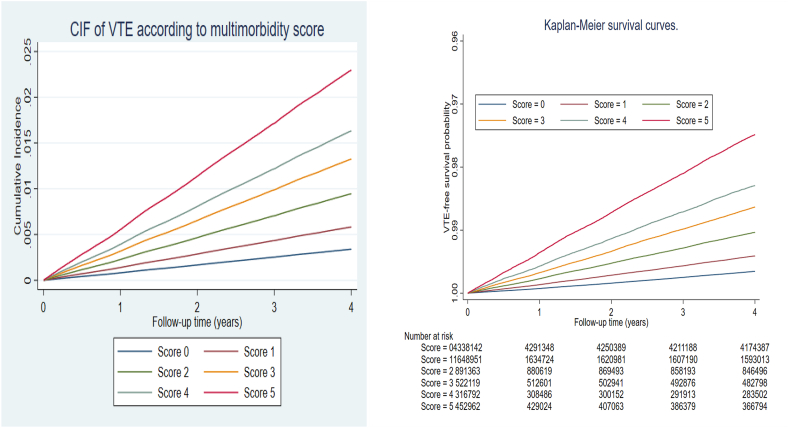
Cumulative incidence function (CIF) of venous thromboembolism (VTE) according to multimorbidity score (score 0 - ≥5) and Kaplan-Meier estimates for VTE-free survival time according to multimorbidity score (score 0 - ≥5) in Landmark 2 with 4 years of follow-up time.

For individuals with multimorbidity, the incidence rate (IR) for VTE was 3.71 (3.67-3.76)/1000 person-years compared to 1.04 (1.03-1.05)/1000 person-years among those without multimorbidity (Table 2). For individuals with multimorbidity severity of ≥5 the IR for VTE was 6.37 (6.25-6.50)/1000 person years. The incidence rate ratio (IRR) for VTE in individuals with multimorbidity was 1.39 (95%CI 1.36-1.41) in Model 3 (Table 2). The IRR increased with multimorbidity severity, with an IRR of 1.73 (95%CI 1.68-1.78) among individuals with ≥5 NCDs. The subHR for the individuals with multimorbidity in Model 3 was 1.36 (95%CI 1.34-1.39) and increased with multimorbidity severity with a subHR of 1.62 (95%CI 1.57-1.67) among individuals with ≥5 NCDs. Fig. 1 presents the CIF for VTE and KM survival curves illustrating VTE-free survival time, stratified by multimorbidity severity. Supplementary Fig. 5 presents a forest plot displaying hazard ratios for VTE stratified by multimorbidity severity.

**Table 2:** Subdistribution Hazard Ratios (subHR), incidence rates, and incidence rate ratios for multimorbidity for Landmark 2 based on 45 diseases.

Multimorbidity score	Person years, No.	Cases, No./persons at risk,/No	Incidence rate, cases/1000 person-years	Incidence rate ratio (95%CI)			subHR (95%CI)		
				Model 1	Model 2	Model 3	Model 1	Model 2	Model 3
Score <2	23415101	24316/5987093	1.04 (1.03-1.05)	1[Reference]	1[Reference]	1[Reference]	1[Reference]	1[Reference]	1[Reference]
Score ≥2	8317341	30896/2183236	3.71 (3.67-3.76)	3.58 (3.52-3.64)	1.40 (1.37-1.42)	1.39 (1.36-1.41)	3.50 (3.44-3.56)	1.37 (1.35-1.40)	1.36 (1.34-1.39)
Score 0	16931766	14720/4338142	0.87 (0.86-0.88)	1[Reference]	1[Reference]	1[Reference]	1[Reference]	1[Reference]	1[Reference]
Score 1	6483335	9596/1648951	1.48 (1.45-1.51)	1.70 (1.66-1.75)	1.22 (1.19-1.25)	1.19 (1.16-1.22)	1.72 (1.67-1.76)	1.24 (1.20-1.27)	1.21 (1.18-1.24)
Score 2	3476634	8422/891363	2.42 (2.37-2.47)	2.79 (2.71-2.86)	1.36 (1.33-1.40)	1.34 (1.30-1.37)	2.79 (2.72-2.87)	1.39 (1.35-1.43)	1.36 (1.32-1.39)
Score 3	2010281	6912/522119	3.44 (3.36-3.52)	3.95 (3.84-4.07)	1.47 (1.43-1.51)	1.45 (1.41-1.49)	3.92 (3.81-4.04)	1.49 (1.44-1.53)	1.46 (1.41-1.50)
Score 4	1200082	5169/316792	4.31 (4.19-4.43)	4.95 (4.80-5.11)	1.52 (1.47-1.57)	1.50 (1.46-1.55)	4.84 (4.69-5.00)	1.52 (1.47-1.57)	1.49 (1.44-1.54)
Score ≥5	1630344	10392/452962	6.37 (6.25-6.50)	7.33 (7.15-7.52)	1.74 (1.69-1.79)	1.73 (1.68-1.78)	6.83 (6.66-7.01)	1.63 (1.58-1.68)	1.62 (1.57-1.67)

Model 1 is a crude model. Model 2 is adjusted for sex, year of birth, and educational attainment. Model 3 is adjusted for sex, year of birth, educational attainment, country of birth, VTE in sibling, Human immunodeficiency virus (HIV) disease, Chronic viral hepatitis B, Chronic viral hepatitis C, Other thrombophilia, Activated protein C resistance [factor V Leiden mutation], Deficiency antithrombin, Deficiency protein C, Deficiency protein S, Prothrombin gene mutation. Reference for multimorbidity was no or one disease (score = 0 or 1). Reference for multimorbidity severity (0-≥5) was no disease (score = 0).CI=Confidence interval.

### 3.4 Multimorbidity and incident VTE stratified for sex and year of birth

Among individuals with multimorbidity, we observed a sex difference in subHR with a subHR of 1.30 (95%CI 1.27-1.33) for males and 1.45 (95%CI 1.41-1.49) for females in Model 3. The subHR increased with multimorbidity severity and was 1.48 (95%CI 1.43-1.54) for males and 1.78 (95%CI 1.71-1.86) for females with multimorbidity severity of ≥5 (Supplementary Tables 7–8). The multimorbidity-by-sex interaction term was statistically significant (p for interaction <0.001) (Supplementary Table 9).

When stratified for birth quartiles (Q) the subHR was 1.28 (95%CI 1.26-1.31) for Q1(1908-1958), 1.61 (95%CI 1.54-1.68) for Q2(1959-1976), 1.77 (95%CI 1.65-1.90) for Q3 (1977-1996) and 1.21 (95%CI 0.91-1.61) for Q4 (1997-2014) (Supplementary Table 10). The subHR increased with increasing multimorbidity severity for all quartiles (Supplementary Table 11).

### 3.5 Multimorbidity and incident VTE by country of birth

A difference in subHR among individuals with multimorbidity was observed in relation to country of birth. Born in Sweden subHR of 1.33 (95%CI 1.30-1.36), other Nordic countries 1.53 (95%CI 1.38-1.68), other European countries 1.54 (95%CI 1.38-1.73) and 1.64 (95%CI 1.52-1.78) born outside Europe (Supplementary Table 12). The difference increased with increasing multimorbidity severity, subHR of 1.56 (95%CI 1.51-1.60) in Swedish-born individuals with multimorbidity severity of ≥5 and subHR of 2.20 (95%CI 1.95-2.48) born outside Europe (Supplementary Table 13). The multimorbidity-by-country of birth interaction term was statistically significant (p for interaction <0.001) (Supplementary Table 14).

### 3.6 Multimorbidity disease clusters and incident VTE

Seven of nine disease clusters were associated with higher subHR for multimorbidity (Table 3, Supplementary Fig. 6). In F1, cardiometabolic disease cluster, the subHR in Model 3 was 1.14 (95%CI 1.12-1.17), in F2, psychiatric disease cluster, the subHR in Model 3 was 1.39 (95%CI 1.34-1.44), in F3, digestive system-disease cluster, the subHR in Model 3 was 1.80 (95%CI 1.39-2.32) (Table 3). The subHR showed an increase with increasing multimorbidity severity (Supplementary Table 15).

**Table 3:** Subdistribution Hazard Ratios (subHR), incidence rates, and incidence rate ratios with 95% confidence intervals (CI) for venous thromboembolism (VTE) according to multimorbidity for Landmark 2 based on 45 diseases and stratified for nine different multimorbidity disease clusters (F1-F9).

Multimorbidity score	Person years, No.	Cases, No./persons at risk,/No	Incidence rate, cases/1000 person-years	Incidence rate ratio (95%CI)			subHR (95%CI)		
				Model 1	Model 2	Model 3	Model 1	Model 2	Model 3
F1 Score <2	29821911	43770/7644103	1.47 (1.45-1.48)	1[Reference]	1[Reference]	1[Reference]	1[Reference]	1[Reference]	1[Reference]
F1 Score ≥2	1910531	11441/526226	5.99 (5.88-6.10)	4.08 (4.00-4.16)	1.20 (1.17-1.23)	1.20 (1.18-1.23)	3.83 (3.75-3.91)	1.14 (1.11-1.16)	1.14 (1.12-1.17)
F2 Score <2	30374897	51365/7818523	1.69 (1.68-1.71)	1[Reference]	1[Reference]	1[Reference]	1[Reference]	1[Reference]	1[Reference]
F2 Score ≥2	13557545	3846/351806	2.83 (2.74-2.92)	1.68 (1.62-1.73)	1.44 (1.39-1.49)	1.42 (1.38-1.47)	1.67 (1.61-1.72)	1.40 (1.36-1.45)	1.39 (1.34-1.44)
F3 Score <2	31723204	55151/8167747	1.74 (1.72-1.75)	1[Reference]	1[Reference]	1[Reference]	1[Reference]	1[Reference]	1[Reference]
F3 Score ≥2	9238	60/2582	6.49 (5.04-8.37)	3.74 (2.90-4.82)	2.07 (1.61-2.67)	1.96 (1.52-2.52)	3.47 (2.70-4.48)	1.91 (1.48-2.46)	1.80 (1.39-2.32)
F4 Score <2	31482988	53136/8096467	1.69 (1.67-1.70)	1[Reference]	1[Reference]	1[Reference]	1[Reference]	1[Reference]	1[Reference]
F4 Score ≥2	249454	2075/73862	8.32 (7.97-8.68)	4.93 (4.72-5.15)	1.24 (1.18-1.29)	1.25 (1.20-1.31)	4.33 (4.15-4.53)	1.10 (1.05-1.15)	1.12 (1.07-1.17)
F5 Score <2	31572099	54085/8125959	1.71 (1.70-1.73)	1[Reference]	1[Reference]	1[Reference]	1[Reference]	1[Reference]	1[Reference]
F5 Score ≥2	160343	1126/44370	7.02 (6.62-7.44)	4.10 (3.86-4.35)	1.34 (1.26-1.42)	1.33 (1.26-1.42)	3.85 (3.63-4.08)	1.28 (1.21-1.36)	1.28 (1.20-1.36)
F6 Score <2	30641557	48824/7879441	1.59 (1.58-1.61)	1[Reference]	1[Reference]	1[Reference]	1[Reference]	1[Reference]	1[Reference]
F6 Score ≥2	1090885	6387/290888	5.85 (5.71-6.00)	3.67 (3.58-3.77)	1.22 (1.19-1.26)	1.23 (1.19-1.26)	3.57 (3.48-3.67)	1.23 (1.20-1.27)	1.24 (1.20-1.27)
F7 Score <2	31721579	55148/8167276	1.74 (1.72-1.75)	1[Reference]	1[Reference]	1[Reference]	1[Reference]	1[Reference]	1[Reference]
F7 Score ≥2	10862	63/3053	5.80 (4.53-7.42)	3.34 (2.61-4.27)	1.07 (0.83-1.36)	1.07 (0.84-1.38)	3.08 (2.41-3.94)	0.99 (0.77-1.27)	1.00 (0.78-1.29)
F8 Score <2	30637558	51883/7883829	1.69 (1.68-1.71)	1[Reference]	1[Reference]	1[Reference]	1[Reference]	1[Reference]	1[Reference]
F8 Score ≥2	1094883	3328/286500	3.04 (2.94-3.14)	1.79 (1.73-1.86)	1.38 (1.34-1.43)	1.39 (1.34-1.44)	1.77 (1.71-1.83)	1.31 (1.27-1.36)	1.32 (1.27-1.36)
F9 Score <2	31731932	55206/8170156	1.74 (1.73-1.75)	1[Reference]	1[Reference]	1[Reference]	1[Reference]	1[Reference]	1[Reference]
F9 Score ≥2	510	5/173	9.81 (4.08-23.58)	5.64 (2.35-13.55)	1.69 (0.70-4.06)	1.65 (0.69-3.97)	4.34 (1.80-10.44)	1.34 (0.56-3.20)	1.29 (0.54-3.13)

Model 1 is a crude model (univariable). Model 2 is an adjusted model (multivariable), with adjustments for year of birth, sex, and educational attainment. Model 3 is adjusted for sex, year of birth, educational attainment, country of birth, VTE in sibling, Human immunodeficiency virus (HIV) disease, Chronic viral hepatitis B, Chronic viral hepatitis C, Other thrombophilia, Activated protein C resistance [factor V Leiden mutation], Deficiency antithrombin, Deficiency protein C, Deficiency protein S, Prothrombin gene mutation.

F1 = hypertension, heart failure, coronary heart disease, diabetes, obesity, atrial fibrillation, gout, atherosclerosis, and renal disease; F2 = affective disorders, anxiety, psychoactive substance misuse, alcohol misuse disorders, anorexia or bulimia, and schizophrenia disorders; F3 = inflammatory bowel disease, liver disease, pancreatic disease, and ulcers; F4 = epilepsy, blindness and poor vision, cerebrovascular disease, cancer, and impaired or hearing loss; F5 = connective tissue disease, osteoporosis, thyroid disorders, and psoriasis; F6 = prostate disease, arthrosis, painful back condition, diverticular disease of intestine, and chronic sinusitis; F7 = bronchiectasis, Parkinson's disease, glaucoma, learning disability, and irritable bowel syndrome; F8 = asthma, dermatitis and eczema, constipation, chronic obstructive pulmonary disease, and migraine; and F9 = multiple sclerosis and dementia. Reference with no or one disease (score = 0 or 1).

### 3.7 Sensitivity analysis

Sensitivity analysis stratified by relevant covariates revealed no substantial differences in the findings (Supplementary Tables 16–24). Adjustment for varicose veins in a sensitivity analysis showed no substantial changes in the results (Supplementary Table 25). A sensitivity analysis including individuals with HRT, OC, DOACs, and Warfarin treatment at baseline showed no substantial differences in the results (Supplementary Table 26). The results for Landmark 1 are presented in Supplementary Tables 27–36 and Supplementary Figs. 7–9.

## 4 Discussion

Our study demonstrates that primary care-diagnosed multimorbidity, along with its severity, serves as a dose-graded risk factor for incident VTE. This risk persists after adjusting for the included acquired and inherited risk factors. We found that multimorbidity was more important regarding VTE risk among females than males and among immigrants than Swedish-born individuals. Additionally, our findings indicate an increased risk of VTE in seven out of the nine previously identified disease clusters, which is consistent with earlier research [19]. The substantial difference in subHR between Model 1 and Models 2 and 3 is primarily attributed to the adjustment for age (Supplementary Tables 37–38). The subHR for VTE was highest in individuals with multimorbidity born in 1977-1996, followed by those born in 1959-1976. Both groups had significantly higher risks than the oldest born in 1908–1958, and the youngest born in 1997–2014. This age-related variation in risk may be in part explained by the fact that age itself is a strong, independent risk factor for VTE [10]. Additionally, older individuals may have diseases not captured by the multimorbidity index used in this study. Multimorbidity may exert a greater relative impact among middle-aged individuals, for whom accumulating chronic diseases could contribute more substantially to VTE risk. The lower risk observed among the youngest group likely reflect the overall lower incidence of VTE at younger ages and the lower prevalence of multimorbidity in this population. Further research is needed to explore these age-related patterns and the potential limitations of the multimorbidity measure. These results highlight the role that multimorbidity plays in increasing the likelihood of VTE, even after adjusting for the included genetic and acquired risk factors. Our observation of higher impact of multimorbidity in females than males has not been shown previously in a primary-care setting, suggesting that sex-related biological or social factors may be important. The higher impact of multimorbidity in immigrants regarding VTE risk is a novel finding and may reflect differences in genetic factors, inequalities in socioeconomic status, or healthcare access and requires further research. The increased risk in seven of nine previously described disease clusters supports their validity as markers for increased VTE risk [19]. This consistency supports the relevance of these disease clusters and highlights their potential for identifying populations at elevated VTE risk.

### 4.1 Strengths and limitations

A major strength is the large and diverse cohort representing all ages and both Swedish-born and foreign-born individuals, enhancing generalizability compared with previous, more homogenous studies on multimorbidity and VTE [[16], [17], [18], [19]]. Another strength is the comprehensive adjustment for potential confounders, including sociodemographic, acquired and inherited. OC or HRT use were excluded, due to the complexity of modelling these time-varying exposures in the large cohort and due to the lack of formulation-specific information, leading to risk of exposure misclassification. We excluded individuals using DOACs, Warfarin, OC, HRT before baseline to reduce the risk of confounding and improve temporal associations. Another strength is the use of large Swedish registers with high coverage and reliability for VTE and the large sample size provides good statistical power, allowing for subgroup and sensitivity analyses [[20], [21], [22]]. A limitation is the lack of biological data and lifestyle-related variables in the national registers. To account for this to some extent, we included educational attainment as an adjustment for lifestyle factors [29,30]. Another limitation is that multimorbidity and other covariates were assessed at baseline and not updated over time. This static approach may not fully capture changes in health status during follow-up. However, in a sensitivity analysis, we used a landmark design to control for this. Another limitation is the exclusion of patients using OC or HRT, which might affect the generalizability for this group of patients. Risk stratification may also be challenging when the incidence is below 1 %. During the study period, the Swedish primary-care Act on System of Choice in Public Sector reform was gradually implemented, leading to an increase in private healthcare providers [31]. The use of two landmark time points strengthens and validates any potential impact that changes in diagnostic practices may have had during the study period.

Our findings indicate that multimorbidity could play a role in VTE risk assessment in primary care. Current models may underestimate VTE risk in patients with multiple chronic conditions, suggesting a need for future research to investigate whether multimorbidity should be included in prediction rules or diagnostic tools such as a modified Wells score. Future research should investigate the biological and social mechanisms linking multimorbidity and disease clusters to VTE. Understanding how multimorbidity influences thrombotic risk, including genetic, inflammatory, and metabolic factors, could offer valuable insights [[32], [33], [34]]. Another research question for future studies is how multimorbidity or multiple comorbidities affects the risk for recurrent VTE in patients with VTE. We have previously shown that Charlson Comorbidity Index is a risk factor for mortality in VTE patients [35].

## 5 Conclusion

Primary care-diagnosed multimorbidity, particularly its severity, is a significant and dose-dependent risk factor for incident venous thromboembolism (VTE). The risk remains after adjustment for genetic and acquired factors, supporting an independent effect of multimorbidity to thrombotic risk. Our findings also support the relevance of disease clusters in predicting VTE risk. With increasing multimorbidity, further research is needed on mechanisms linking specific disease clusters to VTE. These findings underscore the need for further research and for integrated care and multimorbidity-sensitive risk models in VTE prevention.

## CRediT authorship contribution statement

**Jonatan Ahrén:** Conceptualization, Data curation, Formal analysis, Investigation, Methodology, Visualization, Writing – original draft, Writing – review & editing. **MirNabi Pirouzifard:** Conceptualization, Methodology, Supervision, Validation, Writing – review & editing, Formal analysis. **Björn Holmquist:** Conceptualization, Methodology, Supervision, Validation, Writing – review & editing. **Jan Sundquist:** Conceptualization, Methodology, Project administration, Validation, Writing – review & editing, Data curation. **Kristina Sundquist:** Conceptualization, Funding acquisition, Methodology, Resources, Writing – review & editing, Project administration, Software, Supervision, Validation. **Bengt Zöller:** Conceptualization, Data curation, Formal analysis, Funding acquisition, Investigation, Methodology, Project administration, Resources, Software, Supervision, Validation, Writing – review & editing.

## Data sharing statement

The data used in this study cannot be shared publicly as it originates from third-party registries maintained by Swedish authorities. This restriction is in place to ensure the confidentiality and privacy of individuals, which could be compromised by unrestricted access. However, researchers may request access to the data under certain conditions through the National Board of Health and Welfare and Statistics Sweden. To do so, applicants must submit a request, referencing this study and specifying the variables required. Requests should be directed to: National Board of Health and Welfare: https://www.socialstyrelsen.se/en/statistics-and-data/statistics/statisticaldatabase/and Statistics Sweden: https://www.scb.se/en/About-us/contact-us/

These authorities will evaluate the request and manage the approval process accordingly.

## Funding

ALF-funding from Region Skåne and The 10.13039/501100004359Swedish Research Council to Dr. Zöller (2020-01824) and Dr. K. Sundquist as well as the Swedish Heart Lung Foundation to Dr. K. Sundquist.

## Declaration of competing interest

The authors declare that they have no known competing financial interests or personal relationships that could have appeared to influence the work reported in this paper.
