# Supplementary material for: Primary care diagnosed multimorbidity and risk of venous thromboembolism in Sweden
Source: Int J Cardiol Cardiovasc Risk Prev. 2026 Apr 5;29:200631. doi: 10.1016/j.ijcrp.2026.200631 (PMC13091224; doi:10.1016/j.ijcrp.2026.200631)
Supplement: Multimedia component 1 [file mmc1.docx]

**Supplementary Appendix: Primary care diagnosed multimorbidity and risk of venous thromboembolism in Sweden**

Authors: Ahrén J, Pirouzifard M, Holmquist B, Sundquist J, Sundquist K, Zöller B

| **Supplementary Table 1.** Number of visits registered according to county in the Swedish Primary Healthcare data. | | | |
| --- | --- | --- | --- |
| **County**  **(abbreviation)** | **County** | **Number of visits to Primary Healthcare** | **Percentage of total number of visits to Swedish Primary Healthcare** |
| LB | Region Blekinge | 5042364 | 1.31 |
| LD | Region Dalarna | 5318139 | 1.38 |
| LIV | Region Värmland | 7926760 | 2.06 |
| LK | Region Kalmar Län | 4007519 | 1.04 |
| LS | Region Sörmland | 8882992 | 2.31 |
| LTV | Region Västmanland | 1868740 | 0.49 |
| LUL | Region Uppsala | 3450565 | 0.90 |
| LVN | Region Västernorrland | 9435315 | 2.46 |
| NLL | Region Norrbotten | 8931876 | 2.33 |
| RG | Region Gävleborg | 4972009 | 1.29 |
| RGT | Region Gotland | 673539 | 0.18 |
| RH | Region Halland | 2604001 | 0.68 |
| RJ | Region Jönköping län | 11151592 | 2.90 |
| RK | Region Kronoberg | 3536345 | 0.92 |
| RS | Region Skåne | 37105621 | 9.66 |
| RV | Region Västerbotten | 6656068 | 1.73 |
| RÖ | Region Östergötland | 21919204 | 5.71 |
| RÖL | Region Örebro | 3176065 | 0.83 |
| SLL | Region Stockholm | 138498912 | 36.07 |
| VGR | Region Västra Götaland | 98852907 | 25.74 |

| **Supplementary Table 2.** Number of visits registered according to year in the Swedish Primary Health Care data. | | |
| --- | --- | --- |
| **Year** | **Visits** | **%** |
| 1989 | 145 | 0.00 |
| 1990 | 13434 | 0.00 |
| 1991 | 17667 | 0.00 |
| 1992 | 33255 | 0.01 |
| 1993 | 150057 | 0.04 |
| 1994 | 377575 | 0.10 |
| 1995 | 548380 | 0.14 |
| 1996 | 733333 | 0.19 |
| 1997 | 790776 | 0.21 |
| 1998 | 818703 | 0.21 |
| 1999 | 779817 | 0.20 |
| 2000 | 1365136 | 0.36 |
| 2001 | 2000943 | 0.52 |
| 2002 | 1983042 | 0.52 |
| 2003 | 5471556 | 1.42 |
| 2004 | 7631152 | 1.99 |
| 2005 | 8752938 | 2.28 |
| 2006 | 11510451 | 3.00 |
| 2007 | 12720048 | 3.31 |
| 2008 | 14532877 | 3.78 |
| 2009 | 17108448 | 4.46 |
| 2010 | 20496614 | 5.34 |
| 2011 | 24375137 | 6.35 |
| 2012 | 28619605 | 7.45 |
| 2013 | 32847908 | 8.55 |
| 2014 | 33361850 | 8.69 |
| 2015 | 38500602 | 10.03 |
| 2016 | 36080955 | 9.40 |
| 2017 | 39096981 | 10.18 |
| 2018 | 43291117 | 11.27 |

| **Supplementary Table 3.** Diseases with ICD codes included in the multimorbidity score modified and adapted to Swedish ICD-10 codes. One point for each disease. The theoretical range of the score is 0-45 points. | | |
| --- | --- | --- |
|  | **Included disorders in the multimorbidity score** | **ICD-10** |
| 1 | Hypertension | I10-I15 |
| 2 | Affective disorders | F30-F39 |
| 3 | Painful back condition | M50-54 |
| 4 | Asthma | J45-J46 |
| 5 | CHD | I20-I25 |
| 6 | Ulcer disease | K221, K25-K28 |
| 7 | Diabetes | E10-E14 |
| 8 | Thyroid disorders | E00-E07 |
| 9 | Connective tissue disease | M05, M06, M08, M09, M30-M36, D86 |
| 10 | Hearing loss (or impaired) | H90-H91 |
| 11 | Chronic obstructive pulmonary disease (COPD) | J41-J44 |
| 12 | Anxiety | F40-F48 |
| 13 | Irritable bowel syndrome (IBS) | K58 |
| 14 | Cancer | C00-C99 |
| 15 | Alcohol misuse disorders | F10 |
| 16 | Psychoactive substance misuse | F11-F14, F16, F18, F19 |
| 17 | Constipation | K590 |
| 18 | Cerebrovascular disease | I60-I69 |
| 19 | Renal disease | N00-N19, Q61 |
| 20 | Diverticular disease of intestine | K57 |
| 21 | Atrial fibrillation | I48 |
| 22 | Atherosclerosis | I70-I74, I77 |
| 23 | Heart failure | I50, I110, I130, I132 |
| 24 | Prostate disease | N40-N42 |
| 25 | Glaucoma | H40, H42 |
| 26 | Epilepsy | G40, G41 |
| 27 | Dementia | F00-F03 |
| 28 | Schizophrenia disorders | F20, F21 |
| 29 | Psoriasis | L40 |
| 30 | Dermatitis or Eczema | L20-L30 |
| 31 | Inflammatory bowel disease (IBD) | K50, K51 |
| 32 | Migraine | G43, G440, G441 |
| 33 | Blindness & low vision | H53-H54 |
| 34 | Chronic sinusitis | J32 |
| 35 | Learning disability | F81 |
| 36 | Anorexia or bulimia | F50 |
| 37 | Bronchiectasis | J47 |
| 38 | Parkinson’s disease | G20, G21, G22 |
| 39 | Multiple sclerosis | G35 |
| 40 | Liver disease | K70-K77 |
| 41 | Gout | M10 |
| 42 | Arthrosis | M15-M19 |
| 43 | Osteoporosis | M80-M82 |
| 44 | Obesity | E65, E66 |
| 45 | Pancreas diseases | K85, K86 |

| Supplementary Table 4 Nine previously defined disease clusters F1-F9 | |
| --- | --- |
| F1 | Hypertension, heart failure, coronary heart disease, diabetes, obesity, atrial fibrillation, gout, atherosclerosis, and renal disease |
| F2 | Affective disorders, anxiety, psychoactive substance misuse, alcohol misuse disorders, anorexia or bulimia, and schizophrenia disorders |
| F3 | Inflammatory bowel disease, liver disease, pancreatic disease, and ulcers |
| F4 | Epilepsy, blindness and poor vision, cerebrovascular disease, cancer, and impaired or hearing loss |
| F5 | Connective tissue disease, osteoporosis, thyroid disorders, and psoriasis |
| F6 | Prostate disease, arthrosis, painful back condition, diverticular disease of intestine, and chronic sinusitis |
| F7 | Bronchiectasis, Parkinson’s disease, glaucoma, learning disability, and irritable bowel syndrome |
| F8 | Asthma, dermatitis and eczema, constipation, chronic obstructive pulmonary disease, and migraine |
| F9 | Multiple sclerosis and dementia |

| **Supplementary Table 5.** Descriptive findings for study participants in Landmark 1 before exclusion of Hormonal Replacement Therapy, Oral contraceptive, Direct Oral Anticoagulant and Warfarin. | | | | | | | | | |
| --- | --- | --- | --- | --- | --- | --- | --- | --- | --- |
| **Multimorbidity score** | | | | | | | | | |
|  | All | 0 | 1 | 2 | 3 | 4 | ≥ 5 | ≤ 1 | ≥ 2 |
| Unique individuals, n (%) | 9577707  (100) | 6587781  (68.78) | 1496264  (15.62) | 699172  (7.3) | 371121  (3.87) | 202786  (2.12) | 220377  (2.3) | 8084045  (84.4) | 1493662  (15.6) |
| Sex, Female,  n (%) | 4769430  (49.8) | 3104831  (65.1) | 789714  (16.56) | 399513  (8.38) | 220497  (4.62) | 121874  (2.56) | 133001  (2.79) | 3894545  (81.66) | 874545  (18.34) |
| Education (≥12 years), n (%) | 3827536  (39.96) | 2839477  (43.10) | 592633  (39.61) | 219924  (31.45) | 94098  (25.36) | 43425  (21.41) | 37979  (17.23) | 3432110  (42.46) | 395426  (26.47) |
| Age at end of study,  Median (IQR)  Range (min-max) | 45  (28-66)  (0.08-111) | 40  (25-59)  (0.08-111) | 52  (34-68)  (1-109) | 64  (47-77)  (1.8-109) | 71  (56-82)  (3-108) | 75  (63-85)  (6-109) | 80  (70-88)  (6-108) | 42  (26-61)  (0.08-111) | 70  (54-82)  (1.8-109) |
| VTE,  n (%) | 152667  (1.59) | 73582  (1.12) | 27162  (1.82) | 19169  (2.74) | 13344  (3.60) | 8437  (4.16) | 10973  (5.00) | 100744  (1.25) | 51924  (3.48) |
| VTE= Venous thromboembolism, IQR=Interquartile Range | | | | | | | | | |

| **Supplementary Table 6.** Descriptive findings for study participants in Landmark 2 before exclusion of Hormonal Replacement Therapy, Oral contraceptive, Direct Oral Anticoagulant and Warfarin. | | | | | | | | | |
| --- | --- | --- | --- | --- | --- | --- | --- | --- | --- |
| **Multimorbidity score** | | | | | | | | | |
|  | All | 0 | 1 | 2 | 3 | 4 | ≥ 5 | ≤ 1 | ≥ 2 |
| Unique individuals,  n (%) | 9525240  (100) | 4750806  (49.88) | 1914033  (20.09) | 1085515  (11.40) | 666182  (6.99) | 422506  (4.44) | 686198  (7.20) | 664839  (69.97) | 2860401  (30.03) |
| Sex, Female,  n (%) | 4736466  (49.73) | 2136610  (45.11) | 956803  (20.20) | 593874  (12.54) | 385605  (8.14) | 250785  (5.29) | 412789  (8.72) | 3093413  (65.31) | 1643053  (34.69) |
| Education (≥12 years),  n (%) | 4043631  (42.45) | 2264640  (47.67) | 865752  (45.23) | 420355  (38.72) | 218257  (32.76) | 120637  (28.55) | 153990  (22.44) | 3130392  (46.97) | 913239  (31.93) |
| Age at end of study,  Median (IQR)  Range (min-max) | 43  (25-63)  (0.1-111) | 32  (17-50)  (0.1-111) | 42  (25-59)  (1-109) | 52  (35-68)  (1-109) | 61  (44-74)  (1-109) | 67  (52-78)  (5-109) | 74  (62-84)  (2-109) | 34  (19-53)  (0.1-111) | 63  (45-76)  (1.5-109) |
| VTE  n (%) | 69575  (0.73) | 17066  (0.36) | 11342  (0.59) | 10196  (0.94) | 8761  (1.32) | 6836  (1.62) | 15374  (2.24) | 28408  (0.41) | 41167  (1.42) |
| VTE= Venous thromboembolism, IQR=Interquartile Range | | | | | | | | | |

| **Supplementary Table 7**. Subdistributional Hazard Ratios (subHR), incidence rates, and incidence-rate ratios with 95% confidence intervals (CI) for multimorbidity for Landmark 2 males based on 45 diseases. | | | | | | | | | |
| --- | --- | --- | --- | --- | --- | --- | --- | --- | --- |
| **Multimorbidity score** | **Person years, No.** | **Cases, No./persons at risk, /No** | **Incidence rate, cases/1000 person-years** | **Incidence rate ratio (95%CI)**  **Model 1 Model 2 Model 3** | | | **subHR (95%CI)**  **Model 1 Model 2 Model 3** | | |
| Score <2 | 13874328 | 15195/3546808 | 1.10 (1.08-1.11) | 1[Reference] | 1[Reference] | 1[Reference] | 1[Reference] | 1[Reference] | 1[Reference] |
| Score ≥2 | 4271461 | 16743/1123970 | 3.92 (3.86-3.98) | 3.58 (3.50-3.66) | 1.33 (1.30-1.37) | 1.33 (1.30-1.36) | 3.49 (3.42-3.57) | 1.31 (1.28-1.34) | 1.30 (1.27-1.33) |
| Score 0 | 10150301 | 9348/2599357 | 0.92 (0.90-0.94) | 1[Reference] | 1[Reference] | 1[Reference] | 1[Reference] | 1[Reference] | 1[Reference] |
| Score 1 | 3724026 | 5847/947451 | 1.57 (1.53-1.61) | 1.79 (1.76-1.82) | 1.22 (1.18-1.26) | 1.20 (1.16-1.24) | 1.72 (1.66-1.78) | 1.24 (1.20-1.28) | 1.21 (1.17-1.25) |
| Score 2 | 1864479 | 4808/478527 | 2.58 (2.51-2.65) | 2.80 (2.70-2.90) | 1.34 (1.29-1.39) | 1.31 (1.27-1.36) | 2.80 (2.71-2.90) | 1.36 (1.31-1.41) | 1.33 (1.28-1.38) |
| Score 3 | 1019587 | 3761/265500 | 3.69 (3.57-3.81) | 4.01 (3.86-4.16) | 1.41 (1.35-1.46) | 1.38 (1.33-1.44) | 3.96 (2.71-2.90) | 1.42 (1.36-1.47) | 1.39 (1.33-1.45) |
| Score 4 | 590922 | 2816/1256789 | 4.77 (4.59-4.94) | 5.17 (4.96-5.40) | 1.47 (1.41-1.54) | 1.46 (1.39-1.52) | 5.03 (4.82-5.25) | 1.46 (1.40-1.53) | 1.44 (1.38-1.50) |
| Score ≥5 | 796472 | 5349/223154 | 6.72 (6.54-6.90) | 7.29 (7.05-7.54) | 1.61 (1.55-1.67) | 1.60 (1.54-1.66) | 6.74 (6.52-6.97) | 1.50 (1.43-1.55) | 1.48 (1.43-1.54) |
| Model 1 is a crude model. Model 2 is adjusted for year of birth, and educational attainment. Model 3 is adjusted for year of birth, educational attainment, country of birth, VTE in sibling, Human immunodeficiency virus (HIV) disease, Chronic viral hepatitis B, Chronic viral hepatitis C, Other thrombophilia, Activated protein C resistance [factor V Leiden mutation], Deficiency antithrombin, Deficiency protein C, Deficiency protein S, Prothrombin gene mutation Reference for multimorbidity was no or one disease (score=0 or 1). Reference for multimorbidity severity (0-≥5) was no disease (score=0). | | | | | | | | | |

| **Supplementary Table 8**. Subdistributional Hazard Ratios (subHR), incidence rates, and incidence rate ratios with 95% confidence intervals (CI) for multimorbidity for Landmark 2 females based on 45 diseases. | | | | | | | | | |
| --- | --- | --- | --- | --- | --- | --- | --- | --- | --- |
| **Multimorbidity score** | **Person years, No.** | **Cases, No./persons at risk, /No** | **Incidence rate, cases/1000 person-years** | **Incidence rate ratio (95%CI)**  **Model 1 Model 2 Model 3** | | | **subHR (95%CI)**  **Model 1 Model 2 Model 3** | | |
| Score <2 | 9540733 | 9121/2440285 | 0.96 (0.94-0.98) | 1[Reference] | 1[Reference] | 1[Reference] | 1[Reference] | 1[Reference] | 1[Reference] |
| Score ≥2 | 4045879 | 14161/1059266 | 3.50 (3.44-3.56) | 3.66 (2.57-3.76) | 1.48 (1.44-1.52) | 1.47 (1.43-1.51) | 3.59 (3.50-3.69) | 1.46 (1.42-1.50) | 1.45 (1.41-1.49) |
| Score 0 | 6781464 | 5372/1738785 | 0.79 (0.77-0.81) | 1[Reference] | 1[Reference] | 1[Reference] | 1[Reference] | 1[Reference] | 1[Reference] |
| Score 1 | 2759309 | 3749/701500 | 1.36 (1.32-1.40) | 1.72 (1.65-1.79) | 1.21 (1.16-1.26) | 1.18 (1.14-1.24) | 1.73 (1.66-1.81) | 1.23 (1.18-1.28) | 1.20 (1.15-1.25) |
| Score 2 | 1612155 | 3614/412836 | 2.24 (2.17-2.32) | 2.83 (2.71-2.95) | 1.40 (1.34-1.46) | 1.37 (1.31-1.43) | 2.84 (2.72-2.96) | 1.42 (1.36-1.49) | 1.39 (1.33-1.45) |
| Score 3 | 990693 | 3151/256619 | 3.18 (3.07-3.29) | 4.02 (3.84-4.20) | 1.55 (1.49-1.63) | 1.53 (1.46-1.60) | 3.99 (3.82-4.17) | 1.57 (1.50-1.65) | 1.54 (1.47-1.62) |
| Score 4 | 609159 | 2353/160003 | 3.86 (3.71-4.02) | 4.88 (4.65-5.12) | 1.58 (1.51-1.66) | 1.56 (1.49-1.64) | 4.79 (4.56-5.03) | 1.58 (1.50-1.67) | 1.56 (1.48-1.64) |
| Score ≥5 | 833872 | 5043/229808 | 6.05 (5.88-6.22) | 7.63 (7.45-7.93) | 1.90 (1.83-1.98) | 1.89 (1.82-1.97) | 7.18 (6.90-7.46) | 1.80 (1.72-1.88) | 1.78 (1.71-1.86) |
| Model 1 is a crude model. Model 2 is adjusted for year of birth, and educational attainment. Model 3 is adjusted for year of birth, educational attainment, country of birth, VTE in sibling, Human immunodeficiency virus (HIV) disease, Chronic viral hepatitis B, Chronic viral hepatitis C, Other thrombophilia, Activated protein C resistance [factor V Leiden mutation], Deficiency antithrombin, Deficiency protein C, Deficiency protein S, Prothrombin gene mutation. Reference for multimorbidity was no or one disease (score=0 or 1). Reference for multimorbidity severity (0-≥5) was no disease (score=0). | | | | | | | | | |

| **Supplementary Table 9.**  **Multimorbidity-by-sex interaction of Venous Thromboembolism, including interaction terms Landmark 2.** | | | | |
| --- | --- | --- | --- | --- |
|  | subHR(95%CI) | p-value |  |  |
| Multimorbidity | 1.34 (1.31-1.37) | <0.001 |  |  |
| Sex, Female, | 0.84 (0.82-0.87) | <0.001 |  |  |
| Interaction: Multimorbidity x Female | 1.06 (1.02-1.10) | <0.001 |  |  |
| Multimorbidity | Male subHR (95%CI) | Female subHR (95% CI) | Interaction term subHR (95% CI) | p-value |
| 1 | 1.25 (1.21–1.29) | 1.30 (1.26–1.34) | 1.05 (0.99–1.12) | 0.329 |
| 2 | 1.38 (1.34–1.43) | 1.45 (1.40–1.50) | 1.01 (0.95–1.06) | 0.852 |
| 3 | 1.46 (1.41–1.53) | 1.55 (1.49–1.61) | 1.05 (0.99–1.12) | 0.102 |
| 4 | 1.51 (1.45–1.58) | 1.62 (1.55–1.70) | 1.01 (0.95–1.08) | 0.684 |
| ≥5 | 1.55 (1.50–1.61) | 1.78 (1.71–1.86) | 1.11 (1.05–1.17) | <0.001 |
| Model adjusted for sex, year of birth, educational attainment, country of birth, subHR=Subdistributional Hazard Ratios, CI=Confidence interval | | | | |

| **Supplementary Table 10.** Landmark 2 stratified for birth year quartiles (Q1-Q4). Subdistributional Hazard Ratios (subHR), incidence rates, and incidence rate ratios with 95% confidence intervals (CI), for venous thromboembolism (VTE) according to multimorbidity score ≥2 and birth year quartiles 1-4 (Q1-Q4) for Landmark 2. | | | | | | | | | | |
| --- | --- | --- | --- | --- | --- | --- | --- | --- | --- | --- |
| **Multimorbidity score** | **Person years, No.** | **Cases, No./persons at risk, /No** | **Incidence rate, cases/1000 person-years** | **Incidence rate ratio (95%CI)**  **Model 1 Model 2 Model 3** | | | **subHR (95%CI)**  **Model 1 Model 2 Model 3** | | |  |
| Q1 Score <2 | 3581385 | 14381/931682 | 4.01 (3.95-4.08) | 1[Reference] | 1[Reference] | 1[Reference] | 1[Reference] | 1[Reference] | 1[Reference] |  |
| Q1 Score ≥2 | 4052652 | 24884/1103980 | 6.14 (6.06-6.22) | 1.53 (1.50-1.56) | 1.31 (1.28-1.33) | 1.31 (1.28-1.34) | 1.47 (1.44-1.50) | 1.28 (1.25-1.31) | 1.28 (1.26-1.31) |  |
| Q2 Score <2 | 5490562 | 6684/1395556 | 1.22 (1.19-1.25) | 1[Reference] | 1[Reference] | 1[Reference] | 1[Reference] | 1[Reference] | 1[Reference] |  |
| Q2 Score ≥2 | 2345332 | 4639/594210 | 1.98 (1.92-2.04) | 1.62 (1.56-1.69) | 1.61 (1.55-1.67) | 1.60 (1.54-1.67) | 1.63 (1.57-1.69) | 1.61 (1.55-1.67) | 1.61 (1.54-1.68) |  |
| Q3 Score <2 | 6755947 | 2841/1741003 | 0.42 (0.41-0.44) | 1[Reference] | 1[Reference] | 1[Reference] | 1[Reference] | 1[Reference] | 1[Reference] |  |
| Q3 Score ≥2 | 1356915 | 1316/343715 | 0.97 (0.92-1.02) | 2.31 (2.16-2.46) | 1.99 (1.86-2.12) | 1.76 (1.64-1.88) | 2.35 (2.20-2.51) | 2.03 (1.90-2.17) | 1.77 (1.65-1.90) |  |
| Q4 Score <2 | 7585407 | 410/1918852 | 0.054 (0.049-0.060) | 1[Reference] | 1[Reference] | 1[Reference] | 1[Reference] | 1[Reference] | 1[Reference] |  |
| Q4 Score ≥2 | 562441 | 56/141331 | 0.099 (0.088-0.13) | 1.84 (1.39-2.44) | 1.29 (0.98-1.71) | 1.21 (0.91-1.60) | 1.85 (1.40-2.45) | 1.30 (0.98-1.72) | 1.21 (0.91-1.61) |  |
| Model 1 is a crude model. Model 2 is adjusted for sex, year of birth, and educational attainment. Model 3 is adjusted for sex, year of birth, educational attainment, country of birth, VTE in sibling, Human immunodeficiency virus (HIV) disease, Chronic viral hepatitis B, Chronic viral hepatitis C, Other thrombophilia, Activated protein C resistance [factor V Leiden mutation], Deficiency antithrombin, Deficiency protein C, Deficiency protein S, Prothrombin gene mutation, Q1=1908-1958, Q2=1959-1976, Q3=1977-1996, Q4=1997-2014. Reference for multimorbidity was no or one disease (score=0 or 1). Reference for multimorbidity severity (0-≥5) was no disease (score=0). | | | | | | | | | | |

| **Supplementary Table 11.** Landmark 2 stratified for birth year quartiles (Q1-Q4). Incidence rates, incidence rate ratios, and Subdistributional Hazard Ratios (subHR) with 95% confidence intervals (CI) for venous thromboembolism (VTE) according to multimorbidity score (0 to ≥5) and birth year quartiles 1-4 (Q1-Q4). | | | | | | | | | | |
| --- | --- | --- | --- | --- | --- | --- | --- | --- | --- | --- |
| **Multimorbidity score** | **Person years, No.** | **Cases (n) /persons at risk (n)** | **Incidence rate, cases/1000 person-years** | **Incidence rate ratio (95%CI)**  **Model 1 Model 2 Model 3** | | | **subHR (95%CI)**  **Model 1 Model 2 Model 3** | | |  |
| **Q1=birth years 1908-1958 (n=2035662)** | | | | | | | | | | |
| Score 0 | 2170805 | 8384/564328 | 3.86 (3.78-3.95) | 1[Reference] | 1[Reference] | 1[Reference] | 1[Reference] | 1[Reference] | 1[Reference] |  |
| Score 1 | 1412380 | 5997/367354 | 4.24 (4.14-4.35) | 1.10 (1.06-1.14) | 1.10 (1.06-1.13) | 1.09 (1.05-1.13) | 1.10 (1.06-1.14) | 1.09 (1.06-1.13) | 1.09 (1.05-1.12) |  |
| Score 2 | 1227270 | 6063/322983 | 4.94 (4.82-5.07) | 1.28 (1.24-1.32) | 1.20 (1.16-1.24) | 1.19 (1.15-1.23) | 1.27 (1.23-1.31) | 1.20 (1.16-1.24) | 1.19 (1.15-1.23) |  |
| Score 3 | 964403 | 5363/257492 | 5.56 (5.41-5.71) | 1.44 (1.39-1.49) | 1.28 (1.24-1.32) | 1.28 (1.23-1.32) | 1.41 (1.36-1.46) | 1.27 (1.23-1.32) | 1.27 (1.23-1.32) |  |
| Score 4 | 694614 | 4250/288660 | 6.12 (5.94-6.31) | 1.58 (1.53-1.64) | 1.34 (1.29-1.39) | 1.34 (1.30-1.40) | 1.52 (1.47-1.58) | 1.32 (1.28-1.38) | 1.33 (1.28-1.38) |  |
| Score ≥5 | 1166365 | 9208/334845 | 7.89 (7.74-8.06) | 2.04 (1.98-2.11) | 1.59 (1.54-1.64) | 1.61 (1.56-1.66) | 1.86 (1.81-1.92) | 1.50 (1.46-1.55) | 1.52 (1.47-1.56) |  |
| **Q2=birth years 1959-1976(n=1989766)** | | | | | | | | | | |
| Score 0 | 3677030 | 4183/936990 | 1.14 (1.10-1.17) | 1[Reference] | 1[Reference] | 1[Reference] | 1[Reference] | 1[Reference] | 1[Reference] |  |
| Score 1 | 1813532 | 2501/458566 | 1.38 (1.33-1.43) | 1.21 (1.15-1.27) | 1.21 (1.15-1.27) | 1.19 (1.13-1.25) | 1.22 (1.16-1.28) | 1.22 (1.16-1.28) | 1.20 (1.14-1.26) |  |
| Score 2 | 1066399 | 1783/269683 | 1.67 (1.60-1.75) | 1.47 (1.39-1.55) | 1.47 (1.39-1.55) | 1.45 (1.37-1.53) | 1.48 (1.40-1.56) | 1.48 (1.40-1.56) | 1.46 (1.38-1.54) |  |
| Score 3 | 608089 | 1197/153943 | 1.97 (1.86-2.08) | 1.73 (1.62-1.85) | 1.72 (1.61-1.84) | 1.71 (1.61-1.83) | 1.74 (1.64-1.86) | 1.73 (1.62-1.85) | 1.72 (1.61-1.84) |  |
| Score 4 | 331591 | 700/84116 | 2.11 (1.96-2.27) | 1.86 (1.71-2.01) | 1.84 (1.69-1.99) | 1.84 (1.70-1.99) | 1.87 (1.72-2.02) | 1.84 (1.70-2.00) | 1.84 (1.70-2.00) |  |
| Score ≥5 | 339252 | 959/86468 | 2.83 (2.65-3.01) | 2.48 (2.32-2.67) | 2.41 (2.25-2.59) | 2.42 (2.36-2.60) | 2.49 (2.32-2.67) | 2.41 (2.25-2.59) | 2.42 (2.25-2.60) |  |
| **Q3=birth years 1977-1996 (n=2084718)** | | | | | | | | | | |
| Score 0 | 5061001 | 1875/1311017 | 0.37 (0.35-0.39) | 1[Reference] | 1[Reference] | 1[Reference] | 1[Reference] | 1[Reference] | 1[Reference] |  |
| Score 1 | 1694945 | 966/429986 | 0.57 (0.54-0.61) | 1.54 (1.42-1.66) | 1.48 (1.37-1.60) | 1.35 (1.25-1.46) | 1.57 (1.45-1.70) | 1.51 (1.40-1.64) | 1.37 (1.26-1.48) |  |
| Score 2 | 762279 | 547/192974 | 0.72 (0.66-0.78) | 1.94 (1.76-2.13) | 1.75 (1.59-1.92) | 1.56 (1.41-1.72) | 1.98 (1.80-2.18) | 1.79 (1.63-1.97) | 1.58 (1.43-1.74) |  |
| Score 3 | 338411 | 336/85723 | 0.99 (0.89-1.10) | 2.68 (2.39-3.01) | 2.27 (2.02-2.55) | 1.98 (1.76-2.23) | 2.74 (2.44-3.08) | 2.33 (2.07-2.62) | 2.00 (1.78-2.26) |  |
| Score 4 | 147820 | 213/37470 | 1.44 (1.26-1.65) | 3.89 (3.38-4.48) | 3.13 (2.71-3.61) | 2.64 (2.29-3.06) | 3.98 (3.46-4.59) | 3.21 (2.78-3.71) | 2.68 (2.31-3.10) |  |
| Score ≥5 | 108402 | 220/27548 | 2.03 (1.78-2.32) | 5.48 (4.76-6.30) | 4.16 (3.61-4.80) | 3.39 (2.93-3.91) | 5.61 (4.87-6.45) | 4.27 (3.70-4.93) | 3.42 (2.96-3.96) |  |
| **Q4= birth years 1997-2014 (n=2060183)** | | | | | | | | | | |
| Score 0 | 6022929 | 278/1525807 | 0.046 (0.041-0.052) | 1[Reference] | 1[Reference] | 1[Reference] | 1[Reference] | 1[Reference] | 1[Reference] |  |
| Score 1 | 1562478 | 132/393045 | 0.085 (0.071-0.10) | 1.83 (1.49-2.25) | 1.53 (1.24-1.88) | 1.42 (1.15-1.76) | 1.84 (1.50-2.27) | 1.53 (1.24-1.89) | 1.43 (1.15-1.76) |  |
| Score 2 | 420686 | 29/105723 | 0.069 (0.048-0.099) | 1.49 (1.02-2.19) | 1.07 (0.73-1.57) | 0.99 (0.68-1.46) | 1.51 (1.03-2.21) | 1.08 (0.73-1.58) | 0.99 (0.68-1.46) |  |
| Score 3 | 99376 | 16/24961 | 0.16 (0.099-0.26) | 3.49 (2.11-5.77) | 2.06 (1.24-3.42) | 1.91 (1.15-3.17) | 3.52 (2.13-5.82) | 2.07 (1.25-3.45) | 1.92 (1.15-3.19) |  |
| Score 4 | 26055 | 6/6546 | 0.023 (0.01-0.05) | 4.99 (2.22-11.20) | 2.66 (1.18-5.98) | 2.39 (1.06-5.38) | 5.03 (2.24-11.30) | 2.67 (1.19-6.01) | 2.39 (1.07-5.34) |  |
| Score ≥5 | 16325 | 5/4101 | 0.031 (0.012-0.074) | 6.64 (2.74-16.07) | 4.03 (1.66-9.78) | 3.53 (1.45-8.61) | 6.70 (2.77-16.21) | 4.06 (1.67-9.86) | 3.53 (1.42-8.77) |  |
| Abbreviations: Q=quartile. NA=not applicable due to few cases Model 1 is a crude model (univariate). Model 2 is an adjusted model (multivariate), with adjustments for sex, year of birth, and educational attainment. Model 3 is adjusted for sex, year of birth, educational attainment, country of birth, VTE in sibling, Human immunodeficiency virus (HIV) disease, Chronic viral hepatitis B, Chronic viral hepatitis C, Other thrombophilia, Activated protein C resistance [factor V Leiden mutation], Deficiency antithrombin, Deficiency protein C, Deficiency protein S, Prothrombin gene mutation. Reference = no diseases (score=0). | | | | | | | | | | |

| **Supplementary Table 12.** Landmark 2 stratified for Country of birth (CB). Subdistributional Hazard Ratios (subHR), incidence rates, and incidence rate ratios with 95% confidence intervals (CI), for venous thromboembolism (VTE) according to multimorbidity score ≥2 and Country of birth (CB 1-4) for Landmark 2. | | | | | | | | | |
| --- | --- | --- | --- | --- | --- | --- | --- | --- | --- |
| **Multimorbidity score** | **Person years, No.** | **Cases, No./persons at risk, /No** | **Incidence rate, cases/1000 person-years** | **Incidence rate ratio (95%CI)**  **Model 1 Model 2 Model 3** | | | **subHR (95%CI)**  **Model 1 Model 2 Model 3** | | |
| CB1 Score <2 | 18166443 | 21124/4599949 | 1.16 (1.15-1.18) | 1[Reference] | 1[Reference] | 1[Reference] | 1[Reference] | 1[Reference] | 1[Reference] |
| CB1 Score ≥2 | 6868484 | 27024/1805322 | 3.93 (3.89-3.98) | 3.38 (3.32-3.45) | 1.37 (1.35-1.40) | 1.36 (1.33-1.39) | 3.28 (3.22-3.34) | 1.34 (1.31-1.37) | 1.33 (1.30-1.36) |
| CB2 Score <2 | 404923 | 743/110983 | 1.83 (1.71-1.98) | 1[Reference] | 1[Reference] | 1[Reference] | 1[Reference] | 1[Reference] | 1[Reference] |
| CB2 Score ≥2 | 256156 | 1327/69685 | 5.18 (4.91-5.47) | 2.82 (2.58-3.09) | 1.54 (1.41-1.70) | 1.54 (1.40-1.69) | 2.86 (2.62-3.13) | 1.53 (1.39-1.69) | 1.53 (1.38-1.68) |
| CB3 Score <2 | 1049389 | 837/281658 | 0.80 (0.75-0.85) | 1[Reference] | 1[Reference] | 1[Reference] | 1[Reference] | 1[Reference] | 1[Reference] |
| CB3 Score ≥2 | 237584 | 922/63273 | 3.88 (3.64-4.14) | 4.87 (4.43-5.34) | 1.56 (1.40-1.73) | 1.54 (1.38-1.72) | 4.93 (4.49-5.42) | 1.55 (1.39-1.74) | 1.54 (1.38-1.73) |
| CB4 Score <2 | 3794346 | 1612/994503 | 0.42 (0.40-0.45) | 1[Reference] | 1[Reference] | 1[Reference] | 1[Reference] | 1[Reference] | 1[Reference] |
| CB4 Score ≥2 | 955117 | 1622/244956 | 1.79 (1.62-1.97) | 4.00 (3.73-4.28) | 1.62 (1.50-1.75) | 1.61 (1.49-1.74) | 4.10 (3.82-4.39) | 1.66 (1.53-2.79) | 1.64 (1.52-1.78) |
| Model 1 is a crude model. Model 2 is adjusted for sex, year of birth, and educational attainment. Model 3 is adjusted for sex, year of birth, educational attainment, VTE in sibling, Human immunodeficiency virus (HIV) disease, Chronic viral hepatitis B, Chronic viral hepatitis C, Other thrombophilia, Activated protein C resistance [factor V Leiden mutation], Deficiency antithrombin, Deficiency protein C, Deficiency protein S, Prothrombin gene mutation. CB 1= Swedish born, CB2= Born in the Nordic Countries, except Sweden, CB3= born in Europe, except the Nordic Countries, CB4= born outside Europe. Reference for multimorbidity was no or one disease (score=0 or 1). Reference for multimorbidity severity (0-≥5) was no disease (score=0). | | | | | | | | | |

| **Supplementary Table 13.** Landmark 2 stratified for Country of birth (CB). Subdistributional Hazard Ratios (subHR), incidence rates, and incidence rate ratios with 95% confidence intervals (CI), for venous thromboembolism (VTE) according to multimorbidity score ≥2 and Country of birth (CB 1-4) for Landmark 2. | | | | | | | | | | |
| --- | --- | --- | --- | --- | --- | --- | --- | --- | --- | --- |
| **Multimorbidity score** | **Person years, No.** | **Cases (n) /persons at risk (n)** | **Incidence rate, cases/1000 person-years** | **Incidence rate ratio (95%CI)**  **Model 1 Model 2 Model 3** | | | **subHR (95%CI)**  **Model 1 Model 2 Model 3** | | |  |
| **CB 1= Swedish born** | | | | | | | | | | |
| Score 0 | 12658141 | 12560/3202603 | 0.99 (0.98-1.01) | 1[Reference] | 1[Reference] | 1[Reference] | 1[Reference] | 1[Reference] | 1[Reference] |  |
| Score 1 | 5508302 | 8564/3973464 | 1.55 (1.52-1.59) | 1.57 (1.52-1.61) | 1.18 (1.15-1.22) | 1.18 (1.14-1.21) | 1.56 (1.52-1.61) | 1.19 (1.16-1.23) | 1.18 (1.15-1.22) |  |
| Score 2 | 2917468 | 7486/747306 | 2.57 (2.51-2.62) | 2.59 (2.51-2.66) | 1.33 (1.29-1.37) | 1.31 (1.28-1.35) | 2.56 (2.49-2.64) | 1.34 (1.30-1.38) | 1.32 (1.29-1.36) |  |
| Score 3 | 1659070 | 6064/431304 | 3.66 (3.56-3.75) | 3.68 (3.57-3.80) | 1.43 (1.38-1.47) | 1.41 (1.37-1.46) | 3.60 (3.50-3.72) | 1.43 (1.39-1.48) | 1.41 (1.37-1.46) |  |
| Score 4 | 979497 | 4517/259234 | 4.61 (4.48-4.75) | 4.65 (4.49-4.81) | 1.48 (1.43-1.54) | 1.47 (1.42-1.52) | 4.48 (4.34-4.63) | 1.46 (1.41-1.52) | 1.45 (1.39-1.50) |  |
| Score ≥5 | 1312449 | 8957/367478 | 6.82 (6.68–6.97) | 6.88 (6.69-7.07) | 1.70 (1.65-1.75) | 1.68 (1.63-1.73) | 6.28 (6.12-6.46) | 1.57 (1.52-1.62) | 1.56 (1.51-1.60) |  |
| **CB2= Born in the Nordic Countries, except Sweden** | | | | | | | | | | |
| Score 0 | 293957 | 466/81619 | 1.59 (1.45-1.74) | 1[Reference] | 1[Reference] | 1[Reference] | 1[Reference] | 1[Reference] | 1[Reference] |  |
| Score 1 | 110966 | 277/29364 | 2.50 (2.22-2.81) | 1.57 (1.36-1.83) | 1.15 (0.99-1.34) | 1.14 (0.99-1.33) | 1.66 (1.43-1.92) | 1.19 (1.02-1.38) | 1.18 (1.02-1.37) |  |
| Score 2 | 82446 | 284/21825 | 3.44 (3.07-3.87) | 2.17 (1.87-2.51) | 1.31 (1.13-1.52) | 1.30 (1.12-1.51) | 2.29 (1.97-2.65) | 1.35 (1.16-1.57) | 1.34 (1.16-1.56) |  |
| Score 3 | 61060 | 285/16304 | 4.67 (4.16-5.24) | 2.93 (2.54-3.41) | 1.56 (1.13-1.52) | 1.55 (1.34-1.80) | 3.08 (2.66-3.57) | 1.60 (1.37-1.87) | 1.59 (1.36-1.86) |  |
| Score 4 | 41939 | 236/11420 | 5.63 (4.95-6.39) | 3.55 (3.04-4.15) | 1.73 (1.47-2.03) | 1.72 (1.46-2.01) | 3.65 (3.12-4.27) | 1.74 (1.48-2.06) | 1.73 (1.47-2.04) |  |
| Score ≥5 | 70711 | 522/20136 | 7.38 (6.78-8.04) | 4.66 (4.11-5.28) | 1.95 (1.71-2.23) | 1.94 (1.70-2.22) | 4.59 (4.05-5.20) | 1.88 (1.64-2.16) | 1.87 (1.63-2.15) |  |
| **CB3= born in Europe, except the Nordic Countries** | | | | | | | | | | |
| Score 0 | 878839 | 563/237200 | 0.64 (0.59-0.70) | 1[Reference] | 1[Reference] | 1[Reference] | 1[Reference] | 1[Reference] | 1[Reference] |  |
| Score 1 | 170550 | 274/44458 | 1.61 (1.43-1.81) | 2.51 (2.17-2.90) | 1.44 (1.24-1.67) | 1.43 (1.23-1.66) | 2.60 (2.25-3.01) | 1.50 (1.29-1.74) | 1.48 (1.27-1.72) |  |
| Score 2 | 91069 | 239/23799 | 2.62 (2.31-2.98) | 4.10 (3.52-4.77) | 1.67 (1.43-1.95) | 1.65 (1.41-1.93) | 4.25 (3.65-4.94) | 1.73 (1.47-2.04) | 1.71 (1.46-2.02) |  |
| Score 3 | 57681 | 206/15142 | 3.57 (3.12-4.09) | 5.57 (4.75-6.54) | 1.74 (1.47-2.06) | 1.73 (1.46-2.04) | 5.77 (4.92-6.76) | 1.80 (1.51-2.15) | 1.78 (1.49-2.13) |  |
| Score 4 | 35724 | 149/9523 | 4.17 (3.56-4.90) | 6.51 (5.44-7.80) | 1.72 (1.42-2.09) | 1.71 (1.41-2.07) | 6.65 (5.55-7.96) | 1.75 (1.44-2.14) | 1.74 (1.43-2.12) |  |
| Score ≥5 | 53109 | 328/14809 | 6.18 (5.54-6.88) | 9.64 (8.41-11.05) | 1.93 (1.66-2.26) | 1.91 (1.64-2.23) | 9.43 (8.23-10.81) | 1.87 (1.58-2.21) | 1.85 (1.27-2.18) |  |
| **CB4= born outside Europe** | | | | | | | | | | |
| Score 0 | 3100830 | 1131/816720 | 0.36 (0.34-0.39) | 1[Reference] | 1[Reference] | 1[Reference] | 1[Reference] | 1[Reference] | 1[Reference] |  |
| Score 1 | 693517 | 481/177783 | 0.69 (0.63-0.76) | 1.90 (1.71-2.12) | 1.22 (1.10-1.36) | 1.21 (1.09-1.35) | 1.96 (1.76-2.18) | 1.26 (1.13-1.41) | 1.25 (1.12-1.40) |  |
| Score 2 | 385651 | 413/98433 | 1.07 (0.97-1.18) | 2.94 (2.62-3.29) | 1.44 (1.28-1.62) | 1.43 (1.27-1.61) | 3.03 (2.71-3.40) | 1.50 (1.34-1.69) | 1.49 (1.32-1.68) |  |
| Score 3 | 232469 | 357/59369 | 1.54 (1.38-1.70) | 4.21 (3.74-4.74) | 1.69 (1.49-1.91) | 1.67 (1.48-1.90) | 4.35 (3.86-4.90) | 1.76 (1.55-2.00) | 1.75 (1.54-1.98) |  |
| Score 4 | 142921 | 267/36615 | 1.87 (1.66-2.11) | 5.12 (4.48-5.85) | 1.74 (1.51-2.00) | 1.73 (1.50-1.99) | 5.28 (4.63-6.04) | 1.81 (1.57-2.09) | 1.80 (1.55-2.07) |  |
| Score ≥5 | 194075 | 585/50539 | 3.01 (2.78-3.27) | 8.26 (7.48-9.13) | 2.19 (1.96-2.45) | 2.16 (1.93-2.42) | 8.41 (7.61-9.29) | 2.23 (1.98-2.51) | 2.20 (1.95-2.48) |  |
| Abbreviations: Q=quartile. NA=not applicable due to few cases Model 1 is a crude model (univariate). Model 2 is an adjusted model (multivariate), with adjustments for sex, year of birth, and educational attainment. Model 3 is adjusted for sex, year of birth, educational attainment, VTE in sibling, Human immunodeficiency virus (HIV) disease, Chronic viral hepatitis B, Chronic viral hepatitis C, Other thrombophilia, Activated protein C resistance [factor V Leiden mutation], Deficiency antithrombin, Deficiency protein C, Deficiency protein S, Prothrombin gene mutation. Reference with <2 score (one or no disease). | | | | | | | | | | |

| **Supplementary Table 14.**  **Multimorbidity-by-Country of birth interaction of Venous Thromboembolism, including interaction terms Landmark 2.** | | | | | | | |
| --- | --- | --- | --- | --- | --- | --- | --- |
|  | subHR(95%CI) | p-value |  | | | | |
| Multimorbidity | 1.34 (1.31-1.36) | 0.000 |  | | | | |
| CB 2 | 0.90 (0.83-0.96) | 0.003 |  | | | | |
| CB 3 | 0.71 (0.66-0.76) | 0.000 |  | | | | |
| CB 4 | 0.52 (0.50-0.55) | 0.000 |  | | | | |
| Interaction: Multimorbidity x CB 2 | 1.10 (1.00-1.20) | 0.047 |  | | | | |
| Interaction: Multimorbidity x CB 3 | 1.28 (1.16-1.41) | 0.000 |  | | | | |
| Interaction: Multimorbidity x CB 4 | 1.33 (1.24-1.43) | 0.000 |  | | | | |
| **Multimorbidity severity** | **subHR (95% CI)** | **Interaction multimorbidity x CB 2 subHR (95% CI)** | **p-value 2** | **Interaction multimorbidity x CB 3 subHR (95% CI)** | **p-value 3** | **Interaction multimorbidity x CB 4 subHR (95% CI)** | **p-value 4** |
| **1** | 1.19 (1.17-1.22) | 0.98 (0.84-1.14) | 0.79 | 1.34 (1.16-1.55) | 0.000 | 1.09 (0.98-1.22) | 0.1218 |
| **2** | 1.34 (1.30-1.38) | 0.97 (0.84-1.13) | 0.713 | 1.41 (1.21-1.64) | 0.000 | 1.17 (1.04-1.32) | 0.007 |
| **3** | 1.43 (1.38-1.47) | 1.07 (0.92-1.24) | 0.402 | 1.39 (1.18-1.63) | 0.000 | 1.31 (1.16-1.48) | 0.000 |
| **4** | 1.46 (1.41-1.51) | 1.12 (0.96-1.32) | 0.154 | 1.33 (1.11-1.60) | 0.002 | 1.33 (1.16-1.52) | 0.000 |
| **≥5** | 1.56 (1.52-1.61) | 1.11 (0.98-1.27) | 0.099 | 1.33 (1.16-1.53) | 0.000 | 1.54 (1.39-1.71) | 0.000 |
| Model adjusted for sex, year of birth, educational attainment, country of birth, subHR=Subdistributional Hazard Ratios, CI=Confidence interval. CB 1= Swedish born, CB2= Born in the Nordic Countries, except Sweden, CB3= born in Europe, except the Nordic Countries, CB4= born outside Europe. | | | | | | | |

| **Supplementary Table 15.** Landmark 2 multimorbidity severity in F1-F9 disease clusters. Incidence rates, incidence rate ratios, and Subdistributional Hazard Ratios (subHR) with 95% confidence intervals (CI) for VTE (venous thromboembolism) for disease clusters F1-F9 according to multimorbidity score (0 to ≥5) in Landmark 2. All (n=8170329) | | | | | | | | | |
| --- | --- | --- | --- | --- | --- | --- | --- | --- | --- |
| **Score (0 to ≥5)** | **Person years, No.** | **Cases, No./persons at risk, /No** | **Incidence rate, cases/1000 person-years** | **Incidence rate ratio (95%CI)**  **Model 1 Model 2 Model 3** | | | **subHR (95% CI)**  **Model 1 Model 2 Model 3** | | |
| **F1=hypertension, heart failure, coronary heart disease, diabetes, obesity, atrial fibrillation, gout, atherosclerosis, and renal disease (9 diseases)** | | | | | | | | | |
| F1 Score 0 | 26541977 | 30627/6786981 | 1.15 (1.14-1.17) | 1[Reference] | 1[Reference] | 1[Reference] | 1[Reference] | 1[Reference] | 1[Reference] |
| F1 Score 1 | 327993 | 13143/857122 | 4.01 (3.94-4.08) | 3.47 (3.40-3.54) | 1.21 (1.19-1.24) | 1.20 (1.18-1.23) | 3.42 (3.35-3.49) | 1.22 (1.20-1.25) | 1.21 (1.18-1.24) |
| F1 Score 2 | 1242062 | 6824/335306 | 5.49 (5.37-5.63) | 4.76 (4.64-4.89) | 1.25 (1.21-1.28) | 1.24 (1.21-1.28) | 4.55 (4.43-4.67) | 1.22 (1.19-1.26) | 1.22 (1.18-1.25) |
| F1 Score 3 | 470724 | 2995/131294 | 6.36 (6.14-6.59) | 5.51 (5.31-5.72) | 1.32 (1.27-1.37) | 1.32 (1.27-1.37) | 5.11 (4.92-5.30) | 1.24 (1.19-1.29) | 1.24 (1.19-1.29) |
| F1 Score 4 | 143397 | 1080/42193 | 7.52 (7.10-7.99) | 6.53 (6.14-6.94) | 1.40 (1.31-1.48) | 1.41 (1.32-1.50) | 5.74 (5.40-6.10) | 1.23 (1.16-1.31) | 1.24 (1.17-1.32) |
| F1 Score ≥5 | 54348 | 542/17433 | 9.97 (9.17-10.85) | 8.64 (7.94-9.41) | 1.66 (1.53-1.81) | 1.67 (1.53-1.82) | 6.99 (6.42-7.62) | 1.34 (1.23-1.46) | 1.35 (1.24-1.47) |
| **F2=affective disorders, anxiety, psychoactive substance misuse, alcohol misuse disorders, anorexia or bulimia, and schizophrenia disorders (6 diseases)** | | | | | | | | |  |
| F2 Score 0 | 27490646 | 43768/7070216 | 1.59 (1.58-1.61) | 1[Reference] | 1[Reference] | 1[Reference] | 1[Reference] | 1[Reference] | 1[Reference] |
| F2 Score 1 | 2884251 | 7597/748307 | 2.63 (2.58-2.69) | 1.65 (1.61-1.70) | 1.24 (1.21-1.27) | 1.24 (1.21-1.27) | 1.64 (1.60-1.68) | 1.20 (1.17-1.23) | 1.20 (1.17-1.23) |
| F2 Score 2 | 1210920 | 3279/313284 | 2.71 (2.62-2.80) | 1.70 (1.64-1.76) | 1.39 (1.34-1.44) | 1.39 (1.34-1.44) | 1.69 (1.64-1.76) | 1.36 (1.31-1.40) | 1.35 (1.31-1.40) |
| F2 Score 3 | 128004 | 475/33548 | 3.71 (3.39-4.06) | 2.33 (2.13-2.55) | 2.20 (2.01-2.41) | 2.06 (1.88-2.26) | 2.30 (2.10-2.51) | 2.13 (1.95-2.34) | 2.00 (1.83-2.19) |
| F2 Score 4 | 17861 | 90/4772 | 5.04 (4.10-6.20) | 3.16 (2.57-3.89) | 3.64 (2.96-4.48) | 3.19 (2.59-3.93) | 3.07 (2.50-3.78) | 3.43 (2.79-4.23) | 3.00 (2.43-3.70) |
| F2 Score ≥5 | 760 | 2/202 | 2.63 (0.066-10.52) | 1.65 (0.41-6.61) | 3.01 (0.75-12.02) | 2.62 (0.65-10.46) | 1.61 (0.40-6.44) | 2.80 (0.69-11.29) | 2.41 (0.59-9.74) |
| **F3=inflammatory bowel disease, liver disease, pancreatic disease, and ulcers (4 diseases)** | | | | | | | | | |
| F3 Score 0 | 31427776 | 53783/8089094 | 1.71 (1.70-1.73) | 1[Reference] | 1[Reference] | 1[Reference] | 1[Reference] | 1[Reference] | 1[Reference] |
| F3 Score 1 | 295428 | 1368/78653 | 4.63 (4.39-4.88) | 2.71 (2.56-2.86) | 1.51 (1.43-1.60) | 1.50 (1.42-1.58) | 2.63 (2.49-2.78) | 1.47 (1.39-1.55) | 1.45 (1.37-1.53) |
| F3 Score 2 | 8866 | 55/2580 | 6.20 (4.76-8.08) | 3.62 (2.78-4.72) | 1.99 (1.53-2.59) | 1.89 (1.45-2.47) | 3.37 (2.58-4.39) | 1.83 (1.40-2.38) | 1.73 (1.33-2.26) |
| F3 Score 3 | 371 | 5/102 | 13.46 (5.60-32.34) | 7.87 (3.27-18.90) | 4.63 (1.93-11.13) | 4.19 (1.75-10.08) | 7.55 (3.15-18.11) | 4.62 (1.93-11.02) | 4.19 (1.75-10.06) |
| F3 Score 4 | NA | NA | NA | NA | NA | NA | NA | NA | NA |
| F3 Score ≥5 | NA | NA | NA | NA | NA | NA | NA | NA | NA |
| **F4=epilepsy, blindness and poor vision, cerebrovascular disease, cancer, and impaired or hearing loss (5 diseases)** | | | | | | | | | |
| F4 Score 0 | 29694169 | 43855/7612874 | 1.48 (1.46-1.49) | 1[Reference] | 1[Reference] | 1[Reference] | 1[Reference] | 1[Reference] | 1[Reference] |
| F4 Score 1 | 1788828 | 9281/483593 | 5.19 (5.08-5.29) | 3.51 (3.45-3.59) | 1.28 (1.25-1.31) | 1.28 (1.25-1.31) | 3.36 (3.28-3.43) | 1.23 (1.20-1.26) | 1.23 (1.20-1.26) |
| F4 Score 2 | 221129 | 1789/64866 | 8.09 (7.72-8.47) | 5.48 (5.22-5.74) | 1.31 (1.24-1.37) | 1.32 (1.26-1.38) | 4.85 (4.62-5.08) | 1.17 (1.11-1.22) | 1.18 (1.13-1.24) |
| F4 Score 3 | 26172 | 260/8276 | 9.93 (8.80-11.22) | 6.73 (5.95-7.60) | 1.35 (1.20-1.53) | 1.37 (1.21-1.55) | 5.53 (4.90-6.25) | 1.12 (0.99-1.26) | 1.14 (1.01-1.29) |
| F4 Score 4 | 2093 | 26/701 | 12.42 (8.46-18.24) | 8.41 (5.73-12.36) | 1.64 (1.12-2.41) | 1.67 (1.14-2.46) | 6.55 (4.46-9.62) | 1.25 (0.85-1.84) | 1.28 (0.87-1.90) |
| F4 Score ≥5 | 60 | 0/19 | 0 | - | - | - | - | - | - |
| **F5=connective tissue disease, osteoporosis, thyroid disorders, and psoriasis; (4 diseases)** | | | | | | | | | |
| F5 Score 0 | 29972302 | 47399/7702980 | 1.58 (1.57-1.60) | 1[Reference] | 1[Reference] | 1[Reference] | 1[Reference] | 1[Reference] | 1[Reference] |
| F5 Score 1 | 1599797 | 6686/422979 | 4.18 (4.08-4.28) | 2.63 (2.58-2.71) | 1.19 (1.16-1.22) | 1.19 (1.15-1.22) | 2.58 (2.52-2.65) | 1.17 (1.14-1.20) | 1.17 (1.14-1.20) |
| F5 Score 2 | 147562 | 1018/40742 | 6.90 (6.49-7.34) | 4.36 (4.10-4.64) | 1.38 (1.29-1.47) | 1.37 (1.28-1.46) | 4.10 (3.86-4.37) | 1.32 (1.24-1.40) | 1.31 (1.23-1.40) |
| F5 Score 3 | 12245 | 103/3491 | 8.41 (6.93-10.20) | 5.32 (4.38-6.45) | 1.47 (1.21-1.78) | 1.45 (1.19-1.76) | 4.86 (4.00-5.90) | 1.38 (1.13-1.67) | 1.36 (1.12-1.65) |
| F5 Score 4 | 536 | 5/155 | 9.33 (3.88-22.41) | 5.90 (2.46-14.17) | 1.52 (0.63-3.65) | 1.50 (0.62-3.59) | 5.32 (2.21-12.82) | 1.42 (0.59-3.41) | 1.40 (0.58-3.36) |
| F5 Score ≥5 | NA | NA | NA | NA | NA | NA | NA | NA | NA |
| **F6=prostate disease, arthrosis, painful back condition, diverticular disease of intestine, and chronic sinusitis (5 diseases)** | | | | | | | | | |
| F6 Score 0 | 25584561 | 32824/6568412 | 1.28 (1.27-1.30) | 1[Reference] | 1[Reference] | 1[Reference] | 1[Reference] | 1[Reference] | 1[Reference] |
| F6 Score 1 | 5056996 | 16000/1311029 | 3.16 (3.12-3.21) | 2.47 (2.42-2.51) | 1.23 (1.20-1.25) | 1.23 (1.21-1.25) | 2.45 (2.41-2.50) | 1.24 (1.21-1.26) | 1.24 (1.21-1.26) |
| F6 Score 2 | 962488 | 5391/256070 | 5.60 (5.45-5.75) | 4.37 (4.24-4.49) | 1.30 (1.26-1.34) | 1.31 (1.27-1.35) | 4.25 (4.13-4.37) | 1.31 (1.28-1.35) | 1.32 (1.28-1.36) |
| F6 Score 3 | 121392 | 948/32906 | 7.81 (7.33-8.32) | 6.09 (5.71-6.49) | 1.42 (1.33-1.52) | 1.42 (1.33-1.52) | 5.84 (5.47-6.23) | 1.43 (1.34-1.53) | 1.44 (1.35-1.54) |
| F6 Score 4 | 6928 | 48/1891 | 6.93 (5.22-9.19) | 5.40 (4.07-7.17) | 1.16 (0.87-1.54) | 1.15 (0.86-1.52) | 5.13 (3.86-6.80) | 1.16 (0.87-1.54) | 1.15 (0.86-1.53) |
| F6 Score ≥5 | 76 | 0/21 | 0 | 0 | - | - | - | - | - |
| **F7=bronchiectasis, Parkinson’s disease, glaucoma, learning disability, and irritable bowel syndrome (5 diseases)** | | | | | | | | | |
| F7 Score 0 | 30952041 | 52536/7964603 | 1.70 (1.68-1.71) | 1[Reference] | 1[Reference] | 1[Reference] | 1[Reference] | 1[Reference] | 1[Reference] |
| F7 Score 1 | 769538 | 2612/202673 | 3.39 (3.27-3.52) | 2.00 (1.92-2.08) | 1.12 (1.07-1.16) | 1.12 (1.08-1.17) | 1.96 (1.88-2.04) | 1.07 (1.03-1.12) | 1.08 (1.04-1.12) |
| F7 Score 2 | 10729 | 61/3013 | 5.69 (4.42-7.31) | 3.35 (2.61-4.31) | 1.05 (0.82-1.36) | 1.06 (0.83-1.37) | 3.09 (2.41-3.98) | 0.98 (0.76-1.26) | 0.99 (0.77-1.27) |
| F7 Score 3 | 133 | 2/40 | 14.99 (3.75-59.96) | 8.83 (2.21-35.32) | 2.29 (0.57-9.14) | 2.34 (0.59-9.38) | 7.90 (1.92-32.47) | 2.12 (0.51-8.84) | 2.17 (0.52-9.07) |
| F7 Score 4 | NA | NA | NA | NA | NA | NA | NA | NA | NA |
| F7 Score ≥5 | NA | NA | NA | NA | NA | NA | NA | NA | NA |
| **F8=asthma, dermatitis and eczema, constipation, chronic obstructive pulmonary disease, and migraine (5 diseases)** | | | | | | | | | |
| F8 Score 0 | 25218161 | 39962/6487474 | 1.58 (1.57-1.60) | 1[Reference] | 1[Reference] | 1[Reference] | 1[Reference] | 1[Reference] | 1[Reference] |
| F8 Score 1 | 5419398 | 11921/1396355 | 2.20 (2.16-2.24) | 1.39 (1.36-1.42) | 1.21 (1.19-1.24) | 1.21 (1.19-1.24) | 1.39 (1.36-1.42) | 1.19 (1.16-1.21) | 1.18 (1.16-1.21) |
| F8 Score 2 | 970209 | 2744/253105 | 2.83 (2.74-2.94) | 1.78 (1.72-1.86) | 1.39 (1.34-1.45) | 1.40 (1.34-1.45) | 1.76 (1.70-1.83) | 1.32 (1.27-1.37) | 1.32 (1.27-1.38) |
| F8 Score 3 | 116156 | 502/31011 | 4.32 (3.96-4.72) | 2.72 (2.50-2.98) | 1.66 (1.52-1.81) | 1.67 (1.53-1.82) | 2.64 (2.42-2.89) | 1.54 (1.41-1.68) | 1.55 (1.42-1.69) |
| F8 Score 4 | 8258 | 80/2317 | 9.69 (7.78-12.06) | 6.11 (4.91-7.61) | 2.68 (2.15-3.34) | 2.75 (2.21-3.42) | 5.69 (4.57-7.09) | 2.39 (1.91-2.98) | 2.45 (1.96-3.06) |
| F8 Score ≥5 | 260 | 2/67 | 7.69 (1.92-30.73) | 4.85 (1.21-19.40) | 2.73 (0.68-10.90) | 2.59 (0.65-10.34) | 4.90 (1.23-19.52) | 2.87 (0.73-11.35) | 2.72 (0.68-10.82) |
| **F9=multiple sclerosis and dementia (2 diseases)** | | | | | | | | | |
| F9 Score 0 | 31557788 | 53851/8108627 | 1.71 (1.69-1.72) | 1[Reference] | 1[Reference] | 1[Reference] | 1[Reference] | 1[Reference] | 1[Reference] |
| F9 Score 1 | 174144 | 1355/61529 | 7.78 (7.38-8.21) | 4.56 (4.32-4.81) | 1.05 (0.99-1.11) | 1.07 (1.01-1.13) | 3.35 (3.17-3.53) | 0.74 (0.70-1.78) | 0.76 (0.72-0.80) |
| F9 Score 2 | 510 | 5/173 | 9.81 (4.08-23.58) | 5.75 (2.39-13.82) | 1.69 (0.70-4.07) | 1.66 (0.69-3.98) | 4.42 (1.84-10.63) | 1.32 (0.55-3.15) | 1.28 (0.53-3.09) |
| F9 Score 3 | NA | NA | NA | NA | NA | NA | NA | NA | NA |
| F9 Score 4 | NA | NA | NA | NA | NA | NA | NA | NA | NA |
| F9 Score ≥5 | NA | NA | NA | NA | NA | NA | NA | NA | NA |
| Abbreviations: NA=not applicable due to few cases. Model 1 is a crude model. Model 2 is adjusted for sex, year of birth and educational attainment. Model 3 is adjusted for sex, year of birth, educational attainment, country of birth, VTE in sibling, Human immunodeficiency virus (HIV) disease, Chronic viral hepatitis B, Chronic viral hepatitis C, Other thrombophilia, Activated protein C resistance [factor V Leiden mutation], Deficiency antithrombin, Deficiency protein C, Deficiency protein S, Prothrombin gene mutation. Reference with no disease (score=0). | | | | | | | | | |

| **Supplementary Table 16.** Landmark 2 stratified for Chronic viral hepatitis C (B182). Subdistribution Hazard Ratios (subHR), incidence rates, and incidence rate ratios with 95% confidence intervals (CI), for venous thromboembolism (VTE) according to multimorbidity score ≥2 and Chronic viral hepatitis C (B182) for Landmark 2. | | | | | | | | | |
| --- | --- | --- | --- | --- | --- | --- | --- | --- | --- |
| **Multimorbidity score** | **Person years, No.** | **Cases, No./persons at risk, /No** | **Incidence rate, cases/1000 person-years** | **Incidence rate ratio (95%CI)**  **Model 1 Model 2 Model 3** | | | **HR (95%CI)**  **Model 1 Model 2 Model 3** | | |
| B182 <2 | 36330 | 97/9505 | 2.67 (2.19-3.26) | 1[Reference] | 1[Reference] | 1[Reference] | 1[Reference] | 1[Reference] | 1[Reference] |
| B182 ≥2 | 60289 | 312/16214 | 5.18 (4.63-5.78) | 1.94 (1.54-2.43) | 1.83 (1.45-2.31) | 1.80 (1.43-2.26) | 1.89 (1.51-2.38) | 1.80 (1.43-2.27) | 1.77 (1.40-2.22) |
| Without Score <2 | 23378771 | 24219/5977588 | 1.04 (1.03-1.05) | 1[Reference] | 1[Reference] | 1[Reference] | 1[Reference] | 1[Reference] | 1[Reference] |
| Without Score ≥2 | 8255705 | 30583/2167022 | 3.70 (3.67-3.76) | 3.58 (3.52-3.64) | 1.39 (1.36-1.41) | 1.39 (1.36-1.41) | 3.50 (3.44-3.56) | 1.36 (1.34-1.39) | 1.36 (1.33-1.39) |
| **Chronic viral hepatitis C (B182)** | | | | | | | | | |
| Score 0 | 18979 | 40/4958 | 2.11 (1.55-2.87) | 1[Reference] | 1[Reference] | 1[Reference] | 1[Reference] | 1[Reference] | 1[Reference] |
| Score 1 | 17351 | 57/4547 | 3.29 (2.53-4.26) | 1.56 (1.04-2.34) | 1.54 (1.03-2.31) | 1.51 (1.01-2.26) | 1.56 (1.04-2.34) | 1.54 (1.03-2.31) | 1.51 (1.01-2.62) |
| Score 2 | 15984 | 54/4195 | 3.38 (2.59-4.41) | 1.60 (1.06-2.41) | 1.56 (1.04-2.35) | 1.53 (1.01-2.30) | 1.60 (1.06-2.41) | 1.57 (1.04-2.36) | 1.53 (1.02-2.31) |
| Score 3 | 13850 | 69/3701 | 4.98 (3.93-6.31) | 2.36 (1.60-3.49) | 2.28 (1.54-3.37) | 2.23 (1.51-3.29) | 2.32 (1.57-3.43) | 2.27 (1.53-3.34) | 2.20 (1.49-3.25) |
| Score 4 | 10663 | 50/2845 | 4.67 (3.56-6.19) | 2.22 (1.47-3.37) | 2.12 (1.40-3.22) | 2.06 (1.35-3.12) | 2.19 (1.46-3.32) | 2.11 (1.39-3.21) | 2.05 (1.35-3.10) |
| Score ≥5 | 19792 | 139/5473 | 7.02 (5.95-8.29) | 3.33 (2.34-4.74) | 3.10 (2.17-4.43) | 2.97 (2.08-4.25) | 3.18 (2.23-4.51) | 3.00 (2.10-4.27) | 2.87 (2.01-4.08) |
| **Without** | | | | | | | | | |
| Score 0 | 16912787 | 14680/433184 | 0.87 (0.86-0.88) | 1[Reference] | 1[Reference] | 1[Reference] | 1[Reference] | 1[Reference] | 1[Reference] |
| Score 1 | 6465984 | 9539/1644404 | 1.48 (1.45-1.51) | 1.70 (1.66-1.75) | 1.21 (1.18-1.24) | 1.19 (1.16-1.22) | 1.71 (1.67-1.76) | 1.23 (1.20-1.26) | 1.20 (1.17-1.24) |
| Score 2 | 3460650 | 8368/887168 | 2.42 (2.37-2.47) | 2.79 (2.71-2.86) | 1.36 (1.32-1.39) | 1.33 (1.30-1.37) | 2.79 (2.72-2.87) | 1.38 (1.34-1.42) | 1.35 (1.32-1.39) |
| Score 3 | 1996431 | 6843/518418 | 3.43 (3.36-3.52) | 3.96 (3.84-4.07) | 1.46 (1.42-1.50) | 1.44 (1.40-1.49) | 3.92 (3.81-4.03) | 1.47 (1.43-1.52) | 1.45 (1.41-1.50) |
| Score 4 | 1189418 | 5119/313947 | 4.31 (4.19-4.44) | 4.95 (4.80-5.11) | 1.51 (1.46-1.56) | 1.50 (1.45-1.55) | 4.85 (4.69-5.00) | 1.50 (1.45-1.55) | 1.49 (1.44-1.54) |
| Score ≥5 | 1610552 | 10253/447489 | 6.37 (6.25-6.50) | 7.33 (7.15-7.52) | 1.72 (1.67-1.77) | 1.72 (1.68-1.78) | 6.83 (6.66-7.01) | 1.61 (1.56-1.66) | 1.61 (1.56-1.66) |
| Model 1 is a crude model. Model 2 is adjusted for sex, year of birth, and educational attainment. Model 3 is adjusted for sex, year of birth, educational attainment, VTE in sibling, Human immunodeficiency virus (HIV) disease, Chronic viral hepatitis B, Other thrombophilia, Activated protein C resistance [factor V Leiden mutation], Deficiency antithrombin, Deficiency protein C, Deficiency protein S, Prothrombin gene mutation. Reference for multimorbidity was no or one disease (score=0 or 1). Reference for multimorbidity severity (0-≥5) was no disease (score=0). | | | | | | | | | |

| **Supplementary Table 17.** Landmark 2 stratified for Human immunodeficiency virus (HIV) (B20-24). Subdistribution Hazard Ratios (subHR), incidence rates, and incidence rate ratios with 95% confidence intervals (CI), for venous thromboembolism (VTE) according to multimorbidity score ≥2 and Human immunodeficiency virus (HIV) (B20-24) for Landmark 2. | | | | | | | | | |
| --- | --- | --- | --- | --- | --- | --- | --- | --- | --- |
| **Multimorbidity score** | **Person years, No.** | **Cases, No./persons at risk, /No** | **Incidence rate, cases/1000 person-years** | **Incidence rate ratio (95%CI)**  **Model 1 Model 2 Model 3** | | | **HR (95%CI)**  **Model 1 Model 2 Model 3** | | |
| B2024 <2 | 13467 | 26/3489 | 1.93 (1.31-2.84) | 1[Reference] | 1[Reference] | 1[Reference] | 1[Reference] | 1[Reference] | 1[Reference] |
| B2024 ≥2 | 8626 | 25/2521 | 2.90 (1.96-4.29) | 1.50 (0.87-2.60) | 1.03 (0.58-1.82) | 0.94 (0.53-1.67) | 1.49 (0.86-2.58) | 1.02 (0.58-1.81) | 0.93 (0.52-1.68) |
| Without Score <2 | 23401634 | 24290/5983604 | 1.04 (1.03-1.05) | 1[Reference] | 1[Reference] | 1[Reference] | 1[Reference] | 1[Reference] | 1[Reference] |
| Without Score ≥2 | 8308714 | 30870/2180985 | 3.71 (3.67-3.76) | 3.58 (3.52-3.64) | 1.40 (1.37-1.42) | 1.39 (1.36-1.41) | 3.51 (3.45-3.56) | 1.37 (1.35-1.40) | 1.36 (1.34-1.39) |
| **Human immunodeficiency virus (HIV) (B20-24)** | | | | | | | | | |
| Score 0 | 8735 | 16/2264 | 1.83 (1.12-2.99) | 1[Reference] | 1[Reference] | 1[Reference] | 1[Reference] | 1[Reference] | 1[Reference] |
| Score 1 | 4732 | 10/1225 | 2.11 (1.14-3.93) | 1.15 (0.52-2.54) | 1.00 (0.45-2.21) | 0.94 (0.42-2.09) | 1.16 (0.52-2.55) | 1.01 (0.46-2.23) | 0.95 (0.43-2.09) |
| Score 2 | 3330 | 6/853 | 1.80 (0.81-4.01) | 0.98 (0.38-2.51) | 0.76 (0.30-1.95) | 0.69 (0.27-1.78) | 0.99 (0.39-2.54) | 0.78 (0.30-2.02) | 0.71 (0.27-1.83) |
| Score 3 | 1988 | 5/511 | 2.51 (1.05-6.04) | 1.37 (0.50-3.75) | 0.95 (0.35-2.63) | 0.85 (0.30-2.35) | 1.38 (0.51-3.78) | 0.98 (0.35-2.72) | 0.86 (0.30-2.49) |
| Score 4 | 1261 | 2/327 | 1.59 (0.30-6.34) | 0.87 (0.20-3.77) | 0.55 (0.12-2.41) | 0.49 (0.11-2.15) | 0.87 (0.19-3.77) | 0.56 (0.13-2.43) | 0.49 (0.11-2.21) |
| Score ≥5 | 2046 | 12/560 | 5.87 (3.33-10.32) | 3.20 (1.52-6.77) | 1.76 (0.79-3.93) | 1.53 (0.68-3.44) | 3.06 (1.45-6.46) | 1.67 (0.76-3.69) | 1.44 (0.64-3.26) |
| **Without** | | | | | | | | | |
| Score 0 | 16923030 | 14704/435878 | 0.87 (0.86-0.88) | 1[Reference] | 1[Reference] | 1[Reference] | 1[Reference] | 1[Reference] | 1[Reference] |
| Score 1 | 6478604 | 9586/1647726 | 1.48 (1.45-1.51) | 1.70 (1.66-1.75) | 1.22 (1.19-1.25) | 1.19 (1.16-1.22) | 1.72 (1.67-1.76) | 1.24 (1.20-1.27) | 1.21 (1.18-1.24) |
| Score 2 | 3473303 | 8416/890510 | 2.42 (2.37-2.47) | 2.79 (2.71-2.86) | 1.36 (1.33-1.40) | 1.34 (1.30-1.37) | 2.80 (2.72-2.87) | 1.39 (1.35-1.43) | 1.36 (1.32-1.24) |
| Score 3 | 2008292 | 6907/521608 | 3.43 (3.36-3.52) | 3.96 (3.84-4.07) | 1.47 (1.43-1.51) | 1.45 (1.41-1.49) | 3.92 (3.81-4.04) | 1.49 (1.44-1.53) | 1.46 (1.41-1.50) |
| Score 4 | 1198820 | 5167/316465 | 4.31 (4.19-4.44) | 4.95 (4.80-5.11) | 1.52 (1.47-1.57) | 1.50 (1.46-1.55) | 4.85 (4.69-5.00) | 1.52 (1.47-1.57) | 1.49 (1.44-1.54) |
| Score ≥5 | 1628298 | 10380/452402 | 6.37 (6.25-6.50) | 7.33 (7.15-7.52) | 1.74 (1.69-1.79) | 1.73 (1.68-1.78) | 6.84 (6.67-7.01) | 1.63 (1.58-1.68) | 1.62 (1.57-1.67) |
| Model 1 is a crude model. Model 2 is adjusted for sex, year of birth, and educational attainment. Model 3 is adjusted for sex, year of birth, educational attainment, VTE in sibling, Chronic viral hepatitis B, Chronic viral hepatitis C, Other thrombophilia, Activated protein C resistance [factor V Leiden mutation], Deficiency antithrombin, Deficiency protein C, Deficiency protein S, Prothrombin gene mutation. Reference for multimorbidity was no or one disease (score=0 or 1). Reference for multimorbidity severity (0-≥5) was no disease (score=0). | | | | | | | | | |

| **Supplementary Table 18.** Landmark 2 stratified for Chronic viral hepatitis B (B18.0, B18.1). Subdistribution Hazard Ratios (subHR), incidence rates, and incidence rate ratios with 95% confidence intervals (CI), for venous thromboembolism (VTE) according to multimorbidity score ≥2 and Chronic viral hepatitis B (B18.0, B18.1) for Landmark 2. | | | | | | | | | |
| --- | --- | --- | --- | --- | --- | --- | --- | --- | --- |
| **Multimorbidity score** | **Person years, No.** | **Cases, No./persons at risk, /No** | **Incidence rate, cases/1000 person-years** | **Incidence rate ratio (95%CI)**  **Model 1 Model 2 Model 3** | | | **HR (95%CI)**  **Model 1 Model 2 Model 3** | | |
| B180181 <2 | 28151 | 27/7210 | 0.96 (0.66-1.40) | 1[Reference] | 1[Reference] | 1[Reference] | 1[Reference] | 1[Reference] | 1[Reference] |
| B180181 ≥2 | 20455 | 78/5359 | 3.81 (3.05-4.76) | 3.96 (2.57-6.16) | 2.95 (1.85-4.69) | 2.47 (1.55-3.95) | 3.91 (2.52-6.06) | 2.95 (1.82-4.76) | 2.47 (1.52-4.03) |
| Without Score <2 | 23338694 | 24289/5979883 | 1.04 (1.03-1.05) | 1[Reference] | 1[Reference] | 1[Reference] | 1[Reference] | 1[Reference] | 1[Reference] |
| Without Score ≥2 | 8296885 | 30817/2177877 | 3.71 (3.67-3.76) | 3.58 (3.52-3.64) | 139 (1.37-1.42) | 1.39 (1.36-1.41) | 3.50 (3.44-3.56) | 1.37 (1.35-1.40) | 1.36 (1.34-1.39) |
| **Chronic viral hepatitis B (B18.0, B18.1)** | | | | | | | | | |
| Score 0 | 17397 | 10/4457 | 0.57 (0.41-1.07) | 1[Reference] | 1[Reference] | 1[Reference] | 1[Reference] | 1[Reference] | 1[Reference] |
| Score 1 | 10755 | 17/2753 | 1.58 (0.98-2.54) | 2.75 (1.26-6.01) | 2.53 (1.16-5.55) | 2.38 (1.09-5.21) | 2.76 (1.26-6.02) | 2.55 (1.18-5.55) | 2.42 (1.12-5.23) |
| Score 2 | 7258 | 18/1872 | 2.48 (1.56-3.94) | 4.31 (1.99-9.35) | 3.65 (1.67-7.98) | 3.26 (1.49-7.14) | 4.30 (1.99-9.32) | 3.70 (1.70-8.06) | 3.30 (1.53-7.16) |
| Score 3 | 4690 | 20/1220 | 4.27 (1.56-6.61) | 7.42 (3.47-15.85) | 5.73 (2.64-12.44) | 4.74 (2.18-10.32) | 4.30 (3.45-15.74) | 5.80 (2.71-12.44) | 4.79 (1.53-20.20) |
| Score 4 | 2998 | 10/776 | 3.33 (1.79-6.20) | 5.80 (2.42-13.94) | 4.19 (1.71-10.30) | 3.34 (1.35-8.23) | 5.77 (2.40-13.85) | 4.30 (2.91-12.66) | 3.45 (1.39-8.58) |
| Score ≥5 | 5510 | 30/1491 | 5.44 (3.81-7.79) | 9.47 (4.63-19.38) | 6.18 (2.88-13.23) | 4.53 (2.10-9.77) | 9.06 (4.43-18.52) | 6.07 (2.91-12.66) | 4.48 (2.15-9.33) |
| **Without** | | | | | | | | | |
| Score 0 | 16914369 | 14710/433685 | 0.87 (0.86-0.88) | 1[Reference] | 1[Reference] | 1[Reference] | 1[Reference] | 1[Reference] | 1[Reference] |
| Score 1 | 6472581 | 9579/1646198 | 1.48 (1.45-1.51) | 1.70 (1.66-1.75) | 1.22 (1.18-1.25) | 1.19 (1.16-1.22) | 1.72 (1.67-1.76) | 1.23 (1.20-1.27) | 1.20 (1.17-1.24) |
| Score 2 | 3469376 | 8404/889491 | 2.42 (2.37-2.47) | 2.79 (2.71-2.86) | 1.36 (1.33-1.40) | 1.34 (1.30-1.37) | 2.79 (2.72-2.87) | 1.38 (1.35-1.42) | 1.35 (1.32-1.39) |
| Score 3 | 2005591 | 6892/520899 | 3.43 (3.36-3.52) | 3.96 (3.84-4.07) | 1.47 (1.43-1.51) | 1.45 (1.41-1.49) | 3.92 (3.81-4.03) | 1.48 (1.44-1.53) | 1.45 (1.41-1.50) |
| Score 4 | 1197084 | 5159/316016 | 4.31 (4.19-4.44) | 4.95(4.80-5.11) | 1.52 (1.47-1.57) | 1.50 (1.46-1.55) | 4.84 (4.69-5.00) | 1.51 (1.46-1.56) | 1.49 (1.44-1.54) |
| Score ≥5 | 1624835 | 10362/451471 | 6.37 (6.25-6.50) | 7.33 (7.15-7.52) | 1.74 (1.69-1.79) | 1.73 (1.68-1.78) | 6.83 (6.66-7.01) | 1.62 (1.58-1.67) | 1.61 (1.56-1.66) |
| Model 1 is a crude model. Model 2 is adjusted for sex, year of birth, and educational attainment. Model 3 is adjusted for sex, year of birth, educational attainment, VTE in sibling, Human immunodeficiency virus (HIV) disease, Chronic viral hepatitis C, Other thrombophilia, Activated protein C resistance [factor V Leiden mutation], Deficiency antithrombin, Deficiency protein C, Deficiency protein S, Prothrombin gene mutation. Reference for multimorbidity was no or one disease (score=0 or 1). Reference for multimorbidity severity (0-≥5) was no disease (score=0). | | | | | | | | | |

| **Supplementary Table 19.** Landmark 2 stratified for Prothrombin gene mutation (D68.5E). Subdistribution Hazard Ratios (subHR), incidence rates, and incidence rate ratios with 95% confidence intervals (CI), for venous thromboembolism (VTE) according to multimorbidity score ≥2 and Prothrombin gene mutation (D68.5E) for Landmark 2. | | | | | | | | | |
| --- | --- | --- | --- | --- | --- | --- | --- | --- | --- |
| **Multimorbidity score** | **Person years, No.** | **Cases, No./persons at risk, /No** | **Incidence rate, cases/1000 person-years** | **Incidence rate ratio (95%CI)**  **Model 1 Model 2 Model 3** | | | **subHR (95%CI)**  **Model 1 Model 2 Model 3** | | |
| D68.5E <2 | 400 | 5/175 | 10 (3.76-26.67) | 1[Reference] | 1[Reference] | 1[Reference] | 1[Reference] | 1[Reference] | 1[Reference] |
| D68.5E ≥2 | 300 | 1/76 | 3.33 (0.47-23.64) | 0.33 (0.037-2.98) | 0.28 (0.027-2.96) | 0.035 (0.0039-3.12) | 0.34 (0.38-3.02) | 0.33 (0.24-4.46) | 0.13 (0.073-21.57) |
| Without Score <2 | 23414701 | 24312/5986988 | 1.04 (1.03-1.05) | 1[Reference] | 1[Reference] | 1[Reference] | 1[Reference] | 1[Reference] | 1[Reference] |
| Without Score ≥2 | 8317040 | 30894/2183160 | 3.71 (3.67-3.76) | 3.58 (3.51-3.64) | 1.40 (1.37-1.43) | 1.39 (1.36-1.41) | 3.50 (3.45-3.56) | 1.37 (1.35-1.40) | 1.36 (1.34-1.39) |
| **Prothrombin gene mutation (D68.5E)** | | | | | | | | | |
| Score 0 | 241 | 2/63 | 0.083 (0.021-3.32) | 1[Reference] | 1[Reference] | 1[Reference] | 1[Reference] | 1[Reference] | 1[Reference] |
| Score 1 | 259 | 2/42 | 1.26 (0.31-5.03) | 1.51 (0.21-10.75) | 1.84 (0.26-13.21) | 1.73 (0.23-13.02) | 1.54 (0.22-10.89) | 2.03 (0.37-11.09) | 1.77 (0.39-98.06) |
| Score 2 | 103 | 1/26 | 0.097 (0.014-6.88) | 1.17 (011-12.87) | 1.55 (0.14-17.84) | 0.12 (0.0038-41.36) | 1.21 (0.11-13.00) | 1.69 (0.19-14.91) | 0.43 (0.066-279.60) |
| Score 3 | 84 | 0/21 | - | - | - | - | - | - | - |
| Score 4 | 37 | 0/10 | - | - | - | - | - | - | - |
| Score ≥5 | 76 | 0/19 | - | - | - | - | - | - | - |
| **Without** | | | | | | | | | |
| Score 0 | 16931525 | 14718/4338079 | 0.87 (0.86-0.88) | 1[Reference] | 1[Reference] | 1[Reference] | 1[Reference] | 1[Reference] | 1[Reference] |
| Score 1 | 6483177 | 9594/1648909 | 1.48 (1.45-1.51) | 1.70 (1.66-1.75) | 1.22 (1.19-1.25) | 1.19 (1.16-1.22) | 1.72 (1.67-1.76) | 1.24 (1.20-1.27) | 1.21 (1.17-1.24) |
| Score 2 | 3476531 | 8421/891337 | 2.42 (2.37-2.47) | 2.79 (2.71-2.86) | 1.36 (1.33-1.40) | 1.34 (1.30-1.37) | 2.79 (2.72-2.87) | 1.39 (1.35-1.43) | 1.35 (1.32-1.39) |
| Score 3 | 2010197 | 6912/522098 | 3.44 (3.34-3.52) | 3.96 (3.84-4.07) | 1.47 (1.43-1.51) | 1.45 (1.41-1.49) | 3.92 (3.81-4.04) | 1.48 (1.44-1.53) | 1.46 (1.41-1.50) |
| Score 4 | 1200044 | 5169/316782 | 4.31 (4.19-4.43) | 4.96 (4.80-5.11) | 1.52 (1.47-1.57) | 1.50 (1.46-1.55) | 4.84 (4.69-5.00) | 1.52 (1.47-1.57) | 1.49 (1.44-1.54) |
| Score ≥5 | 1630268 | 10392/452943 | 6.37 (6.25-6.50) | 7.33 (7.15-7.52) | 1.74 (1.69-1.79) | 1.73 (1.68-1.78) | 6.83 (6.66-7.01) | 1.63 (1.58-1.68) | 1.62 (1.57-1.67) |
| Model 1 is a crude model. Model 2 is adjusted for sex, year of birth, and educational attainment. Model 3 is adjusted for sex, year of birth, educational attainment, VTE in sibling, Human immunodeficiency virus (HIV) disease, Chronic viral hepatitis B, Chronic viral hepatitis C, Other thrombophilia, Activated protein C resistance [factor V Leiden mutation], Deficiency antithrombin, Deficiency protein C, Deficiency protein S. Reference for multimorbidity was no or one disease (score=0 or 1). Reference for multimorbidity severity (0-≥5) was no disease (score=0). | | | | | | | | | |

| **Supplementary Table 20.** Landmark 2 stratified for Activated protein C resistance [factor V Leiden mutation] (D68.5A). Subdistribution Hazard Ratios (subHR), incidence rates, and incidence rate ratios with 95% confidence intervals (CI), for venous thromboembolism (VTE) according to multimorbidity score ≥2 and Activated protein C resistance [factor V Leiden mutation] (D68.5A) for Landmark 2. | | | | | | | | | |
| --- | --- | --- | --- | --- | --- | --- | --- | --- | --- |
| **Multimorbidity score** | **Person years, No.** | **Cases, No./persons at risk, /No** | **Incidence rate, cases/1000 person-years** | **Incidence rate ratio (95%CI)**  **Model 1 Model 2 Model 3** | | | **HR (95%CI)**  **Model 1 Model 2 Model 3** | | |
| D68.5A <2 | 5388 | 26/1374 | 4.83 (3.29-7.09) | 1[Reference] | 1[Reference] | 1[Reference] | 1[Reference] | 1[Reference] | 1[Reference] |
| D68.5A ≥2 | 4180 | 37/1084 | 8.85 (6.41-12.21) | 1.83 (1.11-3.03) | 1.46 (0.86-2.49) | 1.49 (0.88-2.52) | 1.82 (1.10-3.00) | 1.46 (0.84-2.52) | 1.47 (0.87-2.51) |
| Without Score <2 | 23409713 | 24290/5985719 | 1.04 (1.03-1.05) | 1[Reference] | 1[Reference] | 1[Reference] | 1[Reference] | 1[Reference] | 1[Reference] |
| Without Score ≥2 | 8313160 | 30858/2182152 | 3.71 (3.67-3.76) | 3.58 (3.52-3.64) | 1.40 (1.37-1.42) | 1.39 (1.36-1.41) | 3.50 (3.44-3.56) | 1.37 (1.35-1.40) | 1.36 (1.34-1.39) |
| **Activated protein C resistance [factor V Leiden mutation] (D68.5A)** | | | | | | | | | |
| Score 0 | 3139 | 13/800 | 4.14 (2.40-7.13) | 1[Reference] | 1[Reference] | 1[Reference] | 1[Reference] | 1[Reference] | 1[Reference] |
| Score 1 | 2248 | 13/574 | 5.78 (3.36-9.96) | 1.40 (0.65-3.01) | 1.49 (0.69-3.21) | 1.52 (0.70-3.29) | 1.40 (0.65-3.02) | 1.50 (0.69-3.27) | 1.54 (0.70-3.39) |
| Score 2 | 1480 | 10/379 | 6.75 (3.63-12.55) | 1.63 (0.72-3.72) | 1.57 (0.68-3.61) | 1.65 (0.72-3.80) | 1.63 (0.72-3.73) | 1.59 (0.67-3.75) | 1.67 (0.71-3.90) |
| Score 3 | 1032 | 12/267 | 11.63 (6.60-20.48) | 2.81 (1.28-6.16) | 2.65 (1.20-5.87) | 2.72 (1.23-6.04) | 2.80 (1.38-6.12) | 2.64 (1.20-5.80) | 2.69 (1.23-5.91) |
| Score 4 | 678 | 3/174 | 4.42 (1.43-13.72) | 1.07 (0.30-3.75) | 0.89 (0.25-3.18) | 0.87 (0.24-3.10) | 1.06 (0.30-3.71) | 0.88 (0.24-3.17) | 0.86 (0.24-3.15) |
| Score ≥5 | 990 | 12/264 | 12.12 (6.88-21.34) | 2.93 (1.34-6.41) | 1.68 (0.73-3.90) | 1.73 (0.75-3.98) | 2.85 (1.30-6.24) | 1.65 (0.67-4.07) | 1.69 (0.70-4.05) |
| **Without** | | | | | | | | | |
| Score 0 | 16928626 | 14701/433734 | 0.87 (0.86-0.88) | 1[Reference] | 1[Reference] | 1[Reference] | 1[Reference] | 1[Reference] | 1[Reference] |
| Score 1 | 6481087 | 9583/1648377 | 1.48 (1.45-1.51) | 1.70 (1.66-1.75) | 1.22 (1.19-1.25) | 1.19 (1.16-1.22) | 1.72 (1.67-1.76) | 1.23 (1.20-1.27) | 1.21 (1.17-1.24) |
| Score 2 | 3475153 | 8412/890984 | 2.42 (2.37-2.47) | 2.79 (2.71-2.86) | 1.36 (1.33-1.40) | 1.34 (1.30-1.37) | 2.79 (2.72-2.87) | 1.39 (1.35-1.43) | 1.35 (1.32-1.39) |
| Score 3 | 2009249 | 6900/521852 | 3.43 (3.36-3.52) | 3.96 (3.84-4.07) | 1.47 (1.43-1.51) | 1.45 (1.41-1.49) | 3.92 (3.81-4.03) | 1.48 (1.44-1.53) | 1.46 (1.41-1.50) |
| Score 4 | 1199403 | 5166/316618 | 4.31 (4.19-4.44) | 4.95(4.80-5.11) | 1.52 (1.47-1.57) | 1.50 (1.46-1.55) | 4.84 (4.69-5.00) | 1.51 (1.47-1.57) | 1.49 (1.44-1.54) |
| Score ≥5 | 1629354 | 10380/452698 | 6.37 (6.25-6.50) | 7.33 (7.15-7.52) | 1.74 (1.69-1.79) | 1.73 (1.68-1.78) | 6.83 (6.66-7.01) | 1.63 (1.58-1.68) | 1.62 (1.57-1.66) |
| Model 1 is a crude model. Model 2 is adjusted for sex, year of birth, and educational attainment. Model 3 is adjusted for sex, year of birth, educational attainment, VTE in sibling, Human immunodeficiency virus (HIV) disease, Chronic viral hepatitis B, Chronic viral hepatitis C, Other thrombophilia, Deficiency antithrombin, Deficiency protein C, Deficiency protein S, Prothrombin gene mutation. Reference for multimorbidity was no or one disease (score=0 or 1). Reference for multimorbidity severity (0-≥5) was no disease (score=0). | | | | | | | | | |

| **Supplementary Table 21.** Landmark 2 stratified for Deficiency antithrombin (D68.5B). Subdistribution Hazard Ratios (subHR), incidence rates, and incidence rate ratios with 95% confidence intervals (CI), for venous thromboembolism (VTE) according to multimorbidity score ≥2 and Deficiency antithrombin (D68.5B) for Landmark 2. | | | | | | | | | |
| --- | --- | --- | --- | --- | --- | --- | --- | --- | --- |
| **Multimorbidity score** | **Person years, No.** | **Cases, No./persons at risk, /No** | **Incidence rate, cases/1000 person-years** | **Incidence rate ratio (95%CI)**  **Model 1 Model 2 Model 3** | | | **subHR (95%CI)**  **Model 1 Model 2 Model 3** | | |
| D68.5B <2 | 168 | 2/44 | 11.89 (3.97-47.54) | 1[Reference] | 1[Reference] | 1[Reference] | 1[Reference] | 1[Reference] | 1[Reference] |
| D68.5B ≥2 | 143 | 3/39 | 21.02 (6.78-65.2) | 1.77 (0.30-10.58) | 1.66 (0.25-11.07) | 1.58 (0.21-11.68) | 1.66 (0.28-9.97) | 1.57 (0.26-9.62) | 1.52 (0.26-9.03) |
| Without Score <2 | 23414933 | 24314/5987049 | 1.04 (1.03-1.05) | 1[Reference] | 1[Reference] | 1[Reference] | 1[Reference] | 1[Reference] | 1[Reference] |
| Without Score ≥2 | 8317198 | 30892/2183197 | 3.71 (3.67-3.76) | 3.58 (3.52-3.64) | 1.40 (1.37-1.42) | 1.39 (1.36-1.41) | 3.50 (3.44-3.56) | 1.37 (1.35-1.40) | 1.36 (1.34-1.39) |
| **Deficiency antithrombin (D68.5B)** | | | | | | | | | |
| Score 0 | 100 | 0/25 | - | 1[Reference] | 1[Reference] | 1[Reference] | 1[Reference] | 1[Reference] | 1[Reference] |
| Score 1 | 68 | 2/19 | 29.32 (7.33-117.25) | - | - | - | - | - | - |
| Score 2 | 44 | 0/11 | - | - | - | - | 1 (0.54-1.87) | 1.07 (0.46-2.51) | - |
| Score 3 | 33 | 1/9 | 30.70 (3.32-217.96) | - | - | - | - | - | - |
| Score 4 | 16 | 1/6 | 62.11 (8.75-440.93) | - | - | - | - | - | - |
| Score ≥5 | 50 | 1/13 | 19.98 (2.81-141.84) | - | - | - | - | .- | - |
| **Without** | | | | | | | | | |
| Score 0 | 16931666 | 14720/4338117 | 0.87 (0.86-0.88) | 1[Reference] | 1[Reference] | 1[Reference] | 1[Reference] | 1[Reference] | 1[Reference] |
| Score 1 | 6483267 | 9594/1648932 | 1.48 (1.45-1.51) | 1.70 (1.66-1.75) | 1.22 (1.19-1.25) | 1.19 (1.16-1.22) | 1.72 (1.67-1.76) | 1.23 (1.20-1.27) | 1.21 (1.17-1.24) |
| Score 2 | 3476590 | 8422/891352 | 2.42 (2.37-2.47) | 2.79 (2.71-2.86) | 1.36 (1.33-1.40) | 1.34 (1.30-1.37) | 2.79 (2.72-2.87) | 1.39 (1.35-1.43) | 1.35 (1.32-1.39) |
| Score 3 | 2010248 | 6911/522110 | 3.43 (3.36-3.52) | 3.96 (3.84-4.07) | 1.47 (1.43-1.51) | 1.45 (1.41-1.49) | 3.92 (3.81-4.03) | 1.49 (1.44-1.53) | 1.46 (1.41-1.50) |
| Score 4 | 1200065 | 5168/316786 | 4.31 (4.19-4.44) | 4.95(4.80-5.11) | 1.52 (1.47-1.57) | 1.50 (1.46-1.55) | 4.84 (4.69-5.00) | 1.52 (1.47-1.57) | 1.49 (1.44-1.54) |
| Score ≥5 | 1630294 | 10391/452949 | 6.37 (6.25-6.50) | 7.33 (7.15-7.52) | 1.74 (1.69-1.79) | 1.73 (1.68-1.78) | 6.83 (6.66-7.01) | 1.63 (1.58-1.68) | 1.62 (1.57-1.66) |
| Model 1 is a crude model. Model 2 is adjusted for sex, year of birth, and educational attainment. Model 3 is adjusted for sex, year of birth, educational attainment, VTE in sibling, Human immunodeficiency virus (HIV) disease, Chronic viral hepatitis B, Chronic viral hepatitis C, Other thrombophilia, Activated protein C resistance [factor V Leiden mutation], Deficiency protein C, Deficiency protein S, Prothrombin gene mutation. Reference for multimorbidity was no or one disease (score=0 or 1). Reference for multimorbidity severity (0-≥5) was no disease (score=0). | | | | | | | | | |

| **Supplementary Table 22.** Landmark 2 stratified for Deficiency protein C (D68.5C). Subdistribution Hazard Ratios (subHR), incidence rates, and incidence rate ratios with 95% confidence intervals (CI), for venous thromboembolism (VTE) according to multimorbidity score ≥2 and Deficiency protein C (D68.5C) for Landmark 2. | | | | | | | | | |
| --- | --- | --- | --- | --- | --- | --- | --- | --- | --- |
| **Multimorbidity score** | **Person years, No.** | **Cases, No./persons at risk, /No** | **Incidence rate, cases/1000 person-years** | **Incidence rate ratio (95%CI)**  **Model 1 Model 2 Model 3** | | | **subHR (95%CI)**  **Model 1 Model 2 Model 3** | | |
| D68.5C <2 | 400 | 3/102 | 7.49 (2.42-23.23) | 1[Reference] | 1[Reference] | 1[Reference] | 1[Reference] | 1[Reference] | 1[Reference] |
| D68.5C ≥2 | 271 | 2/69 | 7.37 (1.84-29.48) | 0.98 (0.16-5.89) | 0.53 (0.068-4.09) | 0.47 (0.049-4.63) | 0.99 (0.17-5.91) | 0.53 (0.060-4.66) | 0.48 (0.040-5.71) |
| Without Score <2 | 23414701 | 24313/5986991 | 1.04 (1.03-1.05) | 1[Reference] | 1[Reference] | 1[Reference] | 1[Reference] | 1[Reference] | 1[Reference] |
| Without Score ≥2 | 8317069 | 30893/2183167 | 3.71 (3.67-3.76) | 3.58 (3.52-3.64) | 1.40 (1.37-1.42) | 1.39 (1.36-1.41) | 3.50 (3.44-3.56) | 1.37 (1.35-1.40) | 1.36 (1.34-1.39) |
| **Deficiency protein C (D68.5C)** | | | | | | | | | |
| Score 0 | 252 | 3/65 | 11.89 (3.83-36.85) | 1[Reference] | 1[Reference] | 1[Reference] | 1[Reference] | 1[Reference] | 1[Reference] |
| Score 1 | 148 | 0/37 | - | - | - | - | - | - | - |
| Score 2 | 113 | 1/29 | 8.88 (1.25-63.06) | 0.75(0.078-7.19) | 0.48 (0.041-5.64) | 0.43 (0.035-5.36) | 0.76 (0.076-7.37) | 0.49 (0.071-3.33) | 0.43 (0.037-5.05) |
| Score 3 | 80 | 0/20 | - | - | - | - | - | - | - |
| Score 4 | 28 | 0/7 | - | - | - | - | - | - | - |
| Score ≥5 | 51 | 1/13 | 19.72 (2. 87-140.04) | 1.66 (0.17-15.96) | 0.33 (0.099-11.06) | 0.15 (0.0027-8.39) | 1.67 (018-15.22) | .57 (0.015-21.92) | 0.33 (0.0049-225.52) |
| **Without** | | | | | | | | | |
| Score 0 | 16931513 | 14717/4338077 | 0.87 (0.86-0.88) | 1[Reference] | 1[Reference] | 1[Reference] | 1[Reference] | 1[Reference] | 1[Reference] |
| Score 1 | 6483187 | 9596/1648914 | 1.48 (1.45-1.51) | 1.70 (1.66-1.75) | 1.22 (1.19-1.25) | 1.19 (1.16-1.22) | 1.72 (1.67-1.76) | 1.24 (1.20-1.27) | 1.21 (1.18-1.24) |
| Score 2 | 3476251 | 8421/891334 | 2.42 (2.37-2.47) | 2.79 (2.71-2.86) | 1.36 (1.33-1.40) | 1.34 (1.30-1.37) | 2.79 (2.72-2.87) | 1.39 (1.35-1.43) | 1.35 (1.32-1.39) |
| Score 3 | 2010200 | 6912/522099 | 3.43 (3.36-3.52) | 3.96 (3.84-4.07) | 1.47 (1.43-1.51) | 1.45 (1.41-1.49) | 3.92 (3.81-4.04) | 1.49 (1.44-1.53) | 1.46 (1.41-1.50) |
| Score 4 | 1200053 | 5169/316785 | 4.31 (4.19-4.44) | 4.95(4.80-5.11) | 1.52 (1.47-1.57) | 1.50 (1.46-1.55) | 4.84 (4.69-5.00) | 1.52 (1.47-1.57) | 1.49 (1.44-1.54) |
| Score ≥5 | 1630293 | 10391/452949 | 6.37 (6.25-6.50) | 7.33 (7.15-7.52) | 1.74 (1.69-1.79) | 1.73 (1.68-1.78) | 6.83 (6.66-7.01) | 1.63 (1.58-1.68) | 1.62 (1.57-1.67) |
| Model 1 is a crude model. Model 2 is adjusted for sex, year of birth, and educational attainment. Model 3 is adjusted for sex, year of birth, educational attainment, VTE in sibling, Human immunodeficiency virus (HIV) disease, Chronic viral hepatitis B, Chronic viral hepatitis C, Other thrombophilia, Activated protein C resistance [factor V Leiden mutation], Deficiency antithrombin, Deficiency protein S, Prothrombin gene mutation. Reference for multimorbidity was no or one disease (score=0 or 1). Reference for multimorbidity severity (0-≥5) was no disease (score=0). | | | | | | | | | |

| **Supplementary Table 23.** Landmark 2 stratified for Deficiency protein S (D68.5D). Subdistribution Hazard Ratios (subHR), incidence rates, and incidence rate ratios with 95% confidence intervals (CI), for venous thromboembolism (VTE) according to multimorbidity score ≥2 and Deficiency protein S (D68.5D) for Landmark 2. | | | | | | | | | |
| --- | --- | --- | --- | --- | --- | --- | --- | --- | --- |
| **Multimorbidity score** | **Person years, No.** | **Cases, No./persons at risk, /No** | **Incidence rate, cases/1000 person-years** | **Incidence rate ratio (95%CI)**  **Model 1 Model 2 Model 3** | | | **subHR (95%CI)**  **Model 1 Model 2 Model 3** | | |
| D68.5D <2 | 290 | 6/77 | 20.67 (9.29-46.04) | 1[Reference] | 1[Reference] | 1[Reference] | 1[Reference] | 1[Reference] | 1[Reference] |
| D68.5D ≥2 | 293 | 3/76 | 10.24 (3.30-31.76) | 0.50 (0.12-1.98) | 0.25 (0.042-1.51) | 0.094 (0.0090-0.99) | 0.50 (0.12-1.97)) | 0.26 (0.043-1.65) | 0.14 (0.086-2.37) |
| Without Score <2 | 23414811 | 24310/5987016 | 1.04 (1.03-1.05) | 1[Reference] | 1[Reference] | 1[Reference] | 1[Reference] | 1[Reference] | 1[Reference] |
| Without Score ≥2 | 8317048 | 30892/2183160 | 3.71 (3.67-3.76) | 3.58 (3.52-3.64) | 1.40 (1.37-1.43) | 1.39 (1.36-1.41) | 3.50 (3.45-3.56) | 1.37 (1.35-1.40) | 1.36 (1.34-1.39) |
| **Deficiency protein S (D68.5D)** | | | | | | | | | |
| Score 0 | 143 | 2/37 | 14.03 (3.51-56.08) | 1[Reference] | 1[Reference] | 1[Reference] | 1[Reference] | 1[Reference] | 1[Reference] |
| Score 1 | 147 | 4/40 | 27.12 (10.18-72.28) | 1.93 (0.35-10.56) | 1.80 (0.29-9.98) | 2.22 (0.35-14.17) | 1.92 (0.36-10.3) | 1.69 (0.29-9.79) | 2.22 (0.32-15.67) |
| Score 2 | 125 | 1/32 | 7.99 (1.13-56.72) | 0.57 (0.052-6.28) | 0.34 (0.019-6.10) | 0.059 (0.0017-2.12) | 0.57 (0.053-6.25) | 0.37 (0.033-4.12) | 0.14 (0.0036-5.29) |
| Score 3 | 60 | 0/15 | - | - | - | - | - | - | - |
| Score 4 | 40 | 1/10 | 25.24 (3.56-179.18) | 1.80 (0.16-19.84) | 1.03 (0.089-11.93) | 1.25 (0.099-15.90) | 1.84 (0.18-18.80) | 1.10 (0.11-11.16) | 1.45 (0.097-21.48) |
| Score ≥5 | 68 | 1/19 | 14.68 (2.07-104.20) | 1.05 (0.095-11.54) | 0.19 (0.056-6.52) | 0.15 (0.0027-8.39) | 1.0 (0.89-11.20) | 0.019 (0.012-2.90) | 0.18 (0.0035-9.54) |
| **Without** | | | | | | | | | |
| Score 0 | 16931623 | 14718/4338105 | 0.87 (0.85-0.88) | 1[Reference] | 1[Reference] | 1[Reference] | 1[Reference] | 1[Reference] | 1[Reference] |
| Score 1 | 6483188 | 9592/1648911 | 1.48 (1.45-1.51) | 1.70 (1.66-1.75) | 1.22 (1.19-1.25) | 1.19 (1.16-1.22) | 1.72 (1.67-1.76) | 1.23 (1.20-1.27) | 1.21 (1.17-1.24) |
| Score 2 | 3476509 | 8421/891331 | 2.42 (2.37-2.47) | 2.79 (2.71-2.86) | 1.36 (1.33-1.40) | 1.34 (1.30-1.37) | 2.79 (2.72-2.87) | 1.39 (1.35-1.43) | 1.35 (1.32-1.39) |
| Score 3 | 2010220 | 6912/522104 | 3.33 (3.34-3.52) | 3.96 (3.84-4.07) | 1.47 (1.43-1.51) | 1.45 (1.41-1.49) | 3.92 (3.81-4.04) | 1.48 (1.44-1.53) | 1.46 (1.41-1.50) |
| Score 4 | 1200041 | 5168/316782 | 4.31 (4.19-4.44) | 4.95(4.80-5.11) | 1.52 (1.47-1.57) | 1.50 (1.46-1.55) | 4.84 (4.69-5.00) | 1.52 (1.46-1.57) | 1.49 (1.44-1.54) |
| Score ≥5 | 1630276 | 10391/452943 | 6.37 (6.25-6.50) | 7.33 (7.15-7.52) | 1.74 (1.69-1.79) | 1.73 (1.68-1.78) | 6.83 (6.66-7.01) | 1.63 (1.58-1.68) | 1.62 (1.57-1.66) |
| Model 1 is a crude model. Model 2 is adjusted for sex, year of birth, and educational attainment. Model 3 is adjusted for sex, year of birth, educational attainment, VTE in sibling, Human immunodeficiency virus (HIV) disease, Chronic viral hepatitis B, Chronic viral hepatitis C, Other thrombophilia, Activated protein C resistance [factor V Leiden mutation], Deficiency antithrombin, Deficiency protein C, Prothrombin gene mutation. Reference for multimorbidity was no or one disease (score=0 or 1). Reference for multimorbidity severity (0-≥5) was no disease (score=0). | | | | | | | | | |

| **Supplementary Table 24.** Landmark 2 stratified for other thrombophilia (D68.6). Subdistribution Hazard Ratios (subHR), incidence rates, and incidence rate ratios with 95% confidence intervals (CI), for venous thromboembolism (VTE) according to multimorbidity score ≥2 and Other thrombophilia (D68.6) for Landmark 2. | | | | | | | | | |
| --- | --- | --- | --- | --- | --- | --- | --- | --- | --- |
| **Multimorbidity score** | **Person years, No.** | **Cases, No./persons at risk, /No** | **Incidence rate, cases/1000 person-years** | **Incidence rate ratio (95%CI)**  **Model 1 Model 2 Model 3** | | | **subHR (95%CI)**  **Model 1 Model 2 Model 3** | | |
| D68.6 <2 | 673 | 5/175 | 7.4 (3.1-17.86) | 1[Reference] | 1[Reference] | 1[Reference] | 1[Reference] | 1[Reference] | 1[Reference] |
| D68.6 ≥2 | 933 | 6/249 | 6.43 (2.89-14.3) | 0.86 (0.26-2.83) | 0.52 (0.14-1.92) | 0.42 (0.11-1.70) | 0.85 (0.26-2.76) | 0.54 (0.12-2.38) | 0.45 (0.09-2.33) |
| Without Score <2 | 23414428 | 24311/5986918 | 1.04 (1.03-1.05) | 1[Reference] | 1[Reference] | 1[Reference] | 1[Reference] | 1[Reference] | 1[Reference] |
| Without Score ≥2 | 8316407 | 30889/2182987 | 3.71 (3.67-3.76) | 3.58 (3.52-3.64) | 1.40 (1.37-1.43) | 1.39 (1.36-1.41) | 3.50 (3.44-3.56) | 1.37 (1.35-1.40) | 1.36 (1.34-1.39) |
| **Other thrombophilia (D68.6)** | | | | | | | | | |
| Score 0 | 294 | 3/79 | 10.2 (3.29—31.62) | 1[Reference] | 1[Reference] | 1[Reference] | 1[Reference] | 1[Reference] | 1[Reference] |
| Score 1 | 379 | 2/96 | 5.28 (1.32-21.23) | 0.52 (0.087-3.10) | 0.40 (0.066-2.37) | 0.39 (0.064-2.32) | 0.55 (0.092-3.25) | 0.48 (0.077-3.04) | 0.49 (0.076-3.15) |
| Score 2 | 259 | 2/67 | 7.73 (1.93-30.89) | 0.76 (0.13-4.53) | 0.53 (0.082-3.36) | 0.38 (0.054-2.64) | 0.79 (0.32-4.72) | 0.62 (0.11-3.65) | 0.49 (0.068-3.57) |
| Score 3 | 213 | 2/56 | 9.37 (2.34-37.48) | 0.92 (0.15-5.50) | 0.60 (0.096-3.69) | 0.48 (0.074-3.08) | 0.94 (0.16-5.51) | 0.67 (0.11-4.23) | 0.52 (0.095-2.78) |
| Score 4 | 132 | 1/35 | 7.59 (1.07-53.86) | 0.74 (0.078-7.15) | 0.40 (0.037-4.30) | 0.33 (0.029-3.69) | 0.76 (0.078-7.30) | 0.47 (0.32-6.99) | 0.41 (0.022-7.60) |
| Score ≥5 | 329 | 1/91 | 3.04 (0.43-21.56) | 0.30 (0.031-2.86) | 0.10 (0.099-1.07) | 0.075 (0.0065-0.87) | 0.29 (0.030-2.79) | 0.13 (0.013-1.23) | 0.11 (0.0082-1.37) |
| **Without** | | | | | | | | | |
| Score 0 | 16931471 | 14717/4338063 | 0.87 (0.86-0.88) | 1[Reference] | 1[Reference] | 1[Reference] | 1[Reference] | 1[Reference] | 1[Reference] |
| Score 1 | 6482957 | 9594/1648855 | 1.48 (1.45-1.51) | 1.70 (1.66-1.75) | 1.22 (1.19-1.25) | 1.19 (1.16-1.22) | 1.72 (1.67-1.76) | 1.24 (1.20-1.28) | 1.21 (1.17-1.24) |
| Score 2 | 3476375 | 8420/891296 | 2.42 (2.37-2.47) | 2.79 (2.71-2.86) | 1.36 (1.33-1.40) | 1.34 (1.30-1.37) | 2.79 (2.72-2.87) | 1.39 (1.35-1.43) | 1.35 (1.32-1.39) |
| Score 3 | 2010067 | 6910/522063 | 3.44 (3.36-3.52) | 3.95 (3.84-4.07) | 1.47 (1.43-1.51) | 1.45 (1.41-1.49) | 3.92 (3.91-4.03) | 1.48 (1.44-1.53) | 1.46 (1.41-1.50) |
| Score 4 | 1199950 | 5168/316757 | 4.31 (4.19-4.43) | 4.95 (4.80-5.11) | 1.51 (1.47-1.57) | 1.50 (1.46-1.55) | 4.84 (4.69-5.00) | 1.52 (1.47-1.57) | 1.49 (1.44-1.54) |
| Score ≥5 | 1630015 | 10391/452871 | 6.37 (6.25-6.50) | 7.33 (7.15-7.52) | 1.74 (1.60-1.79) | 1.73 (1.68-1.79) | 6.83 (6.67-7.01) | 1.63 (1.58-1.68) | 1.62 (1.57-1.67) |
| Model 1 is a crude model. Model 2 is adjusted for sex, year of birth, and educational attainment. Model 3 is adjusted for sex, year of birth, educational attainment, VTE in sibling, Human immunodeficiency virus (HIV) disease, Chronic viral hepatitis B, Chronic viral hepatitis C, Activated protein C resistance [factor V Leiden mutation], Deficiency antithrombin, Deficiency protein C, Deficiency protein S, Prothrombin gene mutation. Reference for multimorbidity was no or one disease (score=0 or 1). Reference for multimorbidity severity (0-≥5) was no disease (score=0). | | | | | | | | | |

| **Supplementary Table 25**. Subdistribution Hazard Ratios (subHR), incidence rates, and incidence rate ratios for multimorbidity for Landmark 2 based on 45 diseases with model 4 including adjustments for varicose veins. | | | | | | | | | | | |
| --- | --- | --- | --- | --- | --- | --- | --- | --- | --- | --- | --- |
| **Multimorbidity score** | **Person years, No.** | **Cases, No./persons at risk, /No** | **Incidence rate, cases/1000 person-years** | **Incidence rate ratio (95%CI)**  **Model 1 Model 2 Model3** | | | **subHR (95%CI)**  **Model 1 Model 2 Model 3 Model 4** | | | |  |
| Score <2 | 23415101 | 24316/5987093 | 1.04 (1.03-1.05) | 1[Reference] | 1[Reference] | 1[Reference] | 1[Reference] | 1[Reference] | 1[Reference] | 1[Reference] |  |
| Score ≥2 | 8317341 | 30896/2183236 | 3.71 (3.67-3.76) | 3.58 (3.52-3.64) | 1.40 (1.37-1.42) | 1.39 (1.36-1.41) | 3.50 (3.44-3.56) | 1.37 (1.35-1.40) | 1.36 (1.34-1.39) | 1.35 (1.32-1.37) |  |
| Score 0 | 16931766 | 14720/4338142 | 0.87 (0.86-0.88) | 1[Reference] | 1[Reference] | 1[Reference] | 1[Reference] | 1[Reference] | 1[Reference] | 1[Reference] |  |
| Score 1 | 6483335 | 9596/1648951 | 1.48 (1.45-1.51) | 1.70 (1.66-1.75) | 1.22 (1.19-1.25) | 1.19 (1.16-1.22) | 1.72 (1.67-1.76) | 1.24 (1.20-1.27) | 1.21 (1.18-1.24) | 1.20 (1.17-1.23) |  |
| Score 2 | 3476634 | 8422/891363 | 2.42 (2.37-2.47) | 2.79 (2.71-2.86) | 1.36 (1.33-1.40) | 1.34 (1.30-1.37) | 2.79 (2.72-2.87) | 1.39 (1.35-1.43) | 1.36 (1.32-1.39) | 1.35 (1.31-1.38) |  |
| Score 3 | 2010281 | 6912/522119 | 3.44 (3.36-3.52) | 3.95 (3.84-4.07) | 1.47 (1.43-1.51) | 1.45 (1.41-1.49) | 3.92 (3.81-4.04) | 1.49 (1.44-1.53) | 1.46 (1.41-1.50) | 1.44 (1.40-1.48) |  |
| Score 4 | 1200082 | 5169/316792 | 4.31 (4.19-4.43) | 4.95 (4.80-5.11) | 1.52 (1.47-1.57) | 1.50 (1.46-1.55) | 4.84 (4.69-5.00) | 1.52 (1.47-1.57) | 1.49 (1.44-1.54) | 1.46 (1.42-1.48) |  |
| Score ≥5 | 1630344 | 10392/452962 | 6.37 (6.25-6.50) | 7.33 (7.15-7.52) | 1.74 (1.69-1.79) | 1.73 (1.68-1.78) | 6.83 (6.66-7.01) | 1.63 (1.58-1.68) | 1.62 (1.57-1.67) | 1.58 (1.53-1.62) |  |
| Model 1 is a crude model. Model 2 is adjusted for sex, year of birth, and educational attainment. Model 3 is adjusted for sex, year of birth, educational attainment, country of birth, VTE in sibling, Human immunodeficiency virus (HIV) disease, Chronic viral hepatitis B, Chronic viral hepatitis C, Other thrombophilia, Activated protein C resistance [factor V Leiden mutation], Deficiency antithrombin, Deficiency protein C, Deficiency protein S, Prothrombin gene mutation. Model 4 is adjusted for sex, year of birth, educational attainment, country of birth, VTE in sibling, Human immunodeficiency virus (HIV) disease, Chronic viral hepatitis B, Chronic viral hepatitis C, Other thrombophilia, Activated protein C resistance [factor V Leiden mutation], Deficiency antithrombin, Deficiency protein C, Deficiency protein S, Prothrombin gene mutation, Varicose veins.. Reference for multimorbidity was no or one disease (score=0 or 1). Reference for multimorbidity severity (0-≥5) was no disease (score=0).CI=Confidence interval | | | | | | | | | | | |

| **Supplementary Table 26**. Subdistribution Hazard Ratios (subHR), incidence rates, and incidence rate ratios for multimorbidity for Landmark 2 total population before exclusion criteria based on 45 diseases. | | | | | | | | | | |
| --- | --- | --- | --- | --- | --- | --- | --- | --- | --- | --- |
| **Multimorbidity score** | **Person years, No.** | **Cases, No./persons at risk, /No** | **Incidence rate, cases/1000 person-years** | **Incidence rate ratio (95%CI)**  **Model 1 Model 2 Model3** | | | **subHR (95%CI)**  **Model 1 Model 2 Model 3 Model 4** | | | |
| Score <2 | 26074552 | 28408/6664839 | 1.09 (1.08-1.10) | 1[Reference] | 1[Reference] | 1[Reference] | 1[Reference] | 1[Reference] | 1[Reference] | 1[Reference] |
| Score ≥2 | 10851832 | 41167/2860401 | 3.79 (3.76-3.83) | 3.48 (3.43-3.53) | 1.36 (1.34-1.39) | 1.36 (1.33-1.38) | 3.39 (3.34-3.45) | 1.34 (1.32-1.37) | 1.31 (1.31-1.36) | 1.36 (1.34-1.39) |
| Score 0 | 18549418 | 17066/4750806 | 0.92 (0.91-0.93) | 1[Reference] | 1[Reference] | 1[Reference] | 1[Reference] | 1[Reference] | 1[Reference] | 1[Reference] |
| Score 1 | 7525134 | 11342/1914033 | 1.51 (1.48-1.53) | 1.64 (1.60-1.68) | 1.20 (1.17-1.23) | 1.18 (1.15-1.21) | 1.65 (1.61-1.69) | 1.22 (1.19-1.25) | 1.20 (1.17-1.22) | 1.19 (1.16-1.22) |
| Score 2 | 4233754 | 10196/1085515 | 2.41 (2.36-2.46) | 2.62 (2.55-2.68) | 1.33 (1.30-1.37) | 1.31 (1.27-1.34) | 2.62 (2.56-2.69) | 1.36 (1.32-1.39) | 1.33 (1.30-1.36) | 1.33 (1.30-1.36) |
| Score 3 | 2564108 | 8761/666182 | 3.42(3.35-3.49) | 3.71 (3.62-3.81) | 1.44 (1.40-1.48) | 1.42 (1.38-1.45) | 3.68 (3.59-3.78) | 1.46 (1.42-1.50) | 1.43 (1.39-1.47) | 1.44 (1.40-1.48) |
| Score 4 | 1599860 | 6836/422506 | 4.27 (4.17-4.43) | 4.64 (4.52-4.78) | 1.48 (1.44-1.53) | 1.46 (1.42-1.51) | 4.53 (4.41-4.66) | 1.48 (1.44-1.53) | 1.46 (1.42-1.51) | 1.49 (1.44-1.53) |
| Score ≥5 | 2454110 | 15374/686198 | 6.26 (6.17-6.36) | 6.81 (6.66-6.96) | 1.64 (1.61-1.68) | 1.64 (1.60-1.68) | 6.30 (6.16-6.44) | 1.55 (1.51-1.59) | 1.54 (1.50-1.58) | 1.62 (1.58-1.66) |
| Model 1 is a crude model. Model 2 is adjusted for sex, year of birth, and educational attainment. Model 3 is adjusted for sex, year of birth, educational attainment, country of birth, VTE in sibling, Human immunodeficiency virus (HIV) disease, Chronic viral hepatitis B, Chronic viral hepatitis C, Other thrombophilia, Activated protein C resistance [factor V Leiden mutation], Deficiency antithrombin, Deficiency protein C, Deficiency protein S, Prothrombin gene mutation. Model 4 is adjusted for sex, year of birth, educational attainment, country of birth, VTE in sibling, Human immunodeficiency virus (HIV) disease, Chronic viral hepatitis B, Chronic viral hepatitis C, Other thrombophilia, Activated protein C resistance [factor V Leiden mutation], Deficiency antithrombin, Deficiency protein C, Deficiency protein S, Prothrombin gene mutation, DOAC, Warfarin, oral contraceptives, hormonal replacement therapy Reference for multimorbidity was no or one disease (score=0 or 1). Reference for multimorbidity severity (0-≥5) was no disease (score=0).CI=Confidence interval | | | | | | | | | | |

| **Supplementary Table 27.** Descriptive findings for study participants in Landmark 1 | | | | | | | | | |
| --- | --- | --- | --- | --- | --- | --- | --- | --- | --- |
| **Multimorbidity score** | | | | | | | | | |
|  | All | 0 | 1 | 2 | 3 | 4 | ≥ 5 | ≤ 1 | ≥ 2 |
| Unique individuals, n (%) | 8068489  (100) | 5751476  (71.28) | 1226438  (15.20) | 543005  (6.73) | 271871  (3.37) | 139976  (1.73) | 135723  (1.68) | 6977914  (86.48) | 1090575  (13.52) |
| Sex, Female,  n (%) | 3428881  (42.50) | 2337431  (68.17) | 544644  (15.88) | 264906  (7.73) | 139184  (4.06) | 72617  (2.12) | 70099  (2.04) | 2882075  (84.05) | 546806  (15.95) |
| Education (≥12 years), n (%) | 3208591  (39.77) | 2448453  (42.57) | 475483  (38.77) | 166458  (30.65) | 66857  (24.59) | 28867  (20.62) | 22473  (16.56) | 2923936  (41.90) | 284655  (26.10) |
| Age at end of study,  Median (IQR)  Range (min-max) | 45  (27-63)  (0.08-111) | 39  (24-57)  (0.08-111) | 51  (33-67)  (1-109) | 61  (46-75)  (1.8-109) | 69  (54-81)  (3-108) | 74  (60-84)  (6-109) | 79  (68-88)  (6-108) | 42  (25-59)  (0.08-111) | 68  (52-80)  (1.8-109) |
| Venous thromboembolism, n (%) | 90332  (1.12) | 45313  (0.79) | 16497  (1.35) | 11141  (2.05) | 7521  (2.77) | 4501  (3.22) | 5359  (3.95) | 61810  (0.89) | 28522  (2.62) |
| IQR=Interquartile Range | | | | | | | | | |

| **Supplementary Table 28**. Subdistributional Hazard Ratios (subHR), incidence rates, and incidence rate ratios with 95% confidence intervals (CI) for multimorbidity for Landmark 1 based on 45 diseases. | | | | | | | | | |
| --- | --- | --- | --- | --- | --- | --- | --- | --- | --- |
| **Multimorbidity score** | **Person years, No.** | **Cases, No./persons at risk,/No** | **Incidence rate, cases/1000 person-years** | **Incidence rate ratio (95%CI)**  **Model 1 Model 2 Model 3** | | | **subHR(95%CI)**  **Model 1 Model 2 Model 3** | | |
| Score <2 | 59729277 | 61810/6977914 | 1.03 (1.03-1.04) | 1[Reference] | 1[Reference] | 1[Reference] | 1[Reference] | 1[Reference] | 1[Reference] |
| Score ≥2 | 8554145 | 28522/1090575 | 3.33 (3.30-3.37) | 3.22 (3.18-3.27) | 1.32 (1.30-1.34) | 1.33 (1.31-1.35) | 2.98 (2.94-3.02) | 1.25 (1.23-1.27) | 1.25 (1.24-1.27) |
| Score 0 | 49235077 | 45313/5751476 | 0.92 (0.91-0.93) | 1[Reference] | 1[Reference] | 1[Reference] | 1[Reference] | 1[Reference] | 1[Reference] |
| Score 1 | 10494201 | 16497/1226438 | 1.57 (1.55-1.60) | 1.71 (1.68-1.74) | 1.18 (1.16-1.20) | 1.17 (1.15-1.19) | 1.71 (1.68-1.74) | 1.21 (1.19-1.23) | 1.19 (1.17-1.21) |
| Score 2 | 4486625 | 11141/543005 | 2.48 (2.44-2.53) | 2.70 (2.64-2.76) | 1.27 (1.25-1.30) | 1.27 (1.24-1.29) | 2.62 (2.57-2.68) | 1.28 (1.25-1.31) | 1.27 (1.24-1.30) |
| Score 3 | 2138216 | 7521/271871 | 3.52 (3.44-3.60) | 3.83 (3.73-3.92) | 1.39 (1.36-1.43) | 1.40 (1.36-1.43) | 3.55 (3.47-3.64) | 1.35 (1.32-1.38) | 1.35 (1.32-1.39) |
| Score 4 | 1039872 | 4501/139976 | 4.33 (4.20-4.45) | 4.70 (4.56-4.85) | 1.45 (1.40-1.49) | 1.46 (1.41-1.51) | 4.14 (4.02-4.27) | 1.33 (1.26-1.37) | 1.34 (1.30-1.38) |
| Score ≥5 | 889434 | 5359/135723 | 6.03 (5.87-6.19) | 6.55 (6.36-6.73) | 1.64 (1.59-1.69) | 1.67 (1.62-1.72) | 5.11 (4.97-5.26) | 1.34 (1.30-1.38) | 1.36 (1.32-1.40) |
| Model 1 is a crude model. Model 2 is adjusted for sex, year of birth, and educational attainment. Model 3 is adjusted for sex, year of birth, educational attainment, country of birth, VTE in sibling, Human immunodeficiency virus (HIV) disease, Chronic viral hepatitis B, Chronic viral hepatitis C, Other thrombophilia, Activated protein C resistance [factor V Leiden mutation], Deficiency antithrombin, Deficiency protein C, Deficiency protein S, Prothrombin gene mutation. Reference for multimorbidity was no or one disease (score=0 or 1). Reference for multimorbidity severity (0-≥5) was no disease (score=0). | | | | | | | | | |

| **Supplementary Table 29**. Subdistributional Hazard Ratios (subHR), incidence rates, and incidence rate ratios with 95% confidence intervals (CI) for multimorbidity for Landmark 1 males based on 45 diseases. | | | | | | | | | |
| --- | --- | --- | --- | --- | --- | --- | --- | --- | --- |
| **Multimorbidity score** | **Person years, No.** | **Cases, No./persons at risk, /No** | **Incidence rate, cases/1000 person-years** | **Incidence rate ratio (95%CI)**  **Model 1 Model 2 Model 3** | | | **subHR (95%CI)**  **Model 1 Model 2 Model 3** | | |
| Score <2 | 35099001 | 35652/4095839 | 1.02 (1.01-1.03) | 1[Reference] | 1[Reference] | 1[Reference] | 1[Reference] | 1[Reference] | 1[Reference] |
| Score ≥2 | 4239849 | 14355/543769 | 3.39 (3.33-3.44) | 3.33 (3.27-3.40) | 1.28 (1.26-1.31) | 1.30 (1.27-1.32) | 3.06 (3.01-3.12) | 1.20 (1.17-1.23) | 1.21 (1.18-1.24) |
| Score 0 | 29261635 | 26453/3414045 | 0.90 (0.89-0.92) | 1[Reference] | 1[Reference] | 1[Reference] | 1[Reference] | 1[Reference] | 1[Reference] |
| Score 1 | 5837367 | 9199/681794 | 1.58 (1.54-1.61) | 1.74 (1.70-1.79) | 1.19 (1.16-1.22) | 1.18 (1.15-1.21) | 1.75 (1.71-1.79) | 1.22 (1.19-1.25) | 1.20 (1.17-1.23) |
| Score 2 | 2292332 | 5830/278099 | 2.54 (2.48-2.61) | 2.81 (2.73-2.89) | 1.26 (1.23-1.30) | 1.26 (1.22-1.30) | 2.73 (2.65-2.80) | 1.27 (1.23-1.30) | 1.26 (1.22-1.30) |
| Score 3 | 1034629 | 3758/132687 | 3.63 (3.52-3.75) | 4.02 (3.88-4.16) | 1.35 (1.31-1.40) | 1.36 (1.31-1.41) | 3.70 (3.57-3.83) | 1.30 (1.25-1.34) | 1.30 (1.26-1.35) |
| Score 4 | 493316 | 2215/67359 | 4.49 (4.31-4.68) | 4.97 (4.76-5.19) | 1.40 (1.33-1.46) | 1.41 (1.35-1.47) | 4.31 (4.13-4.50) | 1.26 (1.20-1.32) | 1.28 (1.22-1.33) |
| Score ≥5 | 419571 | 2552/65624 | 6.08 (5.85-6.32) | 6.73 (6.46-7.01) | 1.56 (1.50-1.63) | 1.59 (1.53-1.66) | 5.12 (4.92-5.33) | 1.23 (1.17-1.28) | 1.26 (1.20-1.31) |
| Model 1 is a crude model. Model 2 is adjusted for year of birth, and educational attainment. Model 3 is adjusted for year of birth, educational attainment, country of birth, VTE in sibling, Human immunodeficiency virus (HIV) disease, Chronic viral hepatitis B, Chronic viral hepatitis C, Other thrombophilia, Activated protein C resistance [factor V Leiden mutation], Deficiency antithrombin, Deficiency protein C, Deficiency protein S, Prothrombin gene mutation. Reference for multimorbidity was no or one disease (score=0 or 1). Reference for multimorbidity severity (0-≥5) was no disease (score=0). | | | | | | | | | |

| **Supplementary Table 30**. Subdistributional Hazard Ratios (subHR), incidence rates, and incidence rate ratios with 95% confidence intervals (CI) for multimorbidity for Landmark 1 females based on 45 diseases. | | | | | | | | | |
| --- | --- | --- | --- | --- | --- | --- | --- | --- | --- |
| **Multimorbidity score** | **Person years, No.** | **Cases, No./persons at risk, /No** | **Incidence rate, cases/1000 person-years** | **Incidence rate ratio (95%CI)**  **Model 1 Model 2 Model3** | | | **subHR (95%CI)**  **Model 1 Model 2 Model 3** | | |
| Score <2 | 24630278 | 26158/288075 | 1.06 (1.05-1.08) | 1[Reference] | 1[Reference] | 1[Reference] | 1[Reference] | 1[Reference] | 1[Reference] |
| Score ≥2 | 4314297 | 14157/546806 | 3.33 (3.32-3.34) | 3.09 (3.03-3.16) | 1.36 (1.33-1.39) | 1.36 (1.33-1.39) | 2.88 (2.82-2.94) | 1.30 (1.27-1.33) | 1.30 (1.27-1.33) |
| Score 0 | 19973443 | 18860/2337431 | 0.94 (0.93-0.96) | 1[Reference] | 1[Reference] | 1[Reference] | 1[Reference] | 1[Reference] | 1[Reference] |
| Score 1 | 4856834 | 7298/544644 | 1.57 (1.53-1.60) | 1.66 (1.62-1.71) | 1.17 (1.14-1.20) | 1.15 (1.12-1.18) | 1.67 (1.62-1.71) | 1.19 (1.16-1.23) | 1.17 (1.14-1.21) |
| Score 2 | 2194293 | 5311/264906 | 2.43 (2.36-2.49) | 2.56 (2.49-2.64) | 1.27 (1.24-1.31) | 1.27 (1.23-1.31) | 2.50 (2.43-2.58) | 1.29 (1.25-1.33) | 1.28 (1.24-1.32) |
| Score 3 | 1103586 | 3763/139184 | 3.41 (3.30-3.52) | 3.61 (3.49-3.74) | 1.43 (1.38-1.49) | 1.43 (1.38-1.49) | 3.39 (3.27-3.51) | 1.40 (1.35-1.45) | 1.40 (1.35-1.45) |
| Score 4 | 546555 | 2286/72617 | 4.18 (4.01-4.36) | 4.43 (4.24-4.63) | 1.49 (1.43-1.56) | 1.51 (1.44-1.57) | 3.95 (3.79-4.13) | 1.39 (1.33-1.45) | 1.47 (1.41-1.53) |
| Score ≥5 | 469863 | 2807/7099 | 5.97 (5.76-6.20) | 6.33 (6.08-6.58) | 1.72 (1.65-1.79) | 1.75 (1.68-1.82) | 5.06 (4.87-5.27) | 1.45 (1.39-1.51) | 1.47 (1.41-1.53) |
| Model 1 is a crude model. Model 2 is adjusted for year of birth, and educational attainment. Model 3 is adjusted for year of birth, educational attainment, country of birth, VTE in sibling, Human immunodeficiency virus (HIV) disease, Chronic viral hepatitis B, Chronic viral hepatitis C, Other thrombophilia, Activated protein C resistance [factor V Leiden mutation], Deficiency antithrombin, Deficiency protein C, Deficiency protein S, Prothrombin gene mutation. Reference for multimorbidity was no or one disease (score=0 or 1). Reference for multimorbidity severity (0-≥5) was no disease (score=0). | | | | | | | | | |

| **Supplementary Table 31.** Landmark 1 stratified for birth year quartiles (Q1-Q4). Subdistributional Hazard Ratios (subHR), incidence rates, and incidence rate ratios with 95% confidence intervals (CI), for venous thromboembolism (VTE) according to multimorbidity score ≥2 and birth year quartiles 1-4 (Q1-Q4) for Landmark 1. | | | | | | | | | |
| --- | --- | --- | --- | --- | --- | --- | --- | --- | --- |
| **Multimorbidity score** | **Person years, No.** | **Cases, No./persons at risk, /No** | **Incidence rate, cases/1000 person-years** | **Incidence rate ratio (95%CI)**  **Model 1 Model 2 Model 3** | | | **subHR (95%CI)**  **Model 1 Model 2 Model 3** | | |
| Q1 Score <2 | 10881273 | 38344/1359468 | 3.52 (3.49-3.56) | 1[Reference] | 1[Reference] | 1[Reference] | 1[Reference] | 1[Reference] | 1[Reference] |
| Q1 Score ≥2 | 4459328 | 23080/623204 | 5.18 (5.11-5.24) | 1.47 (1.45-1.49) | 1.27 (1.25-1.29) | 1.28 (1.26-1.30) | 1.32 (1.30-1.34) | 1.21 (1.19-1.24) | 1.22 (1.20-1.25) |
| Q2 Score <2 | 14852863 | 15825/1698442 | 1.07 (1.05-1.08) | 1[Reference] | 1[Reference] | 1[Reference] | 1[Reference] | 1[Reference] | 1[Reference] |
| Q2 Score ≥2 | 2445186 | 4141/279729 | 1.69 (1.64-1.75) | 1.59 (1.54-1.64) | 1.53 (1.48-1.59) | 1.56 (1.50-1.61) | 1.59 (1.54-1.65) | 1.53 (1.48-1.59) | 1.56 (1.50-1.61) |
| Q3 Score <2 | 15900731 | 6167/1864743 | 0.39 (0.38-0.4) | 1[Reference] | 1[Reference] | 1[Reference] | 1[Reference] | 1[Reference] | 1[Reference] |
| Q3 Score ≥2 | 1203533 | 1229/137419 | 1.02 (0.97-1.08) | 2.63 (2.48-2.80) | 2.22 (2.09-2.37) | 1.96 (1.84-2.08) | 2.71 (2.55-2.88) | 2.28 (2.14-2.43) | 1.98 (1.85-2.11) |
| Q4 Score <2 | 18094412 | 1474/2055261 | 0.08 (0.08-0.09) | 1[Reference] | 1[Reference] | 1[Reference] | 1[Reference] | 1[Reference] | 1[Reference] |
| Q4 Score ≥2 | 446099 | 72/50223 | 0.16 (0.13-0.20) | 1.98 (1.56-2.51) | 1.65 (1.30-2.09) | 1.48 (1.17-1.88) | 2.00 (1.28-2.53) | 1.66 (1.31-2.11) | 1.49 (1.17-1.88) |
| Model 1 is a crude model. Model 2 is adjusted for sex, year of birth, and educational attainment. Model 3 is adjusted for sex, year of birth, educational attainment, country of birth, VTE in sibling, Human immunodeficiency virus (HIV) disease, Chronic viral hepatitis B, Chronic viral hepatitis C, Other thrombophilia, Activated protein C resistance [factor V Leiden mutation], Deficiency antithrombin, Deficiency protein C, Deficiency protein S, Prothrombin gene mutation Q1=1908-1954, Q2=1955-1972, Q3=1973-1990, Q4=1991-2009. Reference with <2 score (one or no disease). | | | | | | | | | |

| **Supplementary Table 32.** Landmark 1 stratified for birth year quartiles (Q1-Q4). Incidence rates, incidence rate ratios, and Subdistributional Hazard Ratios (subHR) with 95% confidence intervals (CI) for venous thromboembolism (VTE) according to multimorbidity score (0 to ≥5) and birth year quartiles 1-4 (Q1-Q4). | | | | | | | | | | |
| --- | --- | --- | --- | --- | --- | --- | --- | --- | --- | --- |
| **Multimorbidity score** | **Person years, No.** | **Cases (n) /persons at risk (n)** | **Incidence rate, cases/1000 person-years** | **Incidence rate ratio (95%CI)**  **Model 1 Model 2 Model 3** | | | **subHR (95%CI)**  **Model 1 Model 2 Model 3** | | |  |
| **Q1=birth years 1908-1954 (n=1982672)** | | | | | | | | | | |
| Score 0 | 8022046 | 27524/1000345 | 3.43 (3.39-3.47) | 1[Reference] | 1[Reference] | 1[Reference] | 1[Reference] | 1[Reference] | 1[Reference] |  |
| Score 1 | 2859227 | 10820/359123 | 3.78 (3.71-3.86) | 1.10 (1.08-1.13) | 1.08 (1.05-1.10) | 1.08 (1.06-1.10) | 1.10 (1.07-1.12) | 1.08 (1.06-1.11) | 1.08 (1.06-1.11) |  |
| Score 2 | 1920137 | 8332/251014 | 4.34 (4.25-4.43) | 1.26 (1.23-1.30) | 1.16 (1.13-1.18) | 1.16 (1.13-1.19) | 1.21 (1.18-1.24) | 1.15 (1.12-1.18) | 1.16 (1.13-1.19) |  |
| Score 3 | 1188132 | 6098/163292 | 5.13 (5.01-5.26) | 1.50 (1.45-1.54) | 1.29 (1.26-1.33) | 1.30 (1.27-1.34) | 1.37 (1.33-1.40) | 1.26 (1.23-1.30) | 1.27 (1.23-1.31) |  |
| Score 4 | 675541 | 3835/98083 | 5.68 (5.50-5.86) | 1.65 (1.60-1.71) | 1.37 (1.32-1.41) | 1.38 (1.34-1.43) | 1.43 (1.39-1.48) | 1.29 (1.24-1.33) | 1.30 (1.26-1.35) |  |
| Score ≥5 | 675516 | 4815/110815 | 7.13 (6.93-7.33) | 2.08 (2.01-2.14) | 1.60 (1.56-1.66) | 1.63 (1.58-1.68) | 1.60 (1.55-1.65) | 1.38 (1.34-1.42) | 1.40 (1.36-1.45) |  |
| **Q2=birth years 1955-1972(n=1978171)** | | | | | | | | | | |
| Score 0 | 11687553 | 11816/1338838 | 1.01 (0.90-1.03) | 1[Reference] | 1[Reference] | 1[Reference] | 1[Reference] | 1[Reference] | 1[Reference] |  |
| Score 1 | 3165310 | 4009/359604 | 1.27 (1.23-1.30) | 1.25 (1.21-1.30) | 1.24 (1.20-1.29) | 1.23 (1.19-1.28) | 1.26 (1.22-1.31) | 1.25 (1.21-1.30) | 1.24 (1.20-1.29) |  |
| Score 2 | 1391418 | 2072/158513 | 1.49 (1.43-1.55) | 1.47 (1.41-1.54) | 1.44 (1.37-1.51) | 1.45 (1.38-1.52) | 1.49 (1.42-1.56) | 1.45 (1.38-1.52) | 1.45 (1.39-1.52) |  |
| Score 3 | 619694 | 1087/70933 | 1.75 (1.65-1.86) | 1.74 (1.63-1.85) | 1.67 (1.57-1.78) | 1.70 (1.60-1.81) | 1.74 (1.64-1.85) | 1.67 (1.57-1.78) | 1.71 (1.60-1.82) |  |
| Score 4 | 263575 | 531/30371 | 2.01 (1.85-2.19) | 1.99 (1.83-2.17) | 1.88 (1.73-2.05) | 1.93 (1.78-2.12) | 1.99 (1.83-2.17) | 1.87 (1.72-2.04) | 1.93 (1.77-2.11) |  |
| Score ≥5 | 170498 | 451/19912 | 2.65 (2.41-2.90) | 2.62 (2.38-2.87) | 2.38 (2.17-2.62) | 2.49 (2.27-2.74) | 2.59 (2.36-2.84) | 2.35 (2.14-2.58) | 2.45 (2.23-2.70) |  |
| **Q3=birth years 1973-1990 (n=2002162)** | | | | | | | | | | |
| Score 0 | 13501217 | 4751/1590314 | 0.35 (0.34-0.36) | 1[Reference] | 1[Reference] | 1[Reference] | 1[Reference] | 1[Reference] | 1[Reference] |  |
| Score 1 | 2399515 | 1416/274429 | 0.59 (0.56-0.62) | 1.68 (1.58-1.78) | 1.56 (1.47-1.66) | 1.41 (1.32-1.49) | 1.73 (1.63-1.84) | 1.61 (1.51-1.70) | 1.42 (1.34-1.51) |  |
| Score 2 | 795802 | 685/90795 | 0.86 (0.80-0.93) | 2.45 (2.26-2.65) | 2.12 (1.96-2.30) | 1.87 (1.72-2.03) | 2.53 (2.34-2.74) | 2.19 (2.02-2.38) | 1.90 (1.75-2.06) |  |
| Score 3 | 273874 | 321/31279 | 1.17 (1.05-1.31) | 3.33 (2.97-3.73) | 2.72 (2.42-3.04) | 2.33 (2.07-2.61) | 3.45 (3.08-3.86) | 2.80 (2.50-3.14) | 2.36 (2.10-2.65) |  |
| Score 4 | 92178 | 131/10551 | 1.42 (1.20-1.69) | 4.04 (3.40-4.80) | 3.15 (2.65-3.75) | 2.65 (2.23-3.16) | 4.18 (3.51-4.97) | 3.25 (2.73-3.87) | 2.68 (2.25-3.20) |  |
| Score ≥5 | 41678 | 92/4794 | 2.21 (1.80-2.71) | 6.27 (5.10-7.71) | 4.65 (3.78-5.72) | 3.85 (3.13-4.74) | 6.49 (5.28-7.98) | 4.79 (3.89-5.91) | 3.87 (3.14-4.78) |  |
| **Q4= birth years 1991-2009 (n=2105484)** | | | | | | | | | | |
| Score 0 | 16024263 | 1222/1821979 | 0.076 (0.072-0.081) | 1[Reference] | 1[Reference] | 1[Reference] | 1[Reference] | 1[Reference] | 1[Reference] |  |
| Score 1 | 2070149 | 252/233282 | 0.12 (0.11-0.14) | 1.60 (1.39-1.83) | 1.47 (1.28-1.68) | 1.33 (1.61-1.53) | 1.61 (1.41-1.84) | 1.48 (1.29-1.70) | 1.33 (1.16-1.53) |  |
| Score 2 | 379267 | 52/42683 | 0.14 (0.10-0.18) | 1.80 (1.36-2.37) | 1.53 (1.16-2.02) | 1.37 (1.04-1.81) | 1.82 (1.38-2.40) | 1.5 (1.17-2.04) | 1.37 (1.04-1.82) |  |
| Score 3 | 56514 | 15/6367 | 0.27 (0.16-0.44) | 3.48 (2.09-5.79) | 2.53 (1.52-4.22) | 2.21 (1.33-3.68) | 3.52 (2.11-5.85) | 2.56 (1.54-4.27) | 2.22 (1.33-3.70) |  |
| Score 4 | 8576 | 4/971 | 0.47 (0.18-1.24) | 6.12 (2.29-16.32) | 3.64 (1.36-9.72) | 3.14 (1.18-8.39) | 6.15 (2.31-16.42) | 3.67 (1.37-9.83) | 3.15 (1.18-8.41) |  |
| Score ≥5 | 1741 | 1/202 | 0.57 (0.0081-4.08) | 7.53 (1.06-53.51) | 4.19 (0-59-29.74) | 3.59 (0.50-25.52) | 7.38 (1.04-52.27) | 4.13 (0.59-29.16) | 3.51 (0.50-24.81) |  |
| Abbreviations: Q=quartile. NA=not applicable due to few cases Model 1 is a crude model (univariate). Model 2 is an adjusted model (multivariate), with adjustments for sex, year of birth, and educational attainment. Model 3 is adjusted for sex, year of birth, educational attainment, country of birth, VTE in sibling, Human immunodeficiency virus (HIV) disease, Chronic viral hepatitis B, Chronic viral hepatitis C, Other thrombophilia, Activated protein C resistance [factor V Leiden mutation], Deficiency antithrombin, Deficiency protein C, Deficiency protein S, Prothrombin gene mutation. Reference = no diseases (score=0). | | | | | | | | | | |

| **Supplementary Table 33.** Landmark 1 stratified for Country of birth (CB). Subdistributional Hazard Ratios (subHR), incidence rates, and incidence rate ratios with 95% confidence intervals (CI), for venous thromboembolism (VTE) according to multimorbidity score ≥2 and Country of birth (CB 1-4) for Landmark 1. | | | | | | | | | |
| --- | --- | --- | --- | --- | --- | --- | --- | --- | --- |
| **Multimorbidity score** | **Person years, No.** | **Cases, No./persons at risk, /No** | **Incidence rate, cases/1000 person-years** | **Incidence rate ratio (95%CI)**  **Model 1 Model 2 Model 3** | | | **subHR (95%CI)**  **Model 1 Model 2 Model 3** | | |
| CB1 Score <2 | 45976595 | 54345/5307226 | 1.18 (1.17-1.19) | 1[Reference] | 1[Reference] | 1[Reference] | 1[Reference] | 1[Reference] | 1[Reference] |
| CB1 Score ≥2 | 6972252 | 24618/894880 | 3.53 (3.49-3.58) | 2.99 (2.94-3.03) | 1.31 (1.29-1.33) | 1.31 (1.28-1.33) | 2.71 (2.67-2.76) | 1.22 (1.20-1.24) | 1.22 (1.20-1.24) |
| CB2 Score <2 | 1286126 | 2132/170498 | 1.66 (1.59-1.73) | 1[Reference] | 1[Reference] | 1[Reference] | 1[Reference] | 1[Reference] | 1[Reference] |
| CB2 Score ≥2 | 338984 | 1401/45803 | 4.13 (3.92-4.36) | 2.49 (2.33-2.67) | 1.39 (1.29-1.49) | 1.38 (1.29-1.48) | 2.47 (2.31-2.65) | 1.33 (1.23-1.43) | 1.32 (1.23-1.42) |
| CB3 Score <2 | 2758556 | 1904/346125 | 0.69 (0.66-0.72) | 1[Reference] | 1[Reference] | 1[Reference] | 1[Reference] | 1[Reference] | 1[Reference] |
| CB3 Score ≥2 | 257683 | 925/33553 | 3.59 (3.37-3.83) | 5.20 (4.81-5.63) | 1.46 (1.34-1.59) | 1.45 (1.33-1.58) | 5.08 (4.69-5.49) | 1.40 (1.27-1.53) | 1.39 (1.27-1.52) |
| CB4 Score <2 | 9708002 | 3429/1154065 | 0.35 (0.34-0.37) | 1[Reference] | 1[Reference] | 1[Reference] | 1[Reference] | 1[Reference] | 1[Reference] |
| CB4 Score ≥2 | 985227 | 1579/116339 | 1.60 (1.52-1.69) | 4.53 (4.27-4.81) | 1.61 (1.51-1.71) | 1.60 (1.50-1.71) | 4.59 (4.33-4.88) | 1.64 (1.53-1.75) | 1.63 (1.52-1.75) |
| Model 1 is a crude model. Model 2 is adjusted for sex, year of birth, and educational attainment. Model 3 is adjusted for sex, year of birth, educational attainment, VTE in sibling, Human immunodeficiency virus (HIV) disease, Chronic viral hepatitis B, Chronic viral hepatitis C, Other thrombophilia, Activated protein C resistance [factor V Leiden mutation], Deficiency antithrombin, Deficiency protein C, Deficiency protein S, Prothrombin gene mutation. CB 1= Swedish born, CB2= Born in the Nordic Countries, except Sweden, CB3= born in Europe, except the Nordic Countries, CB4= born outside Europe. Reference with <2 score (one or no disease). | | | | | | | | | |

| **Supplementary Table 34.** Landmark 1 stratified for Country of birth (CB). Subdistributional Hazard Ratios (subHR), incidence rates, and incidence rate ratios with 95% confidence intervals (CI), for venous thromboembolism (VTE) according to multimorbidity score ≥2 and Country of birth (CB 1-4) for Landmark 1. | | | | | | | | | | |
| --- | --- | --- | --- | --- | --- | --- | --- | --- | --- | --- |
| **Multimorbidity score** | **Person years, No.** | **Cases (n) /persons at risk (n)** | **Incidence rate, cases/1000 person-years** | **Incidence rate ratio (95%CI)**  **Model 1 Model 2 Model 3** | | | **subHR (95%CI)**  **Model 1 Model 2 Model 3** | | |  |
| **CB 1= Swedish born** | | | | | | | | | | |
| Score 0 | 37030172 | 39774/4264829 | 1.07 (1.06-1.08) | 1[Reference] | 1[Reference] | 1[Reference] | 1[Reference] | 1[Reference] | 1[Reference] |  |
| Score 1 | 8946423 | 14571/1042397 | 1.63 (1.50-1.66) | 1.52 (1.49-1.55) | 1.15 (1.12-1.17) | 1.14 (1.12-1.17) | 1.50 (1.47-1.53) | 1.16 (1.14-1.19) | 1.16 (1.14-1.18) |  |
| Score 2 | 3722096 | 9743/451148 | 2.62 (2.57-2.67) | 2.44 (2.38-2.49) | 1.25 (1.22-1.28) | 1.24 (1.21-1.27) | 2.33 (2.28-2.38) | 1.24 (1.22-1.27) | 1.24 (1.21-1.26) |  |
| Score 3 | 1730155 | 6545/221774 | 3.78 (3.69-3.88) | 3.53 (3.43-3.62) | 1.38 (1.34-1.42) | 1.37 (1.34-1.41) | 3.20 (3.12-3.29) | 1.32 (1.28-1.36) | 1.32 (1.28-1.35) |  |
| Score 4 | 827960 | 3816/113187 | 4.61 (4.47-4.76) | 4.29 (4.15-4.44) | 1.41 (1.36-1.46) | 1.41 (1.36-1.45) | 3.67 (3.55-3.79) | 1.27 (1.23-1.32) | 1.28 (1.23-1.32) |  |
| Score ≥5 | 692041 | 4514/108771 | 6.52 (6.34-6.72) | 6.07 (5.89-6.26) | 1.62 (1.57-1.67) | 1.63 (1-57-1.68) | 4.55 (4.41-4.69) | 1.29 (1.25-1.33) | 1.30 (1.26-1.34) |  |
| **CB2= Born in the Nordic Countries, except Sweden** | | | | | | | | | | |
| Score 0 | 1036919 | 1489/139236 | 1.44 (1.36-1.51) | 1[Reference] | 1[Reference] | 1[Reference] | 1[Reference] | 1[Reference] | 1[Reference] |  |
| Score 1 | 249207 | 643/31262 | 2.58 (2.39-2.79) | 1.80 (1.64-1.97) | 1.24 (1.13-1.36) | 1.24 (1.13-1.36) | 1.93 (1.76-2.12) | 1.29 (1.18-1.43) | 1.29 (1.18-1.42) |  |
| Score 2 | 151374 | 482/19441 | 3.18 (3.79-4.65) | 2.22 (2.00-2.46) | 1.27 (1.15-1.41) | 1.27 (1.14-1.41) | 2.34 (2.11-2.59) | 1.31 (1.17-1.45) | 1.30 (1.17-1.45) |  |
| Score 3 | 87974 | 369/11726 | 4.19 (3.79-4.65) | 2.92 (2.61-3.27) | 1.52 (1.35-1.71) | 1.51 (1.35-1.70) | 2.98 (2.66-3.34) | 1.50 (1.33-1.69) | 1.49 (1.32-1.68) |  |
| Score 4 | 49217 | 245/6847 | 4.98 (4.39-5.64) | 3.47 (3.03-3.97) | 1.64 (1.43-1.88) | 1.63 (1.42-1.88) | 3.40 (2.97-3.89) | 1.56 (1.35-1.79) | 1.55 (1.35-1.79) |  |
| Score ≥5 | 50420 | 305/7789 | 6.05 (5.41-6.77) | 4.21 (3.72-4.76) | 1.78 (1.57-2.02) | 1.78 (1.56-2.02) | 3.73 (3.30-4.22) | 1.52 (1.35-1.79) | 1.52 (1.33-1.73) |  |
| **CB3= born in Europe, except the Nordic Countries** | | | | | | | | | | |
| Score 0 | 2498036 | 1431/314439 | 0.57 (0.54-0.60) | 1[Reference] | 1[Reference] | 1[Reference] | 1[Reference] | 1[Reference] | 1[Reference] |  |
| Score 1 | 260521 | 473/31686 | 1.82 (1.66-1.99) | 3.17 (2.85-3.52) | 1.38 (1.24-1.54) | 1.37 (1.23-1.53) | 3.30 (2.97-3.66) | 1.46 (1.30-1.63) | 1.45 (1.29-1.62) |  |
| Score 2 | 12749 | 347/15347 | 2.80 (2.52-3.12) | 4.89 (4.35-5.50) | 1.53 (1.36-1.74) | 1.52 (1.35-1.72) | 5.02 (4.46-5.64) | 1.58 (1.39-1.80) | 1.57 (1.38-1.78) |  |
| Score 3 | 65742 | 220/8451 | 3.35 (2.93-3.82) | 5.84 (5.07-6.73) | 1.47 (1.27-1.71) | 1.47 (1.26-1.70) | 5.79 (5.03-6.68) | 1.47 (1.36-1.72) | 1.46 (1.25-1.72) |  |
| Score 4 | 34194 | 153/4629 | 4.47 (3.82-5.24) | 7.81 (6.61-9.23) | 1.71 (1.44-2.04) | 1.70 (1.43-2.02) | 7.38 (6.24-8.72) | 1.61 (1.35-1.93) | 1.60 (1.34-1.92) |  |
| Score ≥5 | 33999 | 205/5126 | 6.03 (5.26-6.91) | 10.53 (9.09-12.18) | 1.89 (1.62-2.21) | 1.88 (1.61-2.20) | 9.00 (7.77-10.42) | 1.62 (1.37-1.90) | 1.60 (1.36-1.89) |  |
| **CB4= born outside Europe** | | | | | | | | | | |
| Score 0 | 8669951 | 2619/1032972 | 0.30 (0.29-0.31) | 1[Reference] | 1[Reference] | 1[Reference] | 1[Reference] | 1[Reference] | 1[Reference] |  |
| Score 1 | 1038051 | 810/121093 | 0.78 (0.73-0.84) | 2.58 (2.39-2.79) | 1.37 (1.26-1.49) | 1.36 (1.26-1.48) | 2.64 (2.44-2.86) | 1.44 (1.32-1.56) | 1.43 (1.31-1.55) |  |
| Score 2 | 49407 | 569/57069 | 1.16 (1.07-1.26) | 3.85 (3.52-4.21) | 1.53 (1.39-1.68) | 1.52 (1.39-1.67) | 3.95 (3.61-4.32) | 1.61 (1.47-1.78) | 1.60 (1.46-1.77) |  |
| Score 3 | 254345 | 387/29921 | 1.52 (1.38-1.68) | 5.04 (4.53-5.60) | 1.65 (1.47-1.84) | 1.64 (1.47-1.83) | 5.13 (4.61-5.71) | 1.72 (1.52-1.92) | 1.71 (1.53-1.91) |  |
| Score 4 | 128500 | 287/15313 | 2.23 (1.99-2.51) | 7.39 (6.55-9.35) | 2.09 (1.84-2.37) | 2.07 (1.83-2.35) | 7.46 (6.61-8.43) | 2.14 (1.88-2.44) | 2.13 (1.87-2.43) |  |
| Score ≥5 | 112975 | 335/14037 | 2.97 (2.66-3.30) | 9.82 (8.76-11.00) | 2.24 (1.98-2.52) | 2.23 (1.97-2.51) | 9.54 (8.51-10.69) | 2.18 (1.92-2.46) | 2.16 (1.91-2.43) |  |
| Abbreviations: Q=quartile. NA=not applicable due to few cases Model 1 is a crude model (univariate). Model 2 is an adjusted model (multivariate), with adjustments for sex, year of birth, and educational attainment. Model 3 is adjusted for sex, year of birth, educational attainment, VTE in sibling, Human immunodeficiency virus (HIV) disease, Chronic viral hepatitis B, Chronic viral hepatitis C, Other thrombophilia, Activated protein C resistance [factor V Leiden mutation], Deficiency antithrombin, Deficiency protein C, Deficiency protein S, Prothrombin gene mutation. Reference with <2 score (one or no disease). | | | | | | | | | | |

| **Supplementary Table 35**. Subdistributional Hazard Ratios (subHR), incidence rates, and incidence rate ratios with 95% confidence intervals (CI) for venous thromboembolism (VTE) according to multimorbidity for Landmark 1 based on 45 diseases and stratified for nine different multimorbidity disease clusters (F1-F9) [6]. | | | | | | | | | |
| --- | --- | --- | --- | --- | --- | --- | --- | --- | --- |
| **Multimorbidity score** | **Person years, No.** | **Cases, No./persons at risk, /No** | **Incidence rate, cases/1000 person-years** | **Incidence rate ratio (95%CI)**  **Model 1 Model 2 Model 3** | | | **subHR (95%CI)**  **Model 1 Model 2 Model 3** | | |
| F1 Score <2 | 66533675 | 81382/7815137 | 1.22 (1.21-1.23) | 1[Reference] | 1[Reference] | 1[Reference] | 1[Reference] | 1[Reference] | 1[Reference] |
| F1 Score ≥2 | 17949750 | 8950/253352 | 5.11 (5.01-5.22) | 4.18 (4.09-4.27) | 1.2 (1.18-1.23) | 1.22 (1.19-1.25) | 3.44 (3.37-3.52) | 1.05 (1.03-1.07) | 1.07 (1.04-1.09) |
| F2 Score <2 | 67157489 | 87787/7933833 | 1.31 (1.30-1.32) | 1[Reference] | 1[Reference] | 1[Reference] | 1[Reference] | 1[Reference] | 1[Reference] |
| F2 Score ≥2 | 1125936 | 2545/134656 | 2.23 (2.17-2.35) | 1.72 (1.66-1.80) | 1.48 (1.42-1.54) | 1.47 (1.41-1.53) | 1.72 (1.65-1.79) | 1.46 (1.40-1.51) | 1.44 (1.38-1.49) |
| F3 Score <2 | 68275353 | 90299/8067377 | 1.32 (1.31-1.33) | 1[Reference] | 1[Reference] | 1[Reference] | 1[Reference] | 1[Reference] | 1[Reference] |
| F3 Score ≥2 | 8072 | 33/1122 | 4.09 (2.91-5.75) | 3.09 (2.20-4.35) | 1.83 (1.3-2.58) | 1.72 (1.22-2.42) | 2.73 (1.95-3.82) | 1.63 (1.15-2.29) | 1.51 (1.07-2.13) |
| F4 Score <2 | 6812811 | 89252/8042463 | 1.31 (1.30-1.32) | 1[Reference] | 1[Reference] | 1[Reference] | 1[Reference] | 1[Reference] | 1[Reference] |
| F4 Score ≥2 | 155313 | 1080/26026 | 6.95 (6.55-7.38) | 5.31 (5.00-5.64) | 1.34 (1.26-1.42) | 1.37 (1.29-1.45) | 3.82 (3.59-4.06) | 1.04 (0.97-1.10) | 1.06 (1.00-1.13) |
| F5 Score <2 | 68169605 | 89696/8052381 | 1.32 (1.31-1.32) | 1[Reference] | 1[Reference] | 1[Reference] | 1[Reference] | 1[Reference] | 1[Reference] |
| F5 Score ≥2 | 113820 | 636/16108 | 5.59 (5.17-6.04) | 4.25 (3.93-4.59) | 1.30 (1.21-1.41) | 1.31 (1.21-1.42) | 3.61 (3.34-3.90) | 1.20 (1.11-1.30) | 1.20 (1.11-1.30) |
| F6 Score <2 | 67494685 | 86524/7965796 | 1.28 (1.27-1.29) | 1[Reference] | 1[Reference] | 1[Reference] | 1[Reference] | 1[Reference] | 1[Reference] |
| F6 Score ≥2 | 788739 | 3808/102693 | 4.82 (4.68-4.98) | 3.77 (3.65-3.89) | 1.26 (1.22-1.30) | 1.27 (1.23-1.32) | 3.45 (3.36-3.58) | 1.27 (1.23-1.31) | 1.29 (1.24-1.33) |
| F7 Score <2 | 68279250 | 90307/8067812 | 1.32 (1.31-1.33) | 1[Reference] | 1[Reference] | 1[Reference] | 1[Reference] | 1[Reference] | 1[Reference] |
| F7 Score ≥2 | 4175 | 25/677 | 5.99 (4.05-8.86) | 4.53 (3.06-6.70) | 1.23 (0.83-1.82) | 1.26 (0.85-1.87) | 3.36 (2.67-4.98) | 0.98 (0.66-1.46) | 1.01 (0.68-1.50) |
| F8 Score <2 | 67485250 | 88276/7969676 | 1.31 (1.30-1.32) | 1[Reference] | 1[Reference] | 1[Reference] | 1[Reference] | 1[Reference] | 1[Reference] |
| F8 Score ≥2 | 798175 | 2056/98813 | 2.58 (2.47-2.69) | 1.97 (1.88-2.06) | 1.52 (1.46-1.59) | 1.53 (1.47-1.60) | 1.90 (1.81-1.98) | 1.37 (1.31-1.43) | 1.38 (1.32-1.44) |
| F9 Score <2 | 68283188 | 90331/8068441 | 1.32 (1.31-1.33) | 1[Reference] | 1[Reference] | 1[Reference] | 1[Reference] | 1[Reference] | 1[Reference] |
| F9 Score ≥2 | 237 | 1/48 | 4.22 (0.59-29.98) | 3.20 (0.45-22.66) | 1.11 (0.16-7.87) | 1.09 (0.15-7.71) | 1.91 (0.29-12.72) | 0.70 (0.10-4.93) | 0.68 (0.10-4.81) |
| Model 1 is a crude model (univariable). Model 2 is an adjusted model (multivariable), with adjustments for year of birth, sex, and educational attainment. Model 3 is adjusted for sex, year of birth, educational attainment, country of birth, VTE in sibling, Human immunodeficiency virus (HIV) disease, Chronic viral hepatitis B, Chronic viral hepatitis C, Other thrombophilia, Activated protein C resistance [factor V Leiden mutation], Deficiency antithrombin, Deficiency protein C, Deficiency protein S, Prothrombin gene mutation.  Abbreviations: NA=not applicable due to few cases; F1=hypertension, heart failure, coronary heart disease, diabetes, obesity, atrial fibrillation, gout, atherosclerosis, and renal disease;F2=affective disorders, anxiety, psychoactive substance misuse, alcohol misuse disorders, anorexia or bulimia, and schizophrenia disorders; F3=inflammatory bowel disease, liver disease, pancreatic disease, and ulcers; F4=epilepsy, blindness and poor vision, cerebrovascular disease, cancer, and impaired or hearing loss; F5=connective tissue disease, osteoporosis, thyroid disorders, and psoriasis; F6=prostate disease, arthrosis, painful back condition, diverticular disease of intestine, and chronic sinusitis; F7=bronchiectasis, Parkinson’s disease, glaucoma, learning disability, and irritable bowel syndrome; F8=asthma, dermatitis and eczema, constipation, chronic obstructive pulmonary disease, and migraine; and F9=multiple sclerosis and dementia. Reference with one or no disease (score=0 or 1). | | | | | | | | | |

| **Supplementary Table 36.** Landmark 1 multimorbidity severity in F1-F9 disease clusters. Incidence rates, incidence rate ratios, and Subdistributional Hazard Ratios (subHR) with 95% confidence intervals (CI) for VTE (venous thromboembolism) for disease clusters F1-F9 according to multimorbidity score (0 to ≥5) in Landmark 1. All (n=8068489) | | | | | | | | | |
| --- | --- | --- | --- | --- | --- | --- | --- | --- | --- |
| **Score (0 to ≥5)** | **Person years, No.** | **Cases, No./persons at risk, /No** | **Incidence rate, cases/1000 person-years** | **Incidence rate ratio (95%CI)**  **Model 1 Model 2 Model 3** | | | **subHR (95% CI)**  **Model 1 Model 2 Model 3** | | |
| **F1=hypertension, heart failure, coronary heart disease, diabetes, obesity, atrial fibrillation, gout, atherosclerosis, and renal disease (9 diseases)** | | | | | | | | | |
| F1 Score 0 | 62167141 | 66222/7264349 | 1.07 (1.06-1.07) | 1[Reference] | 1[Reference] | 1[Reference] | 1[Reference] | 1[Reference] | 1[Reference] |
| F1 Score 1 | 4366533 | 15160/550698 | 3.47 (3.42-3.53) | 3.26 (3.20-3.32) | 1.15 (1.13-1.17) | 1.15 (1.13-1.17) | 3.05 (3.00-3.11) | 1.15 (1.13-1.17) | 1.15 (1.13-1.17) |
| F1 Score 2 | 1296610 | 6156/179935 | 4.75 (4.63-4.87) | 4.46 (4.34-4.57) | 1.20 (1.17-1.23) | 1.21 (1.18-1.24) | 3.81 (3.71-3.91) | 1.10 (1.07-1.13) | 1.12 (1.08-1.15) |
| F1 Score 3 | 359765 | 2048/55478 | 5.69 (5.45-5.94) | 5.35 (5.11-5.58) | 1.31 (1.25-1.37) | 1.33 (1.28-1.40) | 4.12 (3.94-4.31) | 1.07 (1.02-1.12) | 1.09 (1.04-1.14) |
| F1 Score 4 | 75849 | 582/13910 | 7.67 (7.07-8.32) | 7.20 (6.64-7.82) | 1.54 (1.42-1.67) | 1.59 (1.46-1.72) | 4.71 (4.33-5.12) | 1.07 (0.98-1.16) | 1.11 (1.02-1.20) |
| F1 Score ≥5 | 17527 | 164/4029 | 9.36 (8.03-10.90) | 8.78 (7.54-10.24) | 1.71 (1.46-1.99) | 1.79 (1.53-2.08) | 4.58 (3.92-5.34) | 0.97 (0.83-1.13) | 1.01 (0.87-1.18) |
| **F2=affective disorders, anxiety, psychoactive substance misuse, alcohol misuse disorders, anorexia or bulimia, and schizophrenia disorders (6 diseases)** | | | | | | | | |  |
| F2 Score 0 | 63674405 | 80160/7513218 | 1.26 (1.25-1.27) | 1[Reference] | 1[Reference] | 1[Reference] | 1[Reference] | 1[Reference] | 1[Reference] |
| F2 Score 1 | 3484084 | 7627/420615 | 2.19 (2.14-2.24) | 1.74 (1.70-1.78) | 1.30 (1.27-1.33) | 1.29 (1.26-1.33) | 1.71 (1.67-1.75) | 1.25 (1.22-1.28) | 1.24 (1.21-1.27) |
| F2 Score 2 | 1033201 | 2284/123251 | 2.21 (2.12-2.30) | 1.76 (1.68-1.83) | 1.46 (1.40-1.53) | 1.46 (1.40-1.52) | 1.75 (1.67-1.82) | 1.43 (1.37-1.49) | 1.43 (1.37-1.49) |
| F2 Score 3 | 83188 | 232/10209 | 2.79 (2.45-3.17) | 2.22 (1.95-2.52) | 2.14 (1.88-2.43) | 1.96 (1.72-2.23) | 2.15 (1.89-2.44) | 2.05 (1.80-2.33) | 1.87 (1.65-2.13) |
| F2 Score 4 | 9244 | 28/1156 | 3.03 (2.09-4.39) | 2.41 (1.66-3.48) | 2.87 (1.98-4.15) | 2.39 (1.65-3.46) | 2.29 (1.58-3.32) | 2.67 (2.84-3.87) | 2.21 (1.52-3.21) |
| F2 Score ≥5 | 304 | 1/40 | 3.29 (0.46-23.38) | 2.62 (0.37-18.57) | 4.43 (0.62-31.44) | 3.67 (0.52-26.05) | 2.39 (0.033-17.17) | 3.57 (0.49-25.95) | 3.03 (0.41-22.17) |
| **F3=inflammatory bowel disease, liver disease, pancreatic disease, and ulcers (4 diseases)** | | | | | | | | | |
| F3 Score 0 | 67956418 | 89195/8026771 | 1.31 (1.30-1.32) | 1[Reference] | 1[Reference] | 1[Reference] | 1[Reference] | 1[Reference] | 1[Reference] |
| F3 Score 1 | 318935 | 1104/40606 | 3.46 (3.26-3.67) | 2.64 (2.49-2.80) | 1.55 (1.46-1.65) | 1.54 (1.45-1.64) | 2.47 (2.33-2.62) | 1.46 (1.37-1.55) | 1.44 (1.36-1.53) |
| F3 Score 2 | 7892 | 29/1085 | 3.67 (2.55-5.29) | 2.80 (1.95-4.03) | 1.66 (1.15-2.39) | 1.56 (1.08-2.25) | 2.43 (1.69-3.50) | 1.47 (1.02-2.12) | 1.37 (0.95-1.98) |
| F3 Score 3 | 180 | 4/27 | 22.23 (0.83-59.22) | 16.94 (6.36-45.14) | 9.64 (3.62-25.69) | 8.52 (3.20-22.70) | 13.99 (5.48-35.69) | 8.35 (3.31-21.08) | 6.76 (2.56-17.83) |
| F3 Score 4 | NA | NA | NA | NA | NA | NA | NA | NA | NA |
| F3 Score ≥5 | NA | NA | NA | NA | NA | NA | NA | NA | NA |
| **F4=epilepsy, blindness and poor vision, cerebrovascular disease, cancer, and impaired or hearing loss (5 diseases)** | | | | | | | | | |
| F4 Score 0 | 66295717 | 81211/7791491 | 1.22 (1.22-1.23) | 1[Reference] | 1[Reference] | 1[Reference] | 1[Reference] | 1[Reference] | 1[Reference] |
| F4 Score 1 | 1832394 | 8041/250972 | 4.39 (4.29-4.49) | 3.58 (3.50-3.67) | 1.31 (1.28-1.34) | 1.32 (1.29-1.35) | 3.12 (3.05-3.19) | 1.17 (1.14-1.20) | 1.18 (1.15-1.21) |
| F4 Score 2 | 143097 | 962/23709 | 6.72 (6.31-7.16) | 5.49 (5.15-5.85) | 1.35 (1.27-1.44) | 1.38 (1.30-1.48) | 3.97 (3.73-4.24) | 1.05 (0.98-1.17) | 1.08 (1.01-1.15) |
| F4 Score 3 | 11570 | 111/2182 | 9.59 (7.97-11.56) | 7.83 (6.50-9.43) | 1.70 (1.41-2.05) | 1.74 (1.44-2.10) | 5.02 (4.16-6.06) | 1.18 (0.97-1.43) | 1.21 (1.00-1.46) |
| F4 Score 4 | 622 | 7/132 | 11.26 (5.37-23.61) | 9.19 (4.38-19.28) | 1.97 (0.94-4.14) | 2.04 (0.97-4.27) | 5.25 (2.49-11.10) | 1.21 (0.57-2.58) | 1.26 (0.59-2.67) |
| F4 Score ≥5 | 25 | 0/3 | 0 | - | - | - | - | - | - |
| **F5=connective tissue disease, osteoporosis, thyroid disorders, and psoriasis; (4 diseases)** | | | | | | | | | |
| F5 Score 0 | 66454211 | 83803/7833910 | 1.26 (1.25-1.27) | 1[Reference] | 1[Reference] | 1[Reference] | 1[Reference] | 1[Reference] | 1[Reference] |
| F5 Score 1 | 1715394 | 5893/218471 | 3.44 (3.35-3.52) | 2.72 (2.65-2.80) | 1.80 (1.15-1.21) | 1.80 (1.15-1.21) | 2.55 (2.48-2.61) | 1.15 (1.12-1.18) | 1.15 (1.12-1.18) |
| F5 Score 2 | 107526 | 579/15152 | 5.38 (4.96-5.84) | 4.27 (3.93-4.63) | 1.29 (1.19-1.40) | 1.30 (1.19-1.41) | 3.63 (3.35-3.94) | 1.19 (1.09-1.29) | 1.19 (1.10-1.29) |
| F5 Score 3 | 6075 | 57/924 | 9.38 (7.24-12.16) | 7.44 (5.74-9.65) | 1.93 (1.49-2.50) | 1.94 (1.50-2.52) | 5.96 (4.60-7.74) | 1.72 (1.32-2.24) | 1.74 (1.33-2.26) |
| F5 Score 4 | 220 | 0/32 | 0 | - | - | - | - | - | - |
| F5 Score ≥5 | NA | NA | NA | NA | NA | NA | NA | NA | NA |
| **F6=prostate disease, arthrosis, painful back condition, diverticular disease of intestine, and chronic sinusitis (5 diseases)** | | | | | | | | | |
| F6 Score 0 | 61354930 | 70059/7220646 | 1.14 (1.13-1.15) | 1[Reference] | 1[Reference] | 1[Reference] | 1[Reference] | 1[Reference] | 1[Reference] |
| F6 Score 1 | 6139755 | 16465/745150 | 2.68 (2.64-2.72) | 2.35 (2.31-2.39) | 1.22 (1.20-1.24) | 1.23 (1.21-1.25) | 2.29 (2.25-2.33) | 1.24 (1.22-1.26) | 1.24 (1.22-1.27) |
| F6 Score 2 | 726834 | 3428/94260 | 4.72 (4.56-4.88) | 4.13 (3.99-4.27) | 1.31 (1.26-1.35) | 1.33 (1.28-1.37) | 3.81 (3.68-3.94) | 1.33 (1.28-1.37) | 1.34 (1.30-1.39) |
| F6 Score 3 | 59843 | 365/8140 | 6.10 (5.50-6.76) | 5.34 (4.83-5.92) | 1.35 (1.22-1.50) | 1.37 (1.24-1.52) | 4.71 (4.25-5.22) | 1.36 (1.22-1.51) | 1.38 (1.24-1.53) |
| F6 Score 4 | 2040 | 15/290 | 7.35 (4.43-12.20) | 6.44 (3.88-10.68) | 1.46 (0.88-2.43) | 1.50 (0.91-2.49) | 5.50 (3.31-9.13) | 1.41 (0.85-2.36) | 1.44 (0.86-2.41) |
| F6 Score ≥5 | 22 | 0/3 | - | - | - | - | - | - | - |
| **F7=bronchiectasis, Parkinson’s disease, glaucoma, learning disability, and irritable bowel syndrome (5 diseases)** | | | | | | | | | |
| F7 Score 0 | 67592909 | 88418/7980714 | 1.31 (1.30-1.32) | 1[Reference] | 1[Reference] | 1[Reference] | 1[Reference] | 1[Reference] | 1[Reference] |
| F7 Score 1 | 686340 | 1889/87098 | 2.75 (2.63-2.88) | 2.1 (2.01-2.20) | 1.13 (1.08-1.88) | 1.15 (1.09-1.20) | 1.97 (1.88-2.06) | 1.04 (0.99-1.09) | 1.05 (1.00-1.10) |
| F7 Score 2 | 4158 | 25/674 | 6.01 (4.06-8.90) | 4.60 (3.11-6.80) | 1.24 (0.83-1.83) | 1.27 (0.86-1.88) | 3.41 (2.30-5.06) | 0.98 (0.66-1.46) | 1.02 (0.68-1.51) |
| F7 Score 3 | 17 | 0/3 | - | - | - | - | - | - | - |
| F7 Score 4 | NA | NA | NA | NA | NA | NA | NA | NA | NA |
| F7 Score ≥5 | NA | NA | NA | NA | NA | NA | NA | NA | NA |
| **F8=asthma, dermatitis and eczema, constipation, chronic obstructive pulmonary disease, and migraine (5 diseases)** | | | | | | | | | |
| F8 Score 0 | 6131114 | 77156/7232314 | 1.26 (1.25-1.28) | 1[Reference] | 1[Reference] | 1[Reference] | 1[Reference] | 1[Reference] | 1[Reference] |
| F8 Score 1 | 6173798 | 11120/737362 | 1.80 (1.77-1.84) | 1.43 (1.40-1.46) | 1.26 (1.23-1.28) | 1.26 (1.23-1.29) | 1.42 (1.39-1.45) | 1.20 (1.18-1.22) | 1.20 (1.18-1.22) |
| F8 Score 2 | 737793 | 1847/90856 | 2.50 (2.39-2.62) | 1.99 (1.90-2.08) | 1.56 (1.49-1.64) | 1.57 (1.50-1.65) | 1.92 (1.83-2.01) | 1.41 (1.35-1.48) | 1.42 (1.35-1.49) |
| F8 Score 3 | 57846 | 198/7599 | 3.42 (2.97-3.93) | 2.72 (2.36-3.12) | 1.58 (1.37-1.81) | 1.61 (1.40-1.85) | 2.48 (2.16-2.85) | 1.34 (1.16-1.54) | 1.37 (1.19-1.57) |
| F8 Score 4 | 2400 | 11/354 | 4.58 (2.54-8.28) | 3.64 (2.02-6.58) | 1.51 (0.84-2.73) | 1.58 (0.88-2.86) | 3.02 (1.70-5.39) | 1.14 (0.62-2.06) | 1.19 (0.65-2.15) |
| F8 Score ≥5 | 36 | 0/4 | 0 | 0 | - | - | - | - | - |
| **F9=multiple sclerosis and dementia (2 diseases)** | | | | | | | | | |
| F9 Score 0 | 68123236 | 89299/8030629 | 1.31 (1.30-1.32) | 1[Reference] | 1[Reference] | 1[Reference] | 1[Reference] | 1[Reference] | 1[Reference] |
| F9 Score 1 | 159952 | 1032/37812 | 6.45 (6.07-6.86) | 4.92 (4.63-5.23) | 1.08 (1.01-1.14) | 1.10 (1.04-1.17) | 2.49 (2.34-2.65) | 0.57 (0.53-0.60) | 0.58 (0.55-0.62) |
| F9 Score 2 | 237 | 1/48 | 4.22 (0.59-29.98) | 3.22 (0.45-22.87) | 1.11 (0.16-7.88) | 1.09 (0.15-7.72) | 1.87 (0.27-13.12) | 0.69 (0.10-4.85) | 0.68 (0.10-4.74) |
| F9 Score 3 | NA | NA | NA | NA | NA | NA | NA | NA | NA |
| F9 Score 4 | NA | NA | NA | NA | NA | NA | NA | NA | NA |
| F9 Score ≥5 | NA | NA | NA | NA | NA | NA | NA | NA | NA |
| Abbreviations: NA=not applicable due to few cases. Model 1 is a crude model. Model 2 is adjusted for sex, year of birth and educational attainment. Model 3 is adjusted for sex, year of birth, educational attainment, country of birth, VTE in sibling, Human immunodeficiency virus (HIV) disease, Chronic viral hepatitis B, Chronic viral hepatitis C, Other thrombophilia, Activated protein C resistance [factor V Leiden mutation], Deficiency antithrombin, Deficiency protein C, Deficiency protein S, Prothrombin gene mutation. Reference with no disease (score=0). | | | | | | | | | |

| **Supplementary Table 37.**Subdistributional Hazard Ratios (subHR), with 95% confidence intervals (CI) for multimorbidity for Landmark 1 based on 45 diseases. Reference for multimorbidity was one or no disease (score=0 or 1). | | | | | |
| --- | --- | --- | --- | --- | --- |
| **Multimorbidity score** |  |  |  |  |  |
|  | REF | Model 0 | Model 1 | Model 2 | Model 3 |
| Score ≥2 | Score <2 | 2.98 (2.94-3.03) | 1.25 (1.23-1.27) | 2.98 (2.94-3.02) | 2.64 (2.60-2.68) |
| Birth year |  |  | 0.96 (0.96-0.96) |  |  |
| Sex | Male |  |  | 1.02 (1.01-1.04) |  |
| Education |  |  |  |  |  |
| Level 2 | Level 1 |  |  |  | 0.67 (0.66-0.68) |
| Level 3 |  |  |  |  | 0.42 (0.41-0.43) |
| Education level was defined as level 1 = primary school only (9 years in Sweden) or missing value, level 2 = 10-11 years, or level 3 = more than 11 years of education. | | | | | |
|  | | | | | |

| **Supplementary Table 38**. Subdistributional Hazard Ratios (subHR), with 95% confidence intervals (CI) for multimorbidity for Landmark 2 based on 45 diseases. Reference for multimorbidity was one or no disease (score=0 or 1). | | | | | |
| --- | --- | --- | --- | --- | --- |
| **Multimorbidity score** |  |  |  |  |  |
|  | REF | Model 0 | Model 1 | Model 2 | Model 3 |
| Score ≥2 | Score <2 | 3.50 (3.44-3.56) | 1.37 (1.35-1.40) | 3.54 (3.48-3.60) | 3.15 (3.11-3.21) |
| Birth year |  |  | 0.95 (0.95-0.96) |  |  |
| Sex | Male |  |  | 0.89 (0.87-0.90) |  |
| Education |  |  |  |  |  |
| Level 2 | Level 1 |  |  |  | 0.67 (0.65-0.68) |
| Level 3 |  |  |  |  | 0.43 (0.42-0.44) |
| Education level was defined as level 1 = primary school only (9 years in Sweden) or missing value, level 2 = 10-11 years, or level 3 = more than 11 years of education. | | | | | |

**
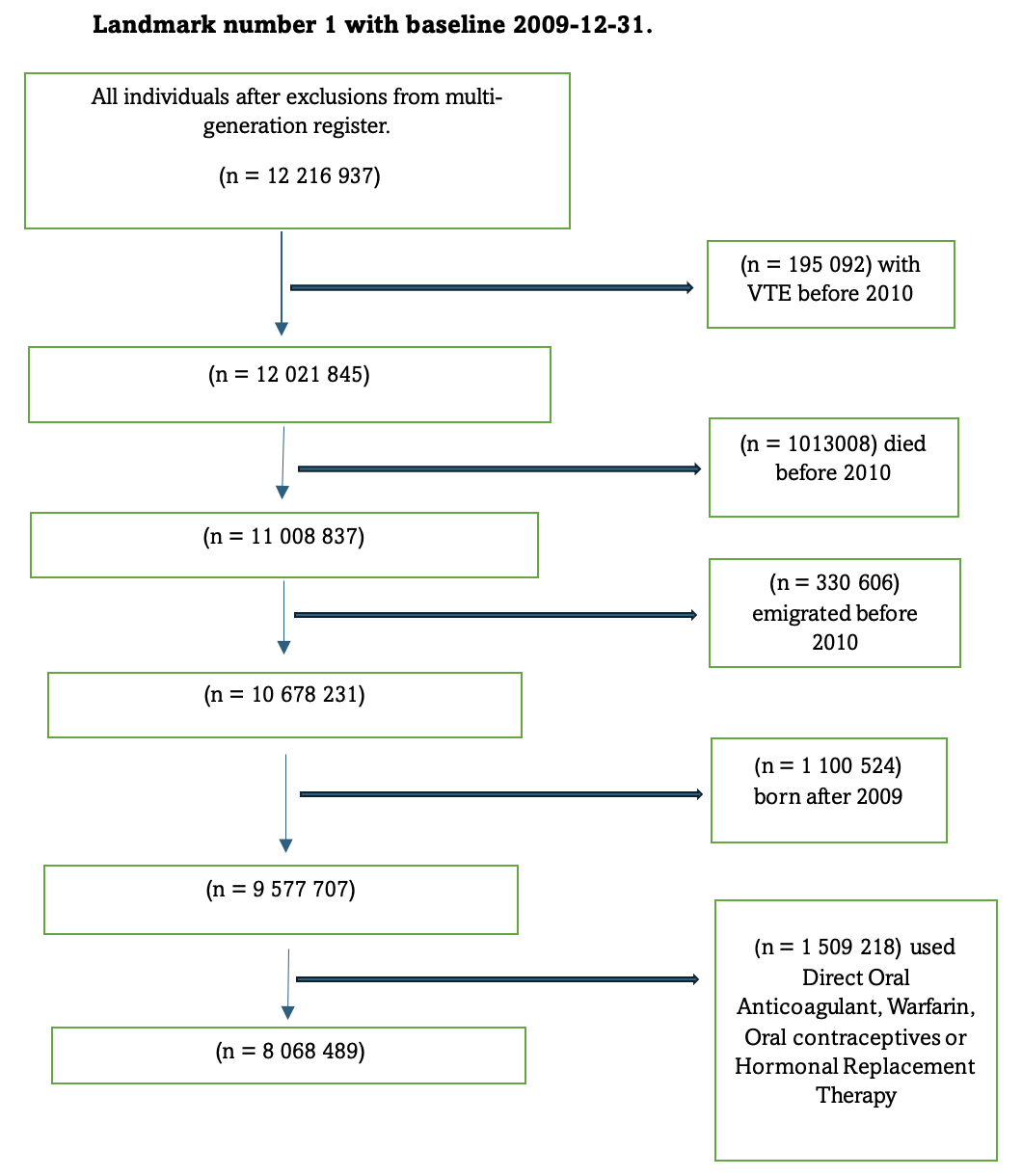
**

**Supplementary Figure 1. Flowchart illustrating the study design, including inclusion and exclusion steps used to derive the final study population for Landmark 1.**

**
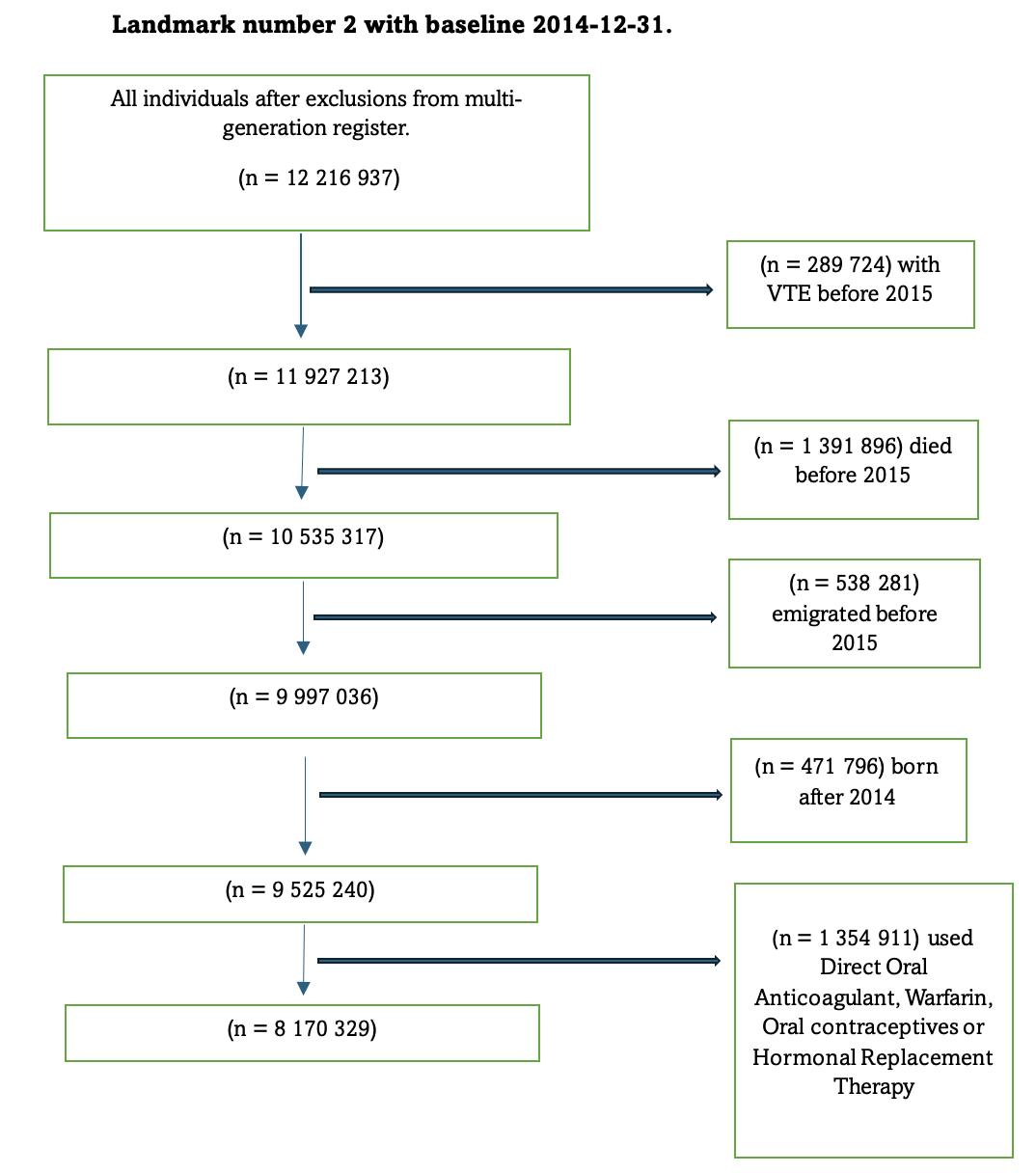
**

**Supplementary Figure 2. Flowchart illustrating the study design, including inclusion and exclusion steps used to derive the final study population for Landmark 2.**

**
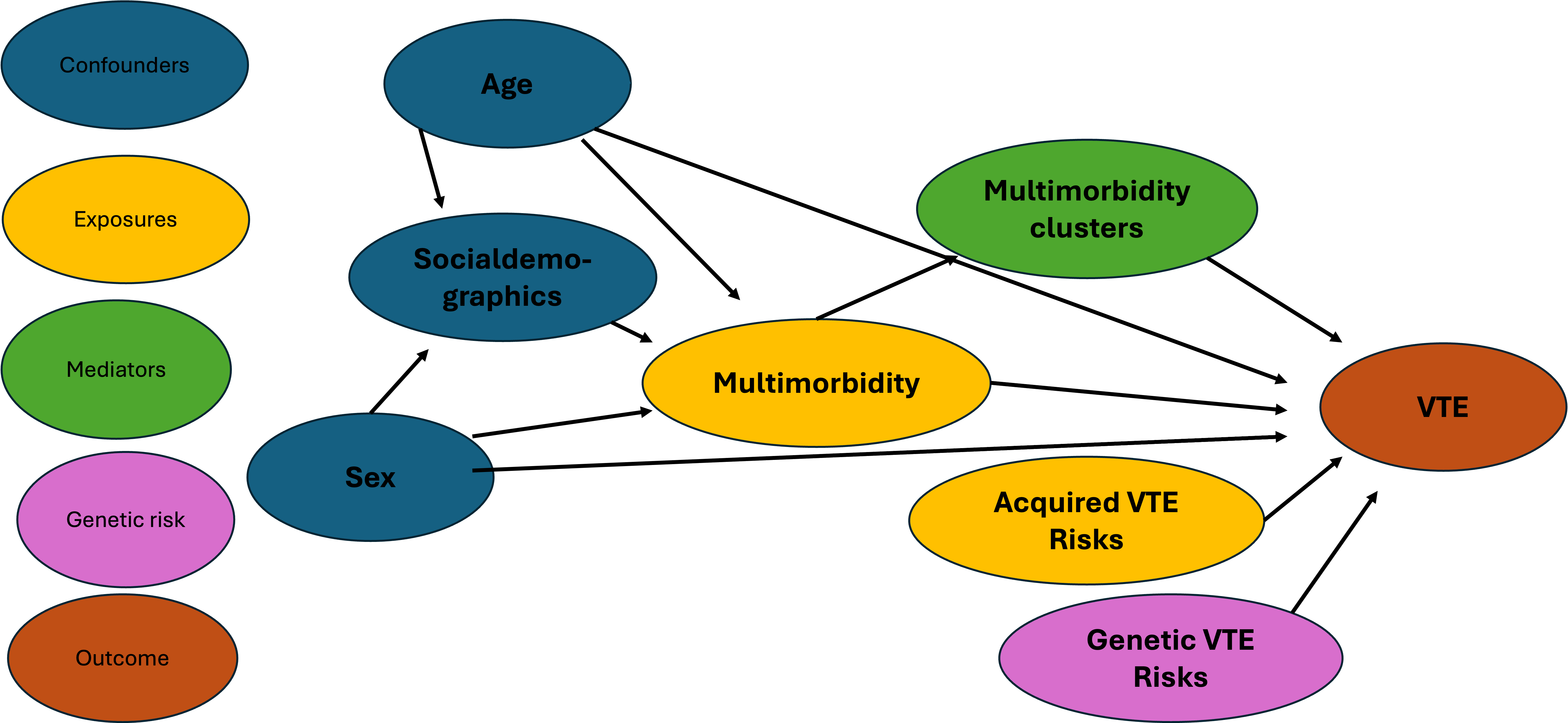
**

**Supplementary Figure 3** Directed acyclic graph (DAG) illustrating the assumed causal relationships between exposure, outcome and covariates.


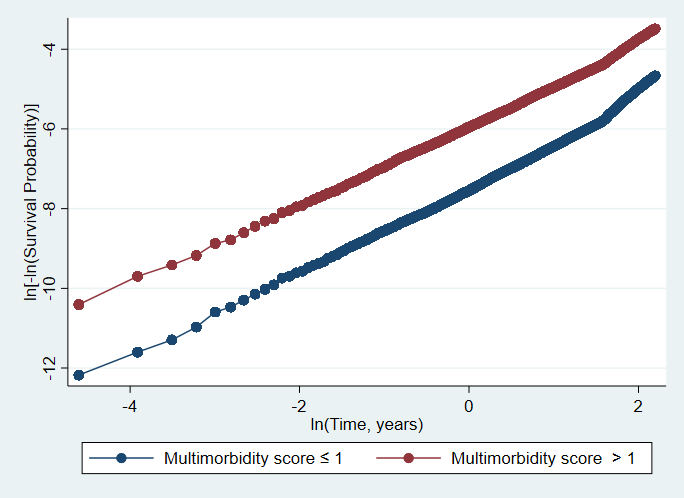

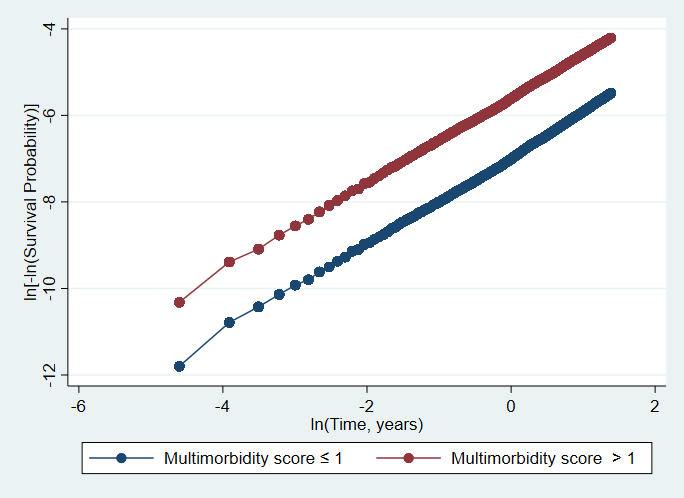


**Supplementary Figure 4.** Log-log function curves for the multimorbidity score, check for the proportional Hazards assumption. The Y-axis represents the log-log transformed survival function


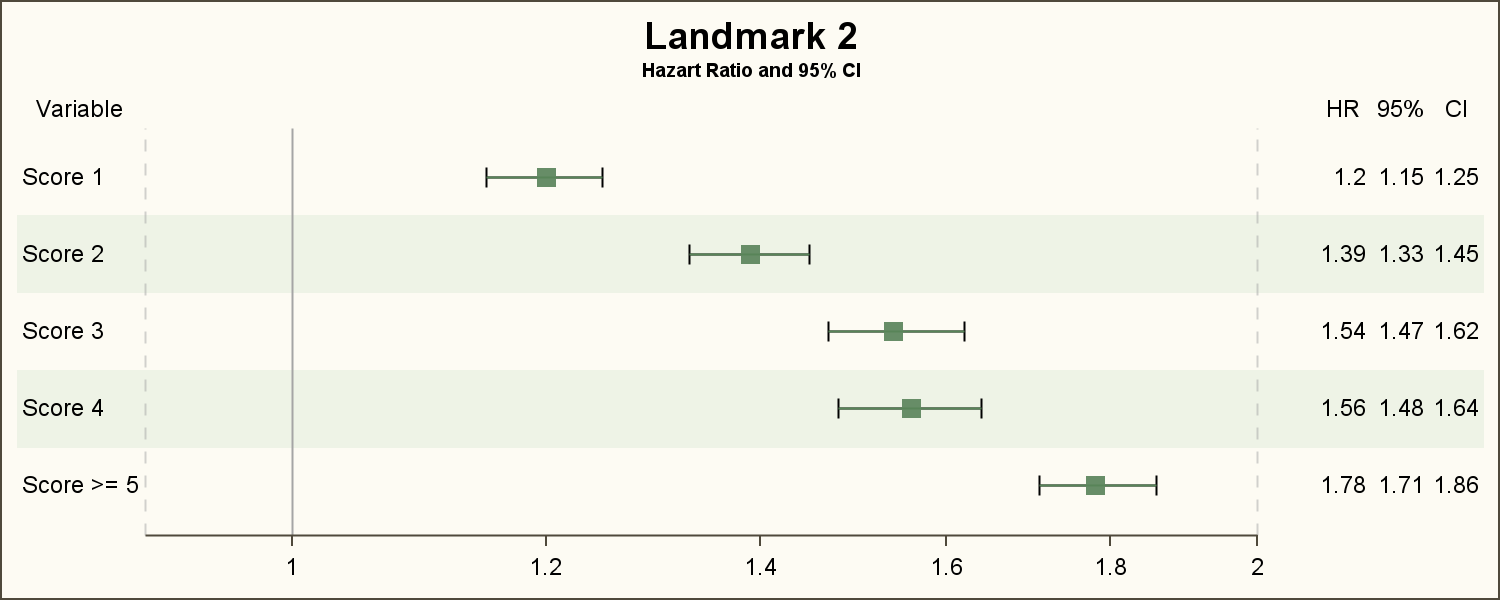


**Supplementary Figure 5.** Forest plot for Subdistribution Hazard Ratio (HR) for venous thromboembolism in Landmark 2 according to multimorbidity score 1-≥5. Reference was no disease (score=0).


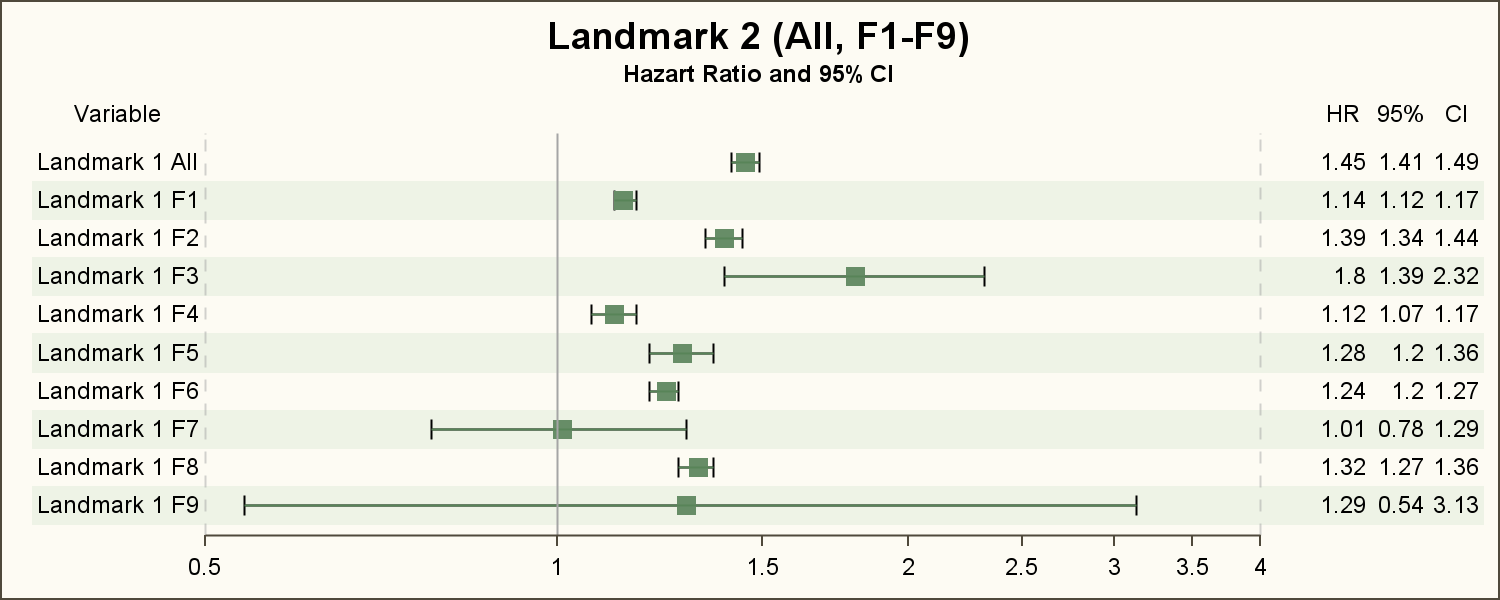


**Supplementary Figure 6.** Landmark 2 disease clusters F1-F9. Subdistributional Hazard Ratio (subHR) for venous thromboembolism in Landmark 2 for cluster F1-F9 according to multimorbidity score ≥2. Reference was one or no disease (score=<2).


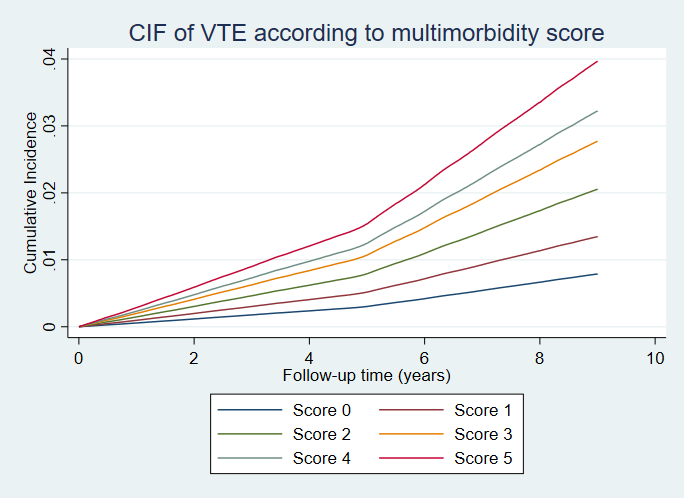

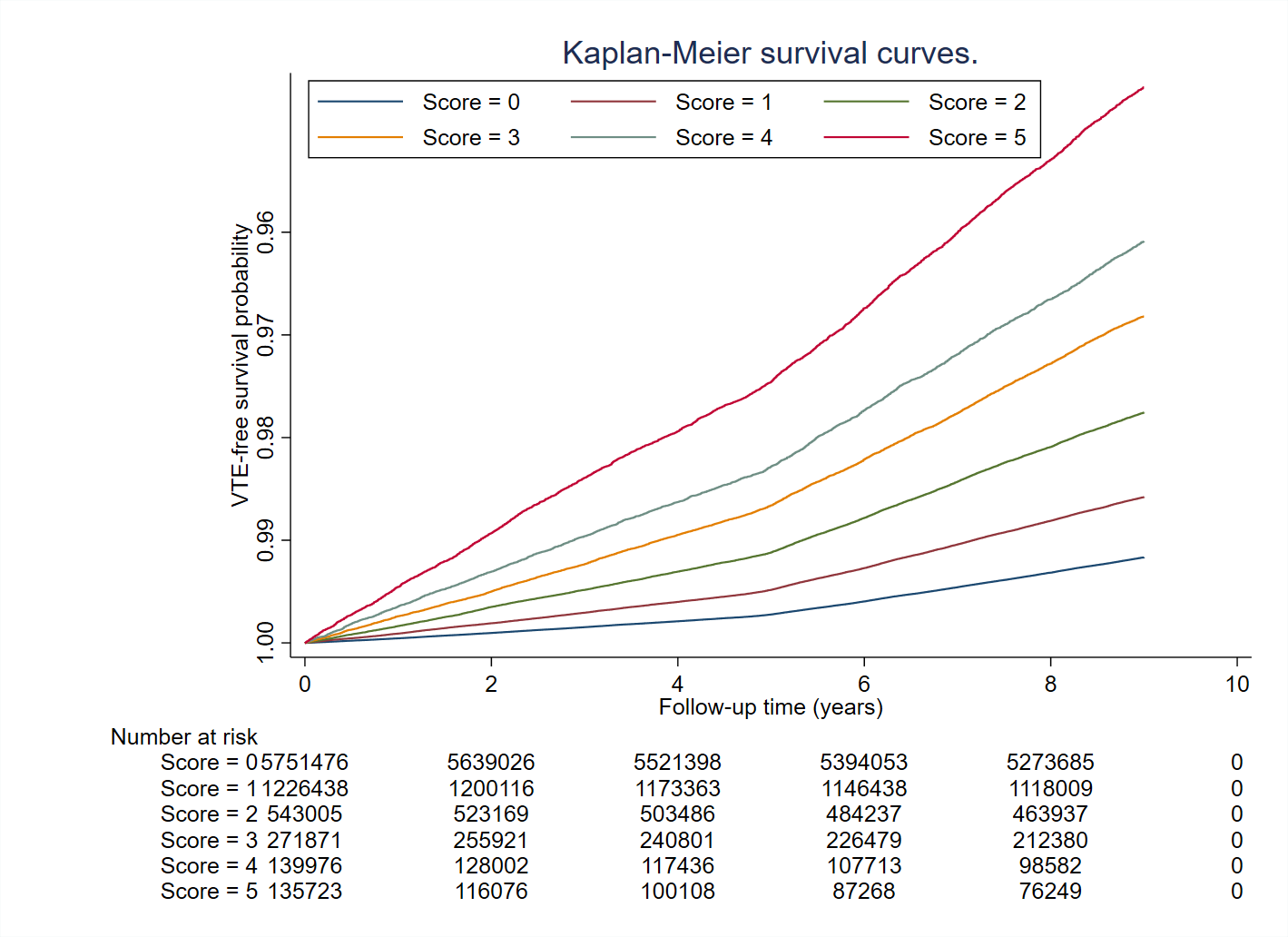


**Supplementary Figure 7.** Landmark 1. Cumulative incidence function (CIF) of Venous thromboembolism (VTE) according to multimorbidity score (score 0-≥5) and Kaplan-Meier estimates for VTE-free survival time according to multimorbidity score (score 0-≥5) in Landmark 2 with 9 years of follow-up time.


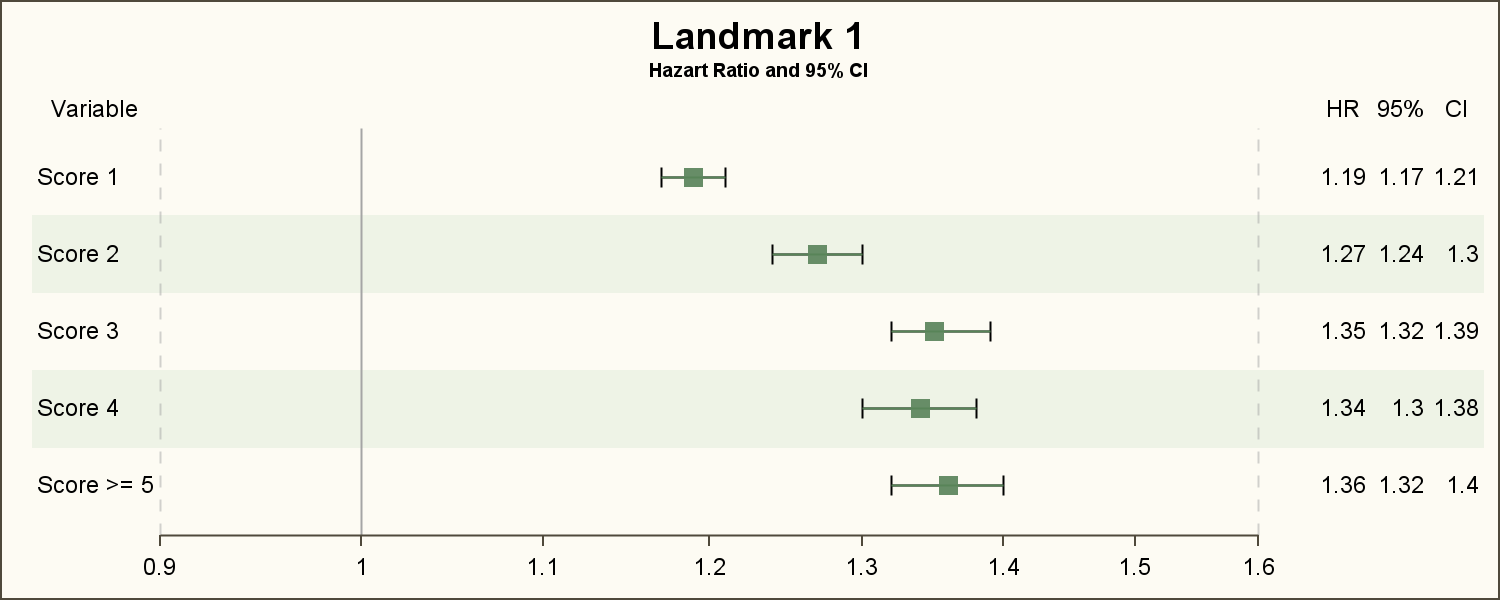


**Supplementary Figure 8.** Landmark 1. Subdistributional Hazard Ratio (subHR) for venous thromboembolism in Landmark 1 according to multimorbidity score 1 - ≥5. Reference was no disease (score=0).


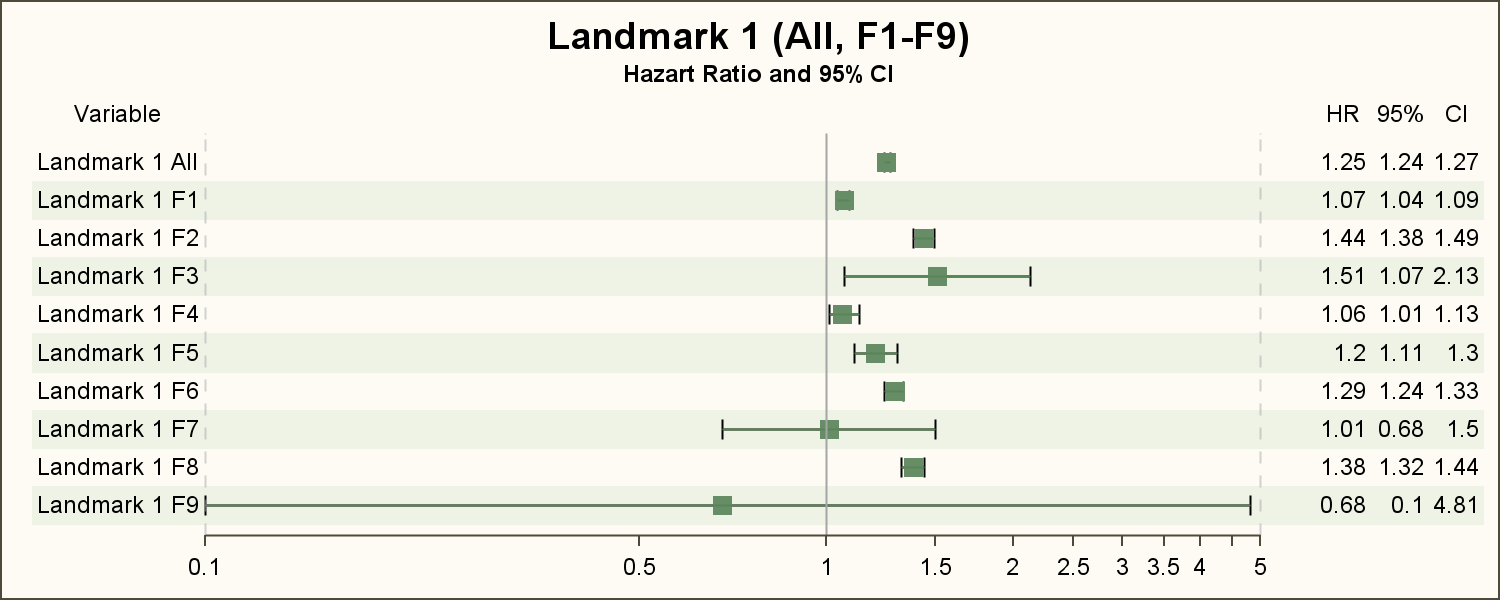


**Supplementary Figure 9.** Landmark 1 disease clusters F1-F9. Subdistributional Hazard Ratio (subHR) for venous thromboembolism in Landmark 1 for cluster F1-F9 according to multimorbidity score ≥2. Reference was one or no disease (score=<2).
